# Signature changes in gut microbiome are associated with increased susceptibility to HIV-1 infection in MSM

**DOI:** 10.1186/s40168-021-01168-w

**Published:** 2021-12-09

**Authors:** Yue Chen, Huang Lin, Mariah Cole, Alison Morris, Jeremy Martinson, Heather Mckay, Matthew Mimiaga, Joseph Margolick, Adam Fitch, Barbara Methe, Vatsala Rangachar Srinivas, Shyamal Peddada, Charles R. Rinaldo

**Affiliations:** 3 Current address: Biostatistics and Bioinformatics Branch, Eunice Kennedy Shriver National Institute of Child Health and Human Development (NICHD), NIH, Bethesda, MD USA; 1 Department of Infectious Diseases and Microbiology, University of Pittsburgh Graduate School of Public Health, Pittsburgh, PA USA; 2 Department of Biostatistics, University of Pittsburgh Graduate School of Public Health, Pittsburgh, PA USA; 4 Present address: Wilmot Cancer Center, University of Rochester Medical Center, Rochester, NY USA; 5 Division of Pulmonary, Allergy and Critical Care Medicine, Department of Medicine, University of Pittsburgh School of Medicine, Pittsburgh, PA USA; 6 Department of Epidemiology, The Johns Hopkins Bloomberg School of Public Health, Baltimore, MD USA; 7 Department of Epidemiology, Fielding School of Public Health, University of California at Los Angeles, Los Angeles, CA USA; 8 Department of Molecular Microbiology and Immunology, The Johns Hopkins Bloomberg School of Public Health, Baltimore, MD USA

**Keywords:** Fecal microbiome, HIV seroconversion, AIDS, MSM (men who have sex with men), Inflammation

## Abstract

**Background:**

Men who have sex with men (MSM) have been disproportionately affected by HIV-1 since the beginning of the AIDS pandemic, particularly in the USA and Europe. Compared to men who have sex with women (MSW), MSM have a distinct fecal microbiome regardless of HIV-1 infection. However, it is unclear whether the MSM-associated gut microbiome affects the susceptibility and progression of HIV-1 infection. We studied fecal microbiome profiles, short-chain fatty acids, and blood plasma inflammatory cytokines of 109 HIV-1 seroconverters (SC) from the early, 1984–1985 phase of the HIV-1 pandemic in the Multicenter AIDS Cohort Study (MACS) before and after HIV-1 infection compared to 156 HIV-1-negative MACS MSM (negative controls [NC]).

**Results:**

We found that family *Succinivibrionaceae*, *S24-7, Mogibacteriaceae, Coriobacteriaceae*, and *Erysipelotrichaceae* were significantly higher (*p*<0.05), whereas *Odoribacteraceae*, *Verucomicrobiaceae*, *Bacteroidaceae*, *Barnesiellaceae*, and *Rikenellaceae* were significantly lower (*p*<0.05), in SC before HIV-1 infection compared to NC. At the species level, *Prevotella stercorea*, *Eubacterium biforme*, and *Collinsella aerofaciens* were significantly higher (*p*<0.05), and *Eubacterium dolichum, Desulfovibrio D168, Alistipes onderdonkii, Ruminococcus torques*, *Bacteroides fragilis, Bacteroides caccae, Alistipes putredinis*, *Akkermansia muciniphila*, *Bacteroides uniformis*, and *Bacteroides ovatus* were significantly lower (*p*<0.05) in SC before HIV-1 infection compared to NC. After HIV-1 infection, family *Prevotellaceae* and *Victivallaceae* and species *Bacteroides fragilis* and *Eubacterium cylindroides* were significantly higher (*p*<0.05) in SC who developed AIDS within 5 years compared to the SC who were AIDS free for more than 10 years without antiretroviral therapy (ART). In addition, family *Victivallaceae* and species *Prevotella stercorea*, *Coprococcus eutactus*, and *Butyrivibrio crossotus* were significantly higher (*p*<0.05) and *Gemmiger formicilis* and *Blautia obeum* were significantly lower (*p*<0.05) after HIV-1 infection in SC who developed AIDS within 5–10 years compared to the SC who were AIDS-free for more than 10 years without ART. Furthermore, plasma inflammatory cytokine levels of sCD14, sCD163, interleukin 6, and lipopolysaccharide binding protein were significantly higher in SC with *p*<0.05 before HIV-1 infection compared to NC.

**Conclusions:**

Our results suggest that pathogenic changes in the gut microbiome were present in MSM several months prior to infection with HIV-1 in the early phase of the AIDS pandemic in the USA. This was associated with increased inflammatory biomarkers in the blood and risk for development of AIDS.

**Video abstract**

**Supplementary Information:**

The online version contains supplementary material available at 10.1186/s40168-021-01168-w.

## Background

The human gastrointestinal (GI) tract possesses gut-associated lymphoid tissue (GALT) which orchestrates host-microbe symbiosis [1]. The gut microbiome also has an important role in maintaining GALT homeostasis and preventing microbial translocation and systemic immune activation [2, 3]. Regardless of the route of HIV-1 infection, the virus quickly moves to GALT and rapidly replicates in part due to the high level of residential activated CD4^+^ T cells. This results in a rapid and severe depletion of CD4^+^ T cells, immune activation, and gut microbiome dysbiosis. These pathogenic changes can lead to microbial translocation, systemic immune activation, and disease progression [4–6].

There are numerous factors that can affect gut microbiome composition, such as genetics, diet, age, antibiotic usage, sex behaviors, and disease status [7–9]. In people with HIV-1 infection (PWH), a number of cross-sectional fecal microbiome studies have shown characteristics of a fecal bacterial community with lower alpha diversity and increased abundance of *Proteobacteria* and *Prevotella* with decreased abundance of *Bacteroidetes*, *Firmicutes*, and *Erysipelotrichaceae* in ART-naïve PWH [5, 6, 10–14]. However, few studies of gut microbiome changes in early HIV-1 infection without ART have been reported due to the challenges of recruitment of such individuals and the advent of pre-exposure prophylaxis. Gori et al. [15] reported that in 57 asymptomatic, antiretroviral treatment (ART)-naive HIV-1-infected Italian men and women, there is an increased abundance of *Pseudomonas aeruginosa* and *Candida albicans*, which is associated with increased microbial translocation, higher viral loads, and lower CD4^+^ T cell percentages, and decreased abundance of *Lactobacilli* and *Bifidobacteria*. Rocafort et al. [16] showed in 49 Mozambican men and women that acute HIV-1 infection led to transient, non-HIV-1-specific changes in gut bacterial richness and composition. Currently, there is no information directly comparing the gut microbiome prior to and soon after HIV-1 infection.

In the USA, men who have sex with men (MSM) have been disproportionately affected by HIV-1 since the early 1980s [17]. Recent studies [18–20] have shown that MSM have a distinct fecal microbiome compared to men who have sex with women (MSW), regardless of HIV-1 infection. In the early years of the HIV-1 pandemic, the greatest risk factor for acquiring HIV-1 in MSM was being the recipient in unprotected anal-receptive intercourse [21, 22]. We hypothesized that the unfavorable interplay of the host gut microbiome and the gut-associated immune system could be a predisposing factor for HIV-1 infection in MSM. Moreover, we further hypothesized the changes of the gut microbiome following HIV-1 infection have an unfavorable impact on HIV-1 disease progression. The answers to these questions could have important implications in the clinical management of acute HIV-1-infected individuals.

In this study, we analyzed longitudinal fecal and blood plasma samples that were obtained from MSM in the Multicenter AIDS Cohort Study (MACS) early in the HIV-1/AIDS pandemic. We explored the changes of the gut microbiome and fecal bacterial product short-chain fatty acids (SCFA) several months before and after documented HIV-1 infection in MSM as well as in HIV-1-negative MSM controls to evaluate the gut microbiome in relation to HIV-1 acquisition and early risk factors for developing AIDS.

## Methods

### Stool and plasma samples

Stool and plasma samples were obtained from the specimen cryorepositories of the MACS (http://aidscohortstudy.org/). The MACS is a prospective cohort study of HIV-1 infection in MSM established in 1983 at 4 sites (Baltimore, Maryland/Washington, DC; Chicago, Illinois; Los Angeles, California; Pittsburgh, Pennsylvania) [23], that has recently joined with the Women’s Interagency HIV Study (WIHS) to form the MACS-WIHS Combined Cohort Study. MACS participants have been studied at semiannual clinic visits with standardized interviews, physical examinations, and phlebotomy for laboratory testing, with storage of plasma and serum and viable peripheral blood mononuclear cells. The study was conducted with institutional review board approval from all participating institutions. During the early phase of the HIV-1 pandemic (1984–1985), MACS participants were instructed to provide stool, urine, semen, and oral wash samples at each clinical research visit, which have been preserved at −80°C without additives or preservatives. Stools were sampled in 20 ml screw-capped glass vials at home and delivered to the clinic within one day by the participants. Enrollment and clinical research of the MACS participants began April 1, 1984, with clinical research visits at 6-month intervals thereafter. During that early period, a number of the MACS participants were infected by HIV-1. HIV-1 seroconversion was determined by enzyme-linked immunosorbent assay and confirmed by Western blot with the participants’ serum samples. The HIV-1 infection date was estimated as the midpoint between the last seronegative and first seropositive clinic research visits [23–25]. In the current study, we examined stool and plasma samples from the clinic research visits flanking the estimated HIV-1 infection time point from 109 HIV-1 seroconverters, and from 156 HIV-1 uninfected MSM controls collected during the same time period. The plasma HIV-1 loads were determined retrospectively by Roche quantitative RT-PCR with a detection limit of 300cp/ml or Roche Ultra-sensitive quantitative RT-PCR with a detection limit of 40cp/ml. The paired samples spanned approximately 6 months, which included an estimated 3 months prior to and 3 months after HIV-1 infection. HIV-1 seroconverters and controls were matched by study centers. All samples were obtained in 1984–1985. Of the 109 HIV-1 seroconverters, 32 developed AIDS [26] within 5 years after seroconversion, 31 developed AIDS within 5–10 years, and 46 were AIDS-free for more than 10 years after seroconversion without ART.

### Profiling microbial populations by sequencing of the variable region of the 16S rRNA gene

Fecal DNA was extracted from the stool samples using the PowerSoil DNA Extraction Kit (MO BIO Laboratories, Carlsbad, CA, USA). The V4 variable region in the 16S rRNA gene was PCR-amplified with the universal primers: 515F 5’-(GTG CCA GCM GCC GCG GTA A)-3’ and 806R 5’-(GGA CTA CHV GGG TWT CTA AT)-3’ [27]. DNA concentrations were measured using Qubit 4 Fluorometer (Thermo Fisher Scientific, Waltham, MA, USA). Amplicons were cleaned, pooled, and sequenced on an Illumina MiSeq platform according to the manufacturer’s specifications to generate paired-end reads. The datasets generated and/or analyzed during the current study are available in the GitHub repository (https://github.com/FrederickHuangLin/Gut-Microbiome-and-HIV-Infection).

### 16S rRNA gene sequence analysis

The resulting 16S rRNA gene sequence data were processed using QIIME2 (version 2019.10.0). The raw sequences were first demultiplexed, and then denoised to remove noisy reads, dereplicated to reduce repetition, and clustered into amplicon sequence variants (ASVs) using the DADA2 algorithm (https://pubmed.ncbi.nlm.nih.gov/27214047/). The observed counts of ASVs were organized into a large matrix referred to as the feature table, where columns represent samples and rows represent ASVs. No ASV was removed based on its observed abundance. The taxonomic composition of bacterial communities was investigated by classifying sequences to the latest reference database (Silva 132 99% OTUs [full-length, seven-level taxonomy, release 132]) using a Naive Bayes classifier.

### Fecal short-chain fatty acid (SCFA) measurement

To determine the concentration of SCFA in the stool samples, 50–100mg fecal matter were aliquoted from every stool sample and sent to the University of Pittsburgh Health Sciences Metabolomics and Lipidomics Core in the Department of Pharmacology & Chemical Biology. The fecal acetate, butyrate, propionate, and valerate levels were measured by stable isotope dilution liquid chromatography mass spectrometry [28].

### Measurement of plasma inflammatory cytokines

For the heparinized plasma samples, the inflammatory cytokines soluble CD14 (sCD14), soluble scavenger receptor CD163 (sCD163), C-reactive protein (CRP), interferon γ-induced protein 10 (IP-10), and lipopolysaccharide-binding protein (LBP) were measured with the Luminex xMAP platform (Luminex, Northbrook, IL, USA), according to the manufacturer’s instructions. The data were collected and analyzed using a BioPlex 200 apparatus and BioPlex Manager Software (Bio-Rad, Hercules, CA, USA). In addition, the inflammatory cytokine interleukin 6 (IL-6) was measured in the plasma samples by ELISA using a commercial ELISA kit (R&D, Minneapolis, MN, USA) following the manufacturer’s instructions.

### Statistical analyses

#### Analysis of gut microbiota composition

The differential abundance (DA) analysis was performed using Analysis of Compositions of Microbiomes with Bias Correction (ANCOM-BC) [29]. The challenge of DA analysis is the compositional nature of microbiome data due to sampling and sequencing depth (the number of reads assigned to an ASV must be interpreted relative to the total number of reads obtained for that sample). ANCOM-BC properly accounts for the compositional nature of the microbiome data by suitably estimating and eliminating the bias introduced by differences in sampling fractions in the observed counts. This methodology uses relative abundances to infer absolute abundances while controlling false discovery rate (FDR) and deals with excess zero by incorporating the ANCOM-II procedure [30]. Microbial absolute abundances were compared between study groups (seroconverters (SC) vs. negative controls (NC)) and different visits (last seronegative visit (visit 1) vs. first seropositive visit (visit 2)) adjusting for age. Furthermore, microbial abundances were also compared in SC based on their years to AIDS diagnosis [26]. Unadjusted *p*-values were used to visualize the results due to a lack of power. Adjusted *p*-values by the Benjamini-Hochberg procedure [31] were also provided in the corresponding supplementary tables.

#### Analysis of gut microbiota’s gene richness and diversity

Both alpha (within-sample) and beta (between-sample) diversities were computed using R microbiome package [32] (Tools for microbiome analysis in R. Version 2.1.24. URL: http://microbiome.github.com/microbiome.) on R Studio (Version 1.2.5033). The calculations of alpha and beta diversities were based on rarefied data (subsample taxa without replacement based on the 90% of minimum library size) since the differential library sizes can have a significant impact on alpha and beta diversities [33].

Inter and intra-study group alpha diversities were evaluated at different visits. Two alpha diversity measurements were investigated: (1) observed number of species or species richness, and (2) Shannon diversity index, which accounts for richness (i.e., the number of species), evenness (i.e., a measure to quantify how equal the microbial abundance is numerical), and divergence (i.e., the variance of species). The corresponding *p*-value was obtained by applying Kruskal-Wallis rank sum test between groups. The log_2_ transformed CD4^+^/CD8^+^ ratio was used as a measure of the immune status of HIV-1-infected participants. Although individually CD4^+^ and CD8^+^ are important, it is their relative value that is biologically more meaningful because these are compositional. Since statistically, log-ratios provide better normal approximation than the ratio, analyses were performed using log_2_ transformed CD4^+^/CD8^+^ ratio. Also note that log-ratio transformation is typically implemented in analyzing compositional data [34]. The relationship between Shannon diversity index and log_2_(CD4^+^/CD8^+^) as well as the relationship between Shannon diversity index and virus load (only for SC at visit 2, log_10_ transformed) were also surveyed. The *p*-value was obtained by fitting a linear regression model between Shannon diversity index, as the outcome variable, and the explanatory variables (log_2_(CD4^+^/CD8^+^) or virus load) and, age stratified by the study group.

Bray-Curtis dissimilarity was selected as the beta diversity measure in this study. *P*-value was obtained by Permutational Multivariate Analysis of Variance (PERMANOVA) (https://onlinelibrary.wiley.com/doi/full/10.1111/j.1442-9993.2001.01070.pp.x). Since a significant PERMANOVA test implies that the observed differences (in multivariate space) are either due to different spatial medians or the heterogeneity of dispersions of different groups, a Permutational Analysis of Multivariate Dispersion (PERMDISP, https://onlinelibrary.wiley.com/doi/pdf/10.1111/j.1541-0420.2005.00440.x?casa_token=C1nt_hAeSVsAAAAA:fyNhMhnZ1V5_CeR0ESbLEuvefJDt5h5E6rFNoFTDE9sUknMBTvqC0FBXMwfT8o2obpKf-VQW4r1SNK6p) test is also performed to confirm the leading effect. An ordination is a popular approach for visualizing and exploring microbial community composition. We projected the samples using principal coordinates (PCoA) plots in the context of Bray-Curtis dissimilarity to visualize between-sample distance.

#### Analysis of SCFA and inflammatory cytokine difference between groups

SCFA levels were investigated between study groups at different visits (NC vs. SC at visit 1; NC vs. SC at visit 2). The *p*-value was obtained by applying Kruskal-Wallis rank sum test between groups. The relationship between SCFA and log_2_(CD4^+^/CD8^+^) as well as the relationship between SCFA and virus load (only for SC at visit 2, log_10_ transformed) were also surveyed. The *p*-value was obtained by fitting a linear regression model between SCFA, as the outcome variable, and the explanatory variables (log_2_(CD4^+^/CD8^+^) or virus load) and age, stratified by the study group. Similar analyses were performed for the inflammatory cytokines.

## Results

### Study participants and clinical samples

**Fig. 1:**
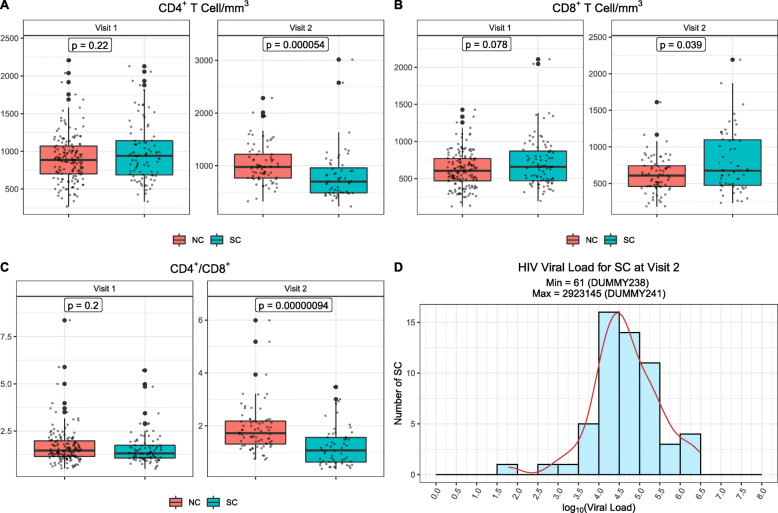
The profiles of CD4^+^ T cell counts (**A**), CD8^+^ T cell counts (**B**), and CD4^+^/CD8^+^ T cell ratio (**C**) of SC and NC at visit 1 and visit 2, and HIV loads of SC (**D**) at visit 2. NC visit 1: *N*=156, visit 2: *N*=77; SC visit 1: *N*=87, visit 2: *N*=57

For this study, the SC and NC were selected and matched from the 4 MACS sites (Baltimore/Wash DC, Chicago, Los Angeles, and Pittsburgh) (Table 1). The HIV-1 infection events in SC were retrospectively reconfirmed by RT-PCR with cryopreserved plasma, with HIV-1 antibody and RNA being negative at visit 1 and both being positive at visit 2 with approximately 6-month intervals. The HIV-1 negativity of NC was confirmed by negative plasma HIV-1 antibody results from both visits. All study participants were self-defined MSM. The SC did not receive ART due to the unavailability of effective antiretroviral drugs in the early years of the HIV-1 pandemic. Stool specimens were obtained from MACS participants beginning April 1, 1984, in order to investigate these specimens for potential viruses that caused AIDS. With the confirmation of HIV-1 as the etiologic agent of AIDS in May, 1984, and prior to the modern resurgence of research on the microbiome, the MACS began to discontinue collection of stools and ended this practice in late 1985. Moreover, not all MACS participants were able to supply stool specimens at each study visit. Thus, within that time, there were 35 SC and 77 NC with plasma and stool samples from both visits 1 and 2, 52 SC and 79 NC only with visit 1 samples, and 22 SC only with visit 2 samples. Compared to NC, SC were younger (*p* = 0.02), but had comparable CD4^+^ T cell counts, CD8^+^ T cell counts, and CD4^+^/CD8^+^ ratios at their pre-HIV-1 infection visit (visit 1). SC had detectable plasma HIV-1 loads, lower CD4^+^ T cell counts, and lower CD4^+^/CD8^+^ ratio at the post-HIV-1 infection, seroconversion visit (visit 2) compared to NC (Fig. 1).

**Table 1:** Characteristics of study participants

	HIV seroconverters (***N***=109)	HIV-negative controls (***N*** = 156)	***P***-value
**Data type**			
Samples from visits 1 & 2 available	35 (32%)	77 (49%)	
Samples from visit 1 available	52 (48%)	79 (51%)	
Samples from visit 2 available	22 (20%)	0 (0%)	<0.01
**Time to AIDS after HIV-1 infection**			
< 5 years	32 (29)	NA	
5–10 years	31 (28)	NA	
> 10 years	46 (42)	NA	NE
**Time period for paired data (days)**			
Min	141	77	
Max	547	230	
Mean (sd)	204.37 ± 63.62	182.60 ± 24.49	0.03
**Age**			
Min	19	21	
Max	82	80	
Mean (sd)	37.12 ± 13.84	42.42 ± 16.64	0.02
**Location**			
Baltimore	22 (20%)	48 (31%)	
Chicago	32 (29%)	39 (25%)	
Pittsburgh	20 (18%)	27 (17%)	
Los Angeles	35 (32%)	42 (27%)	0.28

*NE* not evaluable

### 16S rRNA gene sequencing

A total of 19,500,780 sequence reads were generated for the 377 stool samples, with an average of 51,726 (range 7–126,903) reads per stool sample (Supplementary Figure S1). In the per-base sequence quality plot, the quality of the initial bases appears to be high for both forward and reverse reads, and the quality of the reverse reads appears to drop off around position 170 similar to all Illumina sequencing data.

### Fecal microbiome diversities

#### Alpha diversity

**Fig. 2:**
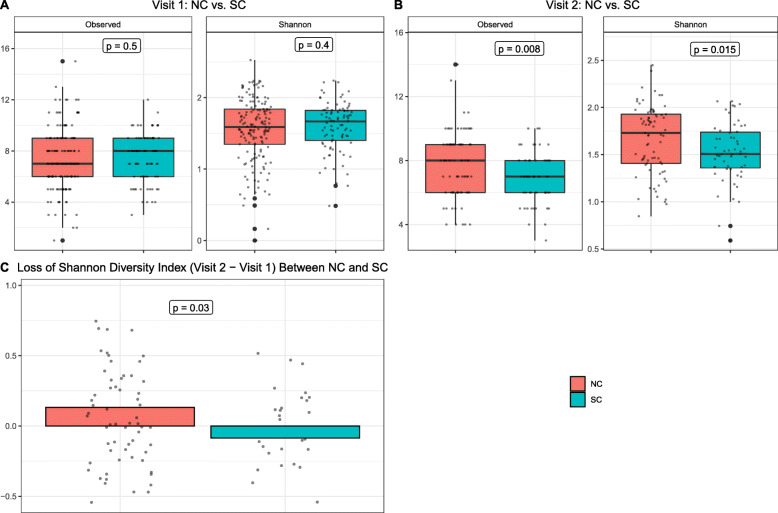
Fecal microbiome alpha diversity at the family level of SC and NC. **A** Alpha diversity of SC and NC at visit 1. **B** Alpha diversity of SC and NC at visit 2. **C** Loss of alpha diversity (Shannon diversity index) of SC and NC at visit 2 compared to alpha diversity at visit 1. Observed: observed species; Shannon: Shannon diversity index

Microbial alpha diversity measures microbial diversity within a sample. Two alpha diversity indices (observed species and Shannon diversity index) were compared at the family level between groups using the standard linear regression method where the *p*-value was adjusted for age. As shown in Fig. 2, there were no differences in observed species and Shannon diversity index between NC and SC at visit 1 (Fig. 2A). However, at visit 2, there were significant decreases of alpha diversity in SC compared to NC for both observed species (*p*=0.008) and Shannon diversity index (*p*=0.015). Furthermore, compared to NC, there was a significant loss of Shannon diversity index (value at visit 2 minus value at visit 1) in SC (*p*=0.03) (Fig. 2C). These results suggest that HIV-1 infection reduced the within-sample bacterial diversity.

#### Correlation analysis of fecal bacterial alpha diversity with CD4^+^/CD8^+^ T cell ratio and HIV-1 load

The peripheral blood CD4^+^/CD8^+^ T lymphocyte ratio is often recognized as a quantitative outcome that reflects the critical role of both CD4^+^ and CD8^+^ T cells in HIV-1 pathogenesis or disease progression [35]. To explore the role of the gut microbiome in HIV pathogenesis, linear regression analysis was performed to reveal the relationship between Shannon diversity index of fecal microbiome and the log_2_ ratio of CD4^+^ T cells and CD8^+^ T cells in peripheral blood mononuclear cells collected at the same stool-collection visits. Interestingly, there was no statistically significant correlation between alpha diversity and CD4^+^/CD8^+^ ratios at visit 1 or visit 2 in both NC and SC (Supplementary Figure S2A and S2B). In addition, no statistically significant correlation was detected between alpha diversity and HIV-1 loads at visit 2 in SC (Supplementary Figure S3).

#### Beta diversity

**Fig. 3:**
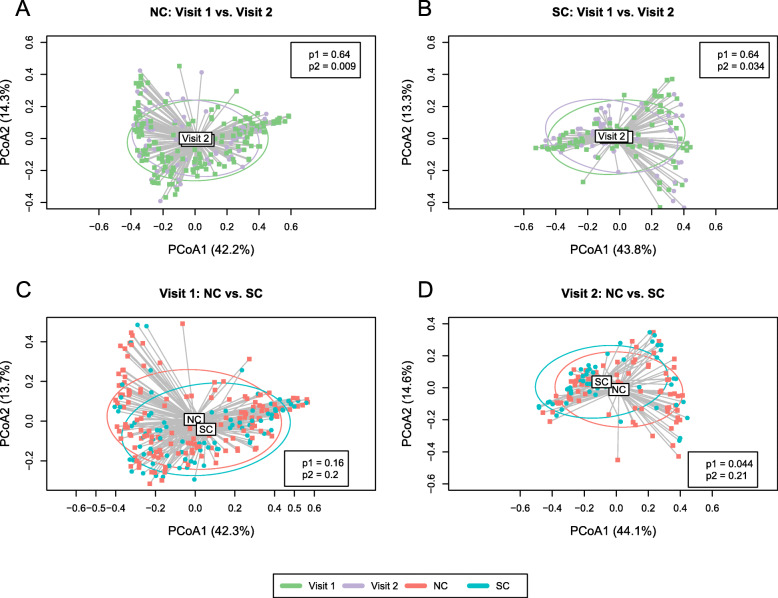
PCoA plot of the fecal microbiome beta diversity (Bray-Curtis dissimilarity) at the family level. Ellipses stand for 68% of data coverage. Lines are connected between samples and the corresponding group spatial median. *P*-values from both PERMANOVA (*p1*) and PERMDISP (*p2*) tests are given in the plot. **A** Beta diversity of visit 1 and visit 2 of NC; **B** Beta diversity of visit 1 and visit 2 of SC; **C** Beta diversity of SC and NC at visit 1; **D** Beta diversity of SC and NC at visit 2

Bacterial beta diversity shows the difference in taxonomic abundance profiles across different groups of samples. In NC or SC, there was no significant difference of beta diversity between visit 1 samples and visit 2 samples based on PERMANOVA test (Fig. 3A, B). Furthermore, PERMANOVA test reveals a trend of separation, but not statistically significant difference, between spatial medians of beta diversity between NC and SC at visit 1 (Fig. 3C). However, comparing visit 2 samples between SC and NC, a statistically significant difference was detected in beta diversity by PERMANOVA test (*p* = 0.044); the PERMDISP test (*p* = 0.21) suggested homogeneity of dispersion and the difference in beta diversity relies on their different spatial medians (Fig. 3D). It is possible that HIV-1 infection led to the changes in the taxonomic abundance profiles of microbial communities in these MSMs.

### Fecal bacterial compositions

**Fig. 4:**
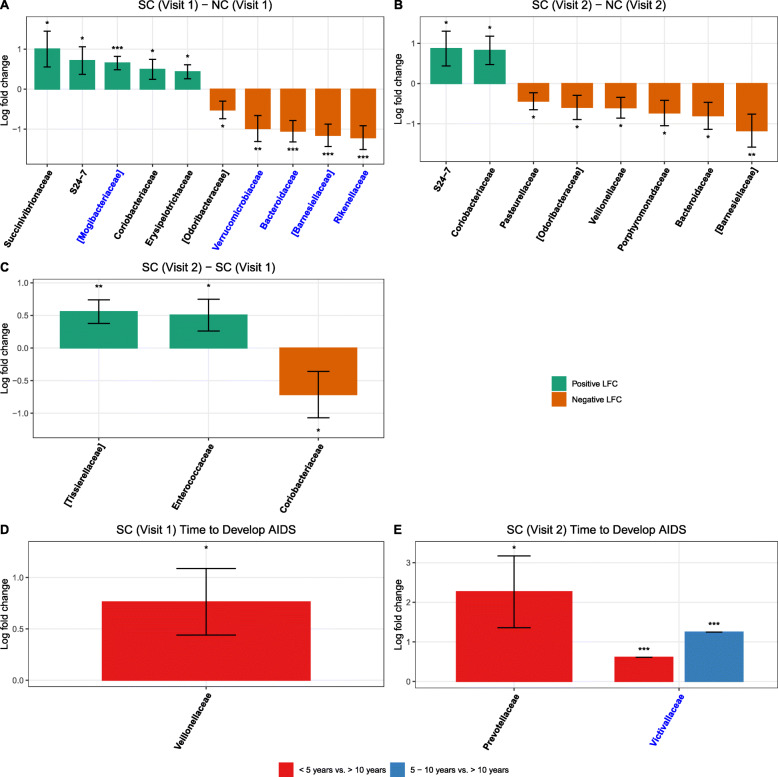
Waterfall plot of log fold change (natural log) of absolute abundances for differentially abundant families. **A** SC vs. NC at visit 1; **B** SC vs. NC at visit 2; **C** visit 2 vs. visit 1 among SC; **D** time to develop AIDS < 5 years/5–10 years vs. > 10 years among SCs at visit 1; **E** time to develop AIDS < 5 years/5–10 years vs. > 10 years among SCs at visit 2. Data are represented by log fold change (shown as a column) ±SE (shown as error bars) derived from the ANCOM-BC model. All effect sizes with *p* < 0.05 are indicated as follows: *significant at 5% level of significance, **significant at 1% level of significance, and ***significant at 0.1% level of significance. Taxa in blue were also significant after multiple testing correction was applied at FDR < 0.05. Exact *p*-values can be found in Supplementary Table 1

Despite certain variations among the groups and visits, there were no significant differences in the taxonomic composition of the fecal microbiome at the phylum level between SC and NC at visit 1 and visit 2 (Supplementary Figure S4). For a deeper understanding of fecal bacterial changes at a lower level of the phylogenic tree, analyses were also performed at the family, genus, and species levels. Compared to NC at visit 1, 5 bacterial families (*Succinivibrionaceae,* S24-7*, Mogibacteriaceae*, *Coriobacteriaceae*, and *Erysipelotrichaceae*) were significantly higher in mean abundance (log fold change [LFC, natural log] ranges from 0.43 to 1, *p*<0.05) and the mean abundance of 5 bacterial families (*Odoribacteraceae*, *Verrucomicrobiaceae*, *Bacteroidaceae*, *Barnesiellaceae*, and *Rikenellaceae*) were significantly lower (LFC ranges from −1.21 to −0.52, *p*<0.05) in SC at visit 1 (Fig. 4A, Supplementary Table 1A). Similarly, two families (S24-7 and *Coriobacteriaceae*) were significantly higher in mean abundance (LFC ranges from 0.83 to 0.87, *p*<0.05) and 6 families (*Pasteurellaceae*, *Odoribacteraceae*, *Veillonellaceae*, *Porphyromonadaceae*, *Bacteroidaceae*, and *Barnesiellaceae*) were significantly lower mean abundance (LFC ranges from −1.17 to −0.44, *p*<0.05) in SC compared to NC at visit 2 (Fig. 4B, Supplementary Table 1B). Compared to SC visit 1, two families (*Tissierellaceae* and *Enterococcaceae*) were significantly higher in mean abundance (LFC ranges from 0.50 to 0.56, *p*<0.05) and one family (*Coriobacteriaceae*) was significantly lower in mean abundance (LFC = −0.71, *p*<0.05) at SC visit 2 after HIV-1 seroconversion (Fig. 4C, Supplementary Table 1C).

At the genus level, compared to NC at visit 1, 17 bacterial genera (*Catenibacterium*, *Mogibacterium*, *Prevotella*, *Succinivibrio*, *Butyrivibrio*, *Peptococcus*, *Bulleidia*, *Desulfovibrio, Eubacterium, Oribacterium, Phascolarctobacterium*, *Collinsella*, *Methanobrevibacter*, *02d06*, *Slackia*, *Anaerovibrio*, and *p-75-a5*) were significantly higher in the mean abundance (LFC ranges from 0.34 to 1.69, *p*<0.05) and 4 bacterial taxa (*Bilophila*, *Akkermansia*, *Bacteroides*, and *Alistipes*) were significantly lower in the mean abundance (LFC ranges from −1.18 to −0.64, *p*<0.05) in SC at visit 1 (Supplementary figure 5A, Supplementary Table 2A). Similarly, 7 genera (*Slackia*, *Mogibacterium*, *Catenibacterium*, *Collinsella*, *Bulleidia*, *Eubacterium*, and *Ruminococcus*) were significantly higher in the mean abundance (LFC ranges from 0.63 to 1.11, *p*<0.05) and 5 genera (*Butyricicoccus*, *Anaerostipes*, *Odoribacter*, *Parabacteroides*, and *Alistipes*) were significantly lower in their mean abundance (LFC ranges from −0.94 to −0.64, *p*<0.05) in SC compared to NC at visit 2 (Supplementary figure 5B, Supplementary Table 2B). Compared to SC visit 1, three genera (*Paraprevotella*, *Methanosphaera*, and *Defluviitalea*) were significantly higher in their mean abundance (LFC ranges from 0.34 to 0.84, *p*<0.05), and one genus (*Collinsella*) was significantly lower (LFC = −0.78, *p*<0.05) at SC visit 2 after HIV-1 seroconversion (Supplementary figure 5C, Supplementary Table 2C).

**Fig. 5:**
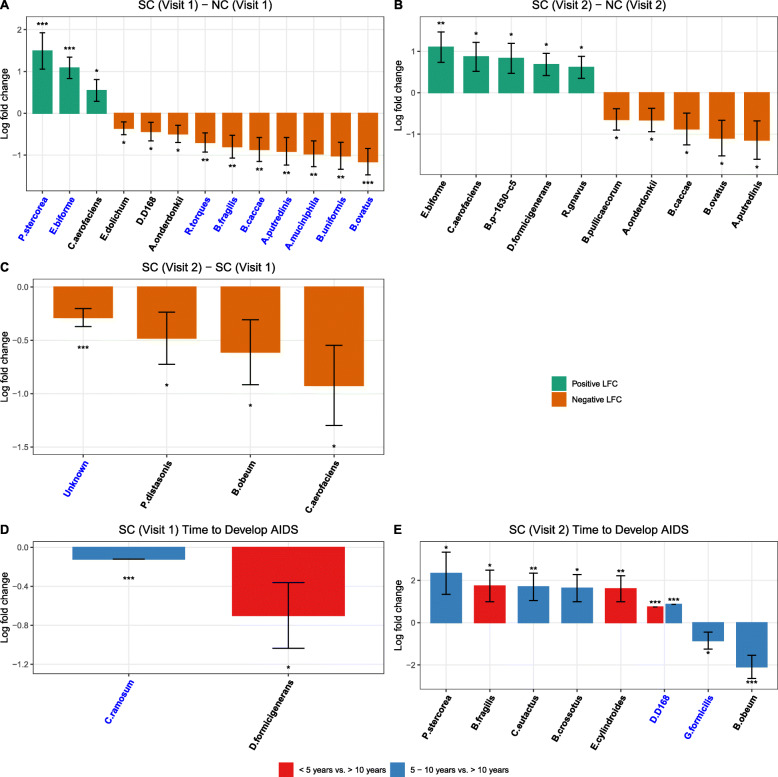
Waterfall plot of log fold change (natural log) of absolute abundances for differentially abundant species. **A** SC vs. NC at visit 1, **B** SC vs. NC at visit 2, **C** visit 2 vs. visit 1 among SC, **D** time to develop AIDS < 5 years/5–10 years vs. > 10 years among SCs at visit 1, and **E** time to develop AIDS < 5 years/5–10 years vs. > 10 years among SCs at visit 2. Data are represented by log fold change (shown as a column) ±SE (shown as error bars) derived from the ANCOM-BC model. All effect sizes with *p* < 0.05 are indicated as follows: *significant at 5% level of significance, **significant at 1% level of significance, and ***significant at 0.1% level of significance. Taxa in blue were also significant after multiple testing correction was as applied at FDR < 0.05. Exact *p*-values can be found in Supplementary Table 3

At the species level, compared to NC at visit 1, three bacterial species (*Prevotella Stercorea*, *Eubacterium Biforme*, and *Collinsella Aerofaciens*) were significantly higher in the mean abundance (log fold change [LFC, natural log] ranges from 0.55 to 1.49, *p*<0.05) and 10 bacterial species (*Eubacterium Dolichum*, *Desulfovibrio D168*, *Alistipes Onderdonkii*, *Ruminococcus Torques*, *Bacteroides Fragilis*, *Bacteroides Caccae*, *Alistipes Putredinis*, *Akkermansia Muciniphila*, *Bacteroides Uniformis*, *and Bacteroides Ovatus*) were significantly lower in the mean abundance (LFC ranges from −1.16 to −0.36, *p*<0.05) in SC at visit 1 (Fig. 5A, Supplementary Table 3A). Similarly, 5 bacterial species (*Eubacterium biforme*, *Collinsella aerofaciens*, *Bulleidia p-1630-c5*, *Dorea formicigenerans*, and *Ruminococcus gnavus*) were significantly higher in the mean abundance (LFC ranges from 0.61 to 1.10, *p*<0.05) and 5 bacterial species (*Butyricicoccus Pullicaecorum*, *Alistipes Onderdonkii*, *Bacteroides Caccae*, *Bacteroides Ovatus*, and *Alistipes Putredinis*) were significantly lower in the mean abundance (LFC ranges from −1.14 to −0.65, *p*<0.05) in SC compared to NC at visit 2 (Fig. 5B, Supplementary Table 3B). Compared to SC visit 1, 4 species (*unknown*, *Parabacteroides distasonis*, *Blautia obeum*, and *Collinsella aerofaciens*) were significantly lower in the mean abundance (LFC ranges from −0.92 to −0.29, *p*<0.05) at SC visit 2 after HIV-1 seroconversion (Fig. 5C, Supplementary Table 3C). Since HIV-1 replicates rampantly in gut-associated lymphoid tissue and compromises the mucosal immune system during acute HIV-1 infection [36], the changes of bacterial taxa after HIV-1 infection in SC suggest a pathogenic impact of HIV-1 infection on the gut microbiome.

The bacterial composition of SCs was further analyzed according to the time to AIDS diagnosis. We discovered that *Veillonellaceae* was significantly higher (*p*<0.05) in the mean abundance at visit 1 in SC with AIDS development within 5 years compared to the SC who were AIDS-free for more than 10 years (Fig. 4D, Supplementary Table 1D). At visit 2, *Prevotellaceae* was significantly higher (*p*<0.05) in the mean abundance among the SC who developed AIDS within 5 years compared to the SC who were AIDS-free for more than 10 years. Additionally, *Victivallaceae* was only present in the SC with AIDS development within 5 years or 5–10 years compared to the SC who were AIDS-free for more than 10 years (Fig. 4E, Supplementary Table 1E). At the genus level, *Methanosphaera* was significantly higher (*p*<0.05) and *Eubacterium* was significantly lower at visit 1 in SC with AIDS development within 5 years compared to the SC who were AIDS-free for more than 10 years (Supplementary Figure S5D, Supplementary Table 2D). At visit 2, the mean abundance of *Prevotella* was significantly higher whereas *Faecalibacterium*, *Anaerovibrio*, and *Paraprevotella* had significantly lower mean abundance (*p*<0.05, *Anaerovibrio* was absent among AIDS rapid developers) in the SC who developed AIDS within 5 years compared to the SC who were AIDS-free for more than 10 years. Additionally, at visit 2, *Mitsuokella, Butyrivibrio, Oribacterium,* and *Lachnospira* were significantly higher and *Blautia* and *Gemmiger* were significantly lower in mean abundance (*p*<0.05) in the SC with AIDS development within 5–10 years compared to the SC who were AIDS-free for more than 10 years (Supplementary Figure S5E, Supplementary Table 2E). At the species level, *Dorea formicigenerans* was significantly lower in mean abundance (*p*<0.05) at visit 1 in SC with AIDS development within 5 years compared to the SC who were AIDS-free for more than 10 years. Additionally, *Clostridium ramosum* was absent in the SC with AIDS development within 5–10 years compared to the SC who were AIDS-free for more than 10 years (Fig. 5D, Supplementary Table 3D). At visit 2, *Bacteroides fragilis, Eubacterium cylindroides,* and *Desulfovibrio D168* were significantly higher in mean abundance (*p*<0.05, where *Desulfovibrio D168* is absent among AIDS long-term developers) in the SC who developed AIDS within 5 years compared to the SC who were AIDS-free for more than 10 years. Additionally, *Prevotella stercorea*, *Coprococcus eutactus*, *Butyrivibrio crossotus*, and *Desulfovibrio D168* were significantly higher (*p*<0.05, *Desulfovibrio D168* is absent among AIDS long-term developers) and *Gemmiger formicilis* and *Blautia obeum* were significantly lower in mean abundance (*p*<0.05) at visit 2 in the SC with AIDS development within 5–10 years compared to the SC who were AIDS-free for more than 10 years (Fig. 5, Supplementary Table 3E).

### Fecal SCFA

**Fig. 6:**
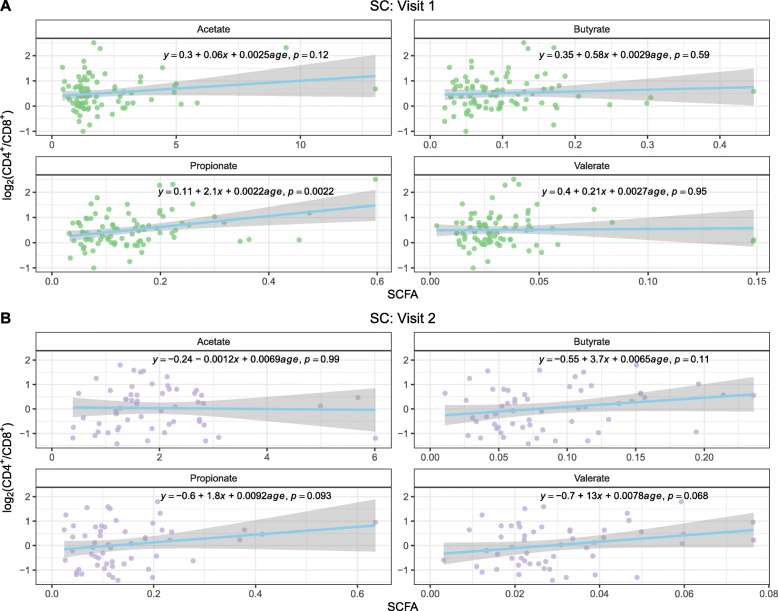
Correlation of fecal SCFA levels and log_2_ ratio of the peripheral blood CD4^+^/CD8^+^ at visit 1 (**A**) or visit 2 (**B**) of SC. SC visit 1: *N*=87, visit 2: *N*=57

Gut microbes produce SCFA by fermenting dietary non-digestible carbohydrates. SCFA are important for intestinal and immune homeostasis. Unbalanced intestinal SCFA quantities play a role in HIV-1 pathogenesis [37–39]. Therefore, four SCFA (acetate, butyrate, propionate, and valerate) were measured in the stool samples of both NC and SC at both visits. Although we did not find significant differences in the level of each individual SCFA between NC and SC at visit 1 or visit 2 (data not shown), analysis of SCFA level and CD4^+^/CD8^+^ ratios (Fig. 6) showed there was a significant positive correlation between the propionate levels and CD4^+^/CD8^+^ ratio at visit 1 of SC with *p* = 0.0022 (Fig. 6A), and a trend of a positive correlation with *p* = 0.093 at visit 2 in SC (Fig. 6B). Interestingly, there was no significant correlation between propionate levels and CD4^+^/CD8^+^ ratio in NC in either visit (data not shown). Correlations between SCFA and plasma inflammatory cytokine levels at both visits were not statistically significant (data not shown). Correlations between SCFA levels and HIV-1 load at visit 2 in SC were not found to be significant (Supplementary Figure S6). Propionate, the conjugate base of propionic acid, is produced by gut bacteria *Bacteroidetes*, *Firmicutes*, and others. Propionate plays an important role in gut immune regulation. The positive association of propionate level and CD4^+^/CD8^+^ ratio before HIV-1 infection could indicate propionate’s role in HIV-1 acquisition due to the high level of CD4^+^ T cells.

### Plasma inflammatory cytokines

**Fig. 7:**
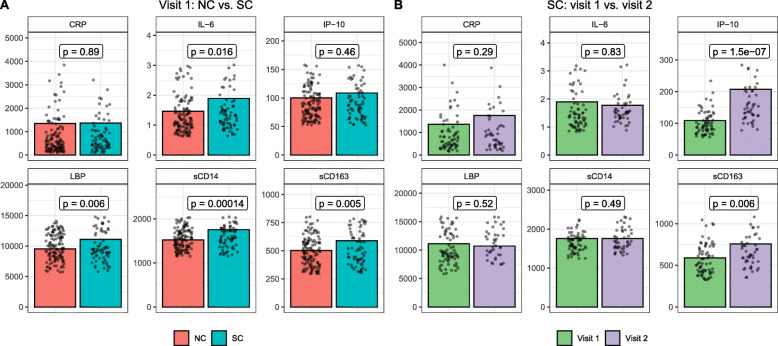
**A** Relative abundances of peripheral blood inflammatory cytokines at visit 1 between NC (*N*=156) and SC (*N*=87). **B** Relative abundances of peripheral blood inflammatory cytokines between visit 1 (*N*=87) and visit 2 (*N*=57) of SC. The measurement for each cytokine is IL-6 (pg/ml), sCD163 (ng/ml), IP-10 (pg/ml), CRP (ng/ml), LBP (ng/ml), and sCD14 (ng/ml). For visualization purposes, scatter points with values belonging to the first/last 10 percentile were not shown

**Fig. 8:**
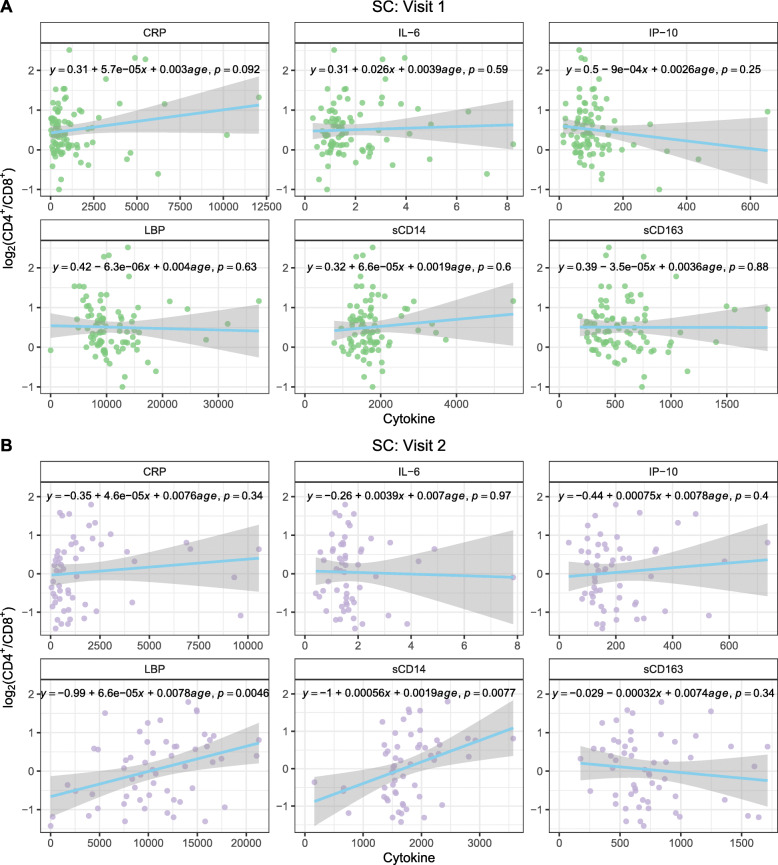
Correlation of peripheral blood inflammatory cytokines and log_2_ ratio of CD4^+^/CD8^+^ at visit 1 (**A**) and visit 2 (**B**) of SC

**Fig. 9:**
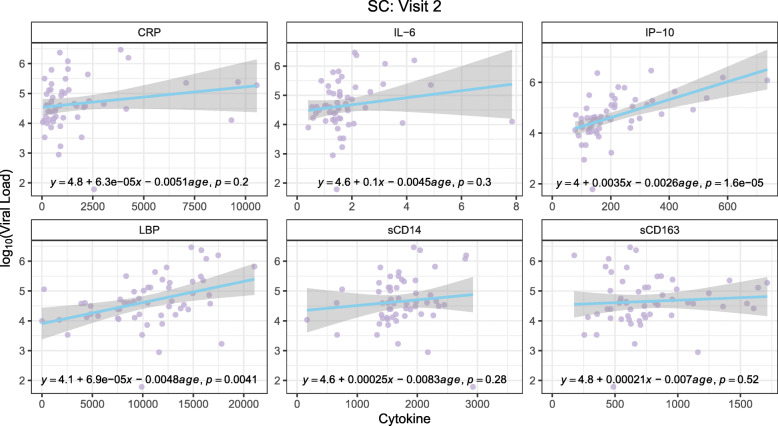
Correlation analysis of peripheral blood inflammatory cytokines and log_10_ HIV loads at visit 2 of SC

Dynamic changes of pro-inflammatory cytokines in the peripheral blood play an important role in HIV-1 acquisition and disease progression [40]. There was an effect of inflammation on the incidence of seroconversion to HIV-1, as the levels of plasma sCD14, sCD163, IL-6, and LBP were significantly higher prior to seroconversion at visit 1 in SC compared to visit 1 in NC (Fig. 7A). After seroconversion to HIV-1, levels of sCD163 and IP10 significantly increased in SC at visit 2 compared to pre-seroconversion visit 1 (Fig. 7B). None of the cytokine levels significantly correlated with CD4^+^/CD8^+^ ratio among NC at either visit (Supplementary Figure S7) or among SC at visit 1 (Fig. 8A), with the exception of IL-6 and CRP at NC visit 1. However, these positive correlations are likely driven by certain leverage points (observations for which the cytokine level is much higher than average). On the contrary, the levels of sCD14 and LBP at visit 2 among SC were positively correlated with CD4^+^/CD8^+^ ratio (*p*=0.0077 and *p*=0.0046, respectively) (Fig. 8B). Levels of IP-10 and LBP at visit 2 among SC positively correlated with HIV-1 viral load with *p*-values <0.01 (Fig. 9).

### Correlation of microbiome before HIV-1 infection and HIV-1 seroconversion status and pathogenesis

The role of the fecal microbiome in the HIV-1 susceptibility of MSM is not clear due to the challenges of collecting fecal materials before HIV-1 infection. In this study, we took the unique opportunity to analyze the fecal microbiome composition before HIV-1 infection and its implication in HIV-1 acquisition and pathogenesis post-HIV-1 infection in MSM.

#### Microbiome alpha diversity at visit 1 and CD4^+^/CD8^+^ at visit 2

To explore the role of fecal bacterial within-sample diversity prior to HIV-1 infection in acute HIV-1 infection pathogenesis, Shannon diversity index at visit 1 was co-analyzed with peripheral blood CD4^+^/CD8^+^ ratio at visit 2 in SC and NC. As shown in Supplementary Figure S8, although it was not statistically significant, fecal bacterial alpha diversity at visit 1 tended to be negatively correlated with CD4^+^/CD8^+^ ratio at visit 2 in SC, but not in NC. As the baseline diversity increases, it appears that the CD4^+^/CD8^+^ ratio decreases dramatically post-HIV-1 infection for SC.

## Discussion

The basic premise of our study is that the fecal microbiome has a major effect on the infection of MSM with HIV-1. This is based on known differences in enteric microbiota and systemic immune activation in non-HIV-1 infected MSM [19, 41], and in MSM with chronic HIV-1 infection [42–45]. Moreover, recent studies [19, 41, 46, 47] have shown that MSM have distinct microbiomes compared to MSW regardless of HIV-1 infection status. In addition, CD4^+^ T cells with high expression of HIV-1 co-receptor CCR5 have been detected in MSM rectosigmoid colon biopsies [48]. The impact of the fecal microbiome on HIV-1 infection is also evident in changes in uninfected individuals taking pre-exposure prophylaxis (PrEP) to prevent HIV-1 infection [49], and HIV-1-infected individuals with ART [50–54] or without ART [54, 55]. The non-human primate model also supports that a fecal microbiome with lower ratios of Bacteroides to Prevotella, and lower levels of Firmicutes are associated with mucosal transmission of high levels of simian-human immunodeficiency virus [56]. However, there is no information on whether these differences in the microbiome are linked to susceptibility to HIV-1 infection in MSM.

To address this issue, we applied unique data and biologic specimens from the earliest phase of the AIDS pandemic in MSM enrolled in the MACS to describe for the first time the dynamic changes of the gut microbiome, plasma inflammatory cytokines, and the peripheral blood CD4^+^/CD8^+^ T cell ratio present several months before and after primary HIV-1 infection in MSM. We found that there were significant differences in the fecal microbiome compositions of SC and NC in our MSM cohort at their biannual study visit prior to HIV-1 infection. Our findings after HIV-1 infection in SC are in agreement with existing literature on high levels of *Prevotella stercorea* [13, 42, 46], *Eubacterium biforme* [13, 46] and low levels of *Desulfovibrio D168* [19], *Bacteroides fragilis* [57], *Bacteroides caccae* [13, 46, 57], *Akkermansia muciniphila* [46, 58, 59], *Bacteroides uniformis* [13, 42, 46, 57], and *Bacteroides ovatus* [13, 46, 57] in PWH. Especially, significantly higher in the abundance of *Prevotella stercorea* (*p*<0.01, BH adjusted *p*=0.01) and lower in the abundance of *Bacteroides fragilis* (*p*<0.01, BH adjusted *p*=0.02), *Bacteroides caccae* (*p*<0.01, BH adjusted *p*=0.02), *Bacteroides uniformis* (p<0.01, BH adjusted *p*=0.02), and *Bacteroides ovatus* (*p*<0.01, BH adjusted *p*=0.01) were detected in SC before HIV-1 infection compared to NC. These data support that the pathogenic microbiome changes with high *Prevotella* and low *Bacteroides* occurred before HIV-1 infection in MSM. Although high levels of fecal *Prevotella* were reported in healthy Tanzania hunter-gatherers [60] and athletes [61], increases in fecal *Prevotella* and decreases in *Bacteroides* have also been found in various gut inflammatory conditions [62–64]. Our finding could indicate a pro-inflammatory condition in the GI tract in SC before acquiring HIV-1, in that *P. stercorea* species of the gut commensal bacterial genus *Prevotella* has pro-inflammatory effects, whereas several species of the genus *Bacteroides* have the anti-inflammatory function by promoting T regulatory cell reactivity [62, 65]. Pascal et al. [63] reported that fecal bacteria family *Mogibacteriaceae* along with other bacteria were significantly increased in inflammatory bowel disease (IBD) patients with proctitis. Similarly, the fecal bacteria family *Erysipelotrichaceae* is associated with HIV-1 infection [13, 66] and colorectal cancer [67, 68]. In our study participants, two potential pathogenic fecal bacteria, *Mogibacteriaceae* and *Erysipelotrichaceae*, were significantly increased in SC prior to HIV-1 infection compared to NC. In contrast, anti-inflammatory and protective bacteria, *Bacteroidaceae* and *Rikenellaceae*, whose decrease is associated with HIV-1 infection and IBD [69–71], were significantly lower in SC prior to HIV-1 infection. These changes of microbiome composition in MSM before HIV-1 seroconversion could lead to greater inflammation in the GI tract and position them to be vulnerable to HIV-1 infection.

SCFA are fermentation products of gut microbiota from dietary fibers and a major factor in the maintenance of gut and immune homeostasis [72, 73]. Rau et al. [74] reported the association of higher fecal propionate and acetate levels with lower resting regulatory T cells and higher Th17/rTreg ratio in the peripheral blood in nonalcoholic fatty liver disease patients. In this study, we found a significant positive correlation between the levels of propionate and the peripheral blood CD4^+^/CD8^+^ ratio before HIV-1 infection in SC. Although limited reports are available on propionate level changes in PWH [75] and in other diseases [76], whether the gut propionate level modulates the systemic immune system and influences HIV-1 susceptibility needs further investigation.

Immune activation after HIV-1 infection has been extensively reported [77–79]. However, there are very limited reports on immune turbulence surrounding HIV-1 infection, which is important for early therapeutic intervention. Breen et al. [80] reported that the significant changes of plasma cytokines 20 days before and 20 days after HIV-1 infection of 3 acute HIV-1-infected persons, showing high levels of plasma pro-inflammatory cytokines a few days post HIV-1 infection. In our current study, significantly high levels of pro-inflammatory cytokine sCD14, sCD163, IL-6, and LBP were detected in SC prior to HIV-1 infection compared to NC (*p*<0.05). This systemic immune activation could be the consequence of microbial translocation caused by fecal microbiome dysbiosis, which was further supported by the significant increase in pro-inflammatory bacterial taxa (*Prevotella stercorea*) and a significant decrease in anti-inflammatory bacterial taxa (*Bacteroides fragilis, Bacteroides caccae, Bacteroides uniformis*, and *Bacteroides ovatus*) in SC prior to HIV-1 infection. These microbiome changes could increase local inflammation and mucosal membrane permeability, which may in turn lead to microbial translocation and systemic inflammation. In addition, this microbiome change could increase the number of local activated CD4^+^ T cells by immune activation and chemoattractant, which then serve as targets upon exposure to HIV-1 in the GI tract.

To our knowledge, our study is the only investigation linking the fecal microbiome present within several months before and after primary HIV-1 infection with the development of AIDS. After HIV-1 infection, we found that fecal *Prevotellaceae*, *Victivallaceae*, and *Eubacterium cylindroides* were significantly higher in SC who subsequently developed AIDS faster (within 5 years) compared to the SC who were AIDS-free for more than 10 years without ART. High levels of fecal *Prevotellaceae* and *Eubacterium cylindroides* are found in PWH [13, 46, 57], and fecal *Victivallaceae* are enriched in PWH on ART after nutritional intervention [81]. We recognize that given the relatively small number of PWH in our study for prediction modeling, caution is warranted in making predictive conclusions on the role of the fecal microbiome in developing AIDS. Nevertheless, it is unlikely that our study can be reproduced due to the lack of stool specimens archived from other pre-ART, HIV/AIDS cohort studies and current treatment policies of rapidly initiating ART soon after primary HIV-1 infection.

The present results are pertinent to recent studies showing a distinct gut microbiome composition in MSM compared to MSW even in the absence of HIV-1 infection [18, 19, 41, 46]. Furthermore, Li et al. reported that mice receiving MSM stool samples showed increased activation of CD4^+^ T cells, and human gut-derived immune cells were more likely to be infected by HIV-1 after being exposed to MSM fecal bacteria in vitro [18]. Although all of our study participants were MSM, different homosexual behaviors of individual study participants might contribute to different levels of microbiome changes and local and systemic inflammation. Indeed, men who are exclusively receptive during anal intercourse are more likely to become infected with HIV-1 than exclusively insertive men [22, 82]. It is possible that the microbiome differences observed in SC before HIV-1 infection are caused by different sexual behaviors of the study participants. Furthermore, since the fecal microbiome plays an important role in modulating the systemic immune system, the microbiome differences observed could be a driving factor for immune activation and susceptibility to HIV-1 infection. After HIV-1 infection, fecal alpha diversity of SC significantly decreased, which is consistent with a previous study by Noguera-Julian et al. [19]. Further investigation of the role of sexual history in the microbiome of our study participants is ongoing.

## Conclusions

Our results support that pathogenic changes in the gut microbiome occurred in MSM several months prior to seroconversion to HIV-1. This was associated with increased inflammatory biomarkers in blood and increased risk for the development of AIDS.

## Supplementary Information

**Additional file 1.** Supplementary figure S1. Sequence reads of bacterial 16S rRNA V4 gene of SC and NC stool samples. Supplementary figure S2. Correlation analysis of fecal microbiome Shannon diversity index and log_2_ ratio of peripheral blood CD4^+^/CD8^+^ at visit 1(A) or visit 2(B) of SC and NC. Supplementary figure S3. Correlation analysis of fecal microbiome Shannon diversity index and peripheral blood HIV loads at visit 2 of SC. Supplementary figure S4. The fecal microbiome compositions at phylum level of SC and NC at visit 1 and visit 2 obtained with 16S rRNA gene sequencing. Supplementary figure S5. Waterfall plot of log fold change (natural log) of absolute abundances for differentially abundant genera. A: SC vs. NC at visit 1; B. SC vs. NC at visit 2; C. visit 2 vs. visit 1 among SC; D. Time to develop AIDS < 5 years/5-10 years vs. > 10 years among SCs at visit 1; E. Time to develop AIDS < 5 years/5-10 years vs. > 10 years among SCs at visit 2. Data are represented by log fold change (shown as column) ±SE (shown as error bars) derived from the ANCOM-BC model. All effect sizes with p < 0.05 are indicated, *significant at 5% level of significance; **significant at 1% level of significance; ***significant at 0.1% level of significance. Taxa in blue were also significant after multiple testing correction was applied at FDR < 0.05. Exact p-values can be found in Supplementary Table 2. Supplementary figure S6. Correlation analysis of fecal SCFAs and log_10_ peripheral blood HIV loads at visit 2 of SC. Supplementary figure S7. Correlation of peripheral blood inflammatory cytokines and log_2_ ratio of CD4^+^/CD8^+^ at visit 1 (A) and visit 2 (B) of NC. Supplementary figure S8. Correlation of fecal microbiome alpha diversity (Shannon diversity index) at visit 1 and log2 ratio of peripheral blood CD4+/CD8+ at visit 2 of SC and NC.

**Additional file 2.** Supplementary Table 1. The log fold change (LFC) of absolute abundances for differentially abundant families (A), (B), (C), (D), (E). *The taxon was declared to be significant since the absolute abundance in the reference group (Time to Develop AIDS > 10 Years) was zero. **NE: not evaluable. Supplementary Table 2. The log fold change (LFC) of absolute abundances for differentially abundant genera (A), (B), (C), (D), (E). *The taxon was declared to be significant since the absolute abundance in the group of interest (Time to Develop AIDS < 5 Years) was zero. **NE: not evaluable. Supplementary Table 3. The log fold change (LFC) of absolute abundances for differentially abundant families (A), (B), (C), (D), (E). *The taxon was declared to be significant since the absolute abundance in the group of interest (Time to Develop AIDS 5 - 10 Years) was zero. **NE: not evaluable. ***The taxon was declared to be significant since the absolute abundance in the reference group (Time to Develop AIDS > 10 Years) was zero.

## Data Availability

The datasets generated and/or analyzed during the current study are available in the GitHub repository (https://github.com/FrederickHuangLin/Gut-Microbiome-and-HIV-Infection).

## Abbreviations

MSM: Men who have sex with men
HIV-1: Human immunodeficiency virus type 1
MACS: Multicenter AIDS Cohort Study
SC: HIV-1 seroconverters
NC: HIV-1 negative controls
SCFA: Short-chain fatty acids
ART: Antiretroviral drug therapy
