# Supplementary figures and images for: Signature changes in gut microbiome are associated with increased susceptibility to HIV-1 infection in MSM
Source: Microbiome. 2021 Dec 9;9:237. doi: 10.1186/s40168-021-01168-w (PMC8656045; doi:10.1186/s40168-021-01168-w)

# Reads per sample

Min = 7 (F-132)

Max = 126903 (F-141)

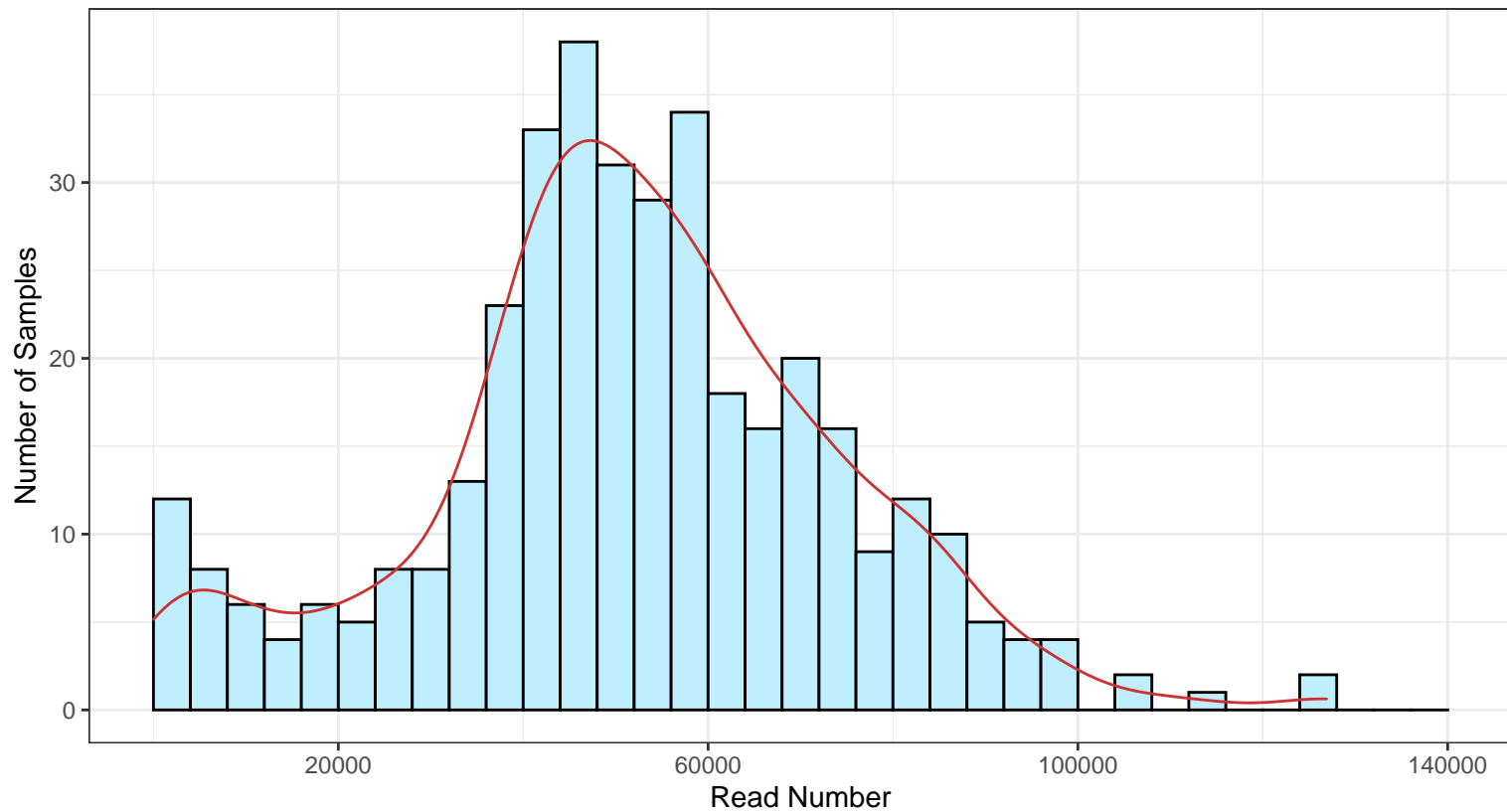

Supplement: Supplementary file 2 — Additional file 1. Supplementary figure S1. Sequence reads of bacterial 16S rRNA V4 gene of SC and NC stool samples. Supplementary figure S2. Correlation analysis of fecal microbiome Shannon diversity index and log2 ratio of peripheral blood CD4+/CD8+ at visit 1(A) or visit 2(B) of SC and NC. Supplementary figure S3. Correlation analysis of fecal microbiome Shannon diversity index and peripheral blood HIV loads at visit 2 of SC. Supplementary figure S4. The fecal microbiome compositions at phylum level of SC and NC at visit 1 and visit 2 obtained with 16S rRNA gene sequencing. Supplementary figure S5. Waterfall plot of log fold change (natural log) of absolute abundances for differentially abundant genera. A: SC vs. NC at visit 1; B. SC vs. NC at visit 2; C. visit 2 vs. visit 1 among SC; D. Time to develop AIDS < 5 years/5-10 years vs. > 10 years among SCs at visit 1; E. Time to develop AIDS < 5 years/5-10 years vs. > 10 years among SCs at visit 2. Data are represented by log fold change (shown as column) ±SE (shown as error bars) derived from the ANCOM-BC model. All effect sizes with p < 0.05 are indicated, *significant at 5% level of significance; **significant at 1% level of significance; ***significant at 0.1% level of significance. Taxa in blue were also significant after multiple testing correction was applied at FDR < 0.05. Exact p-values can be found in Supplementary Table 2. Supplementary figure S6. Correlation analysis of fecal SCFAs and log10 peripheral blood HIV loads at visit 2 of SC. Supplementary figure S7. Correlation of peripheral blood inflammatory cytokines and log2 ratio of CD4+/CD8+ at visit 1 (A) and visit 2 (B) of NC. Supplementary figure S8. Correlation of fecal microbiome alpha diversity (Shannon diversity index) at visit 1 and log2 ratio of peripheral blood CD4+/CD8+ at visit 2 of SC and NC. [file 40168_2021_1168_MOESM2_ESM.zip › fig_s1_ESM.pdf]

**A**

Visit 1: NC vs. SC

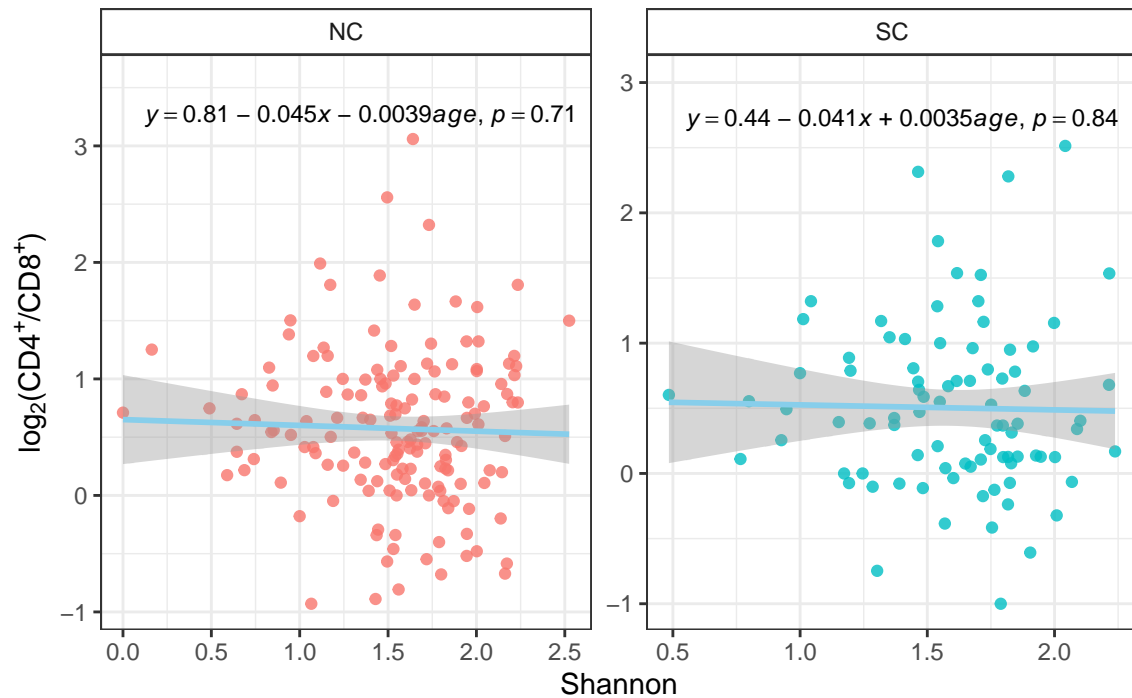**B**

Visit 2: NC vs. SC

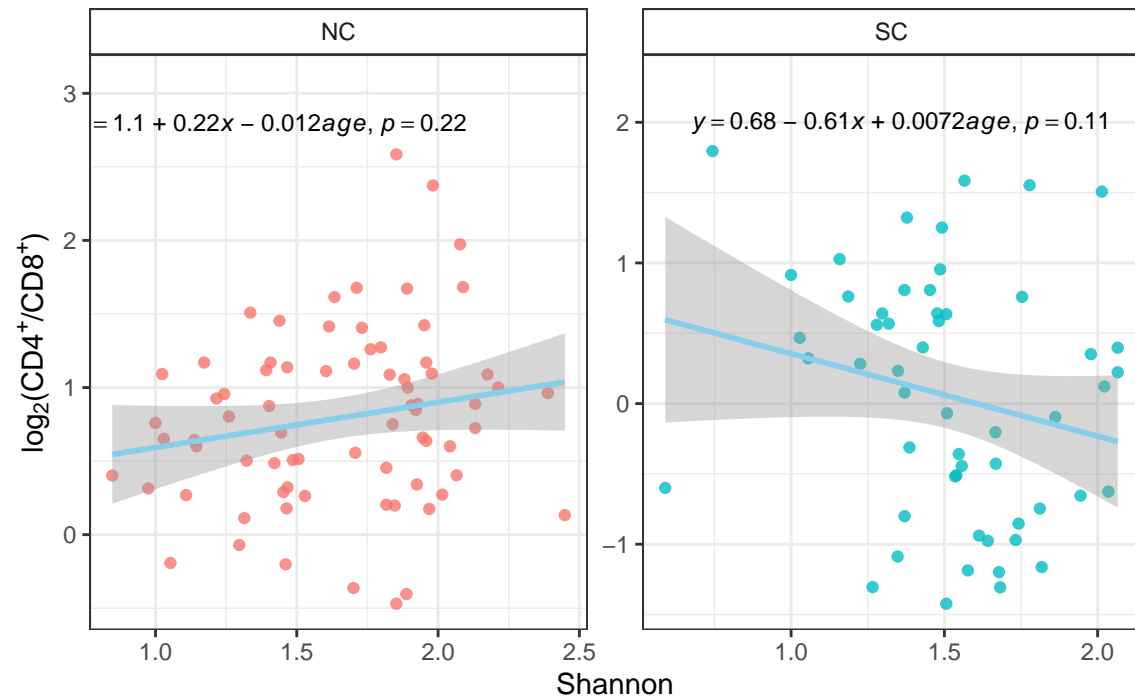

Supplement: Supplementary file 2 — Additional file 1. Supplementary figure S1. Sequence reads of bacterial 16S rRNA V4 gene of SC and NC stool samples. Supplementary figure S2. Correlation analysis of fecal microbiome Shannon diversity index and log2 ratio of peripheral blood CD4+/CD8+ at visit 1(A) or visit 2(B) of SC and NC. Supplementary figure S3. Correlation analysis of fecal microbiome Shannon diversity index and peripheral blood HIV loads at visit 2 of SC. Supplementary figure S4. The fecal microbiome compositions at phylum level of SC and NC at visit 1 and visit 2 obtained with 16S rRNA gene sequencing. Supplementary figure S5. Waterfall plot of log fold change (natural log) of absolute abundances for differentially abundant genera. A: SC vs. NC at visit 1; B. SC vs. NC at visit 2; C. visit 2 vs. visit 1 among SC; D. Time to develop AIDS < 5 years/5-10 years vs. > 10 years among SCs at visit 1; E. Time to develop AIDS < 5 years/5-10 years vs. > 10 years among SCs at visit 2. Data are represented by log fold change (shown as column) ±SE (shown as error bars) derived from the ANCOM-BC model. All effect sizes with p < 0.05 are indicated, *significant at 5% level of significance; **significant at 1% level of significance; ***significant at 0.1% level of significance. Taxa in blue were also significant after multiple testing correction was applied at FDR < 0.05. Exact p-values can be found in Supplementary Table 2. Supplementary figure S6. Correlation analysis of fecal SCFAs and log10 peripheral blood HIV loads at visit 2 of SC. Supplementary figure S7. Correlation of peripheral blood inflammatory cytokines and log2 ratio of CD4+/CD8+ at visit 1 (A) and visit 2 (B) of NC. Supplementary figure S8. Correlation of fecal microbiome alpha diversity (Shannon diversity index) at visit 1 and log2 ratio of peripheral blood CD4+/CD8+ at visit 2 of SC and NC. [file 40168_2021_1168_MOESM2_ESM.zip › fig_s2_ESM.pdf]

## SC at Visit 2

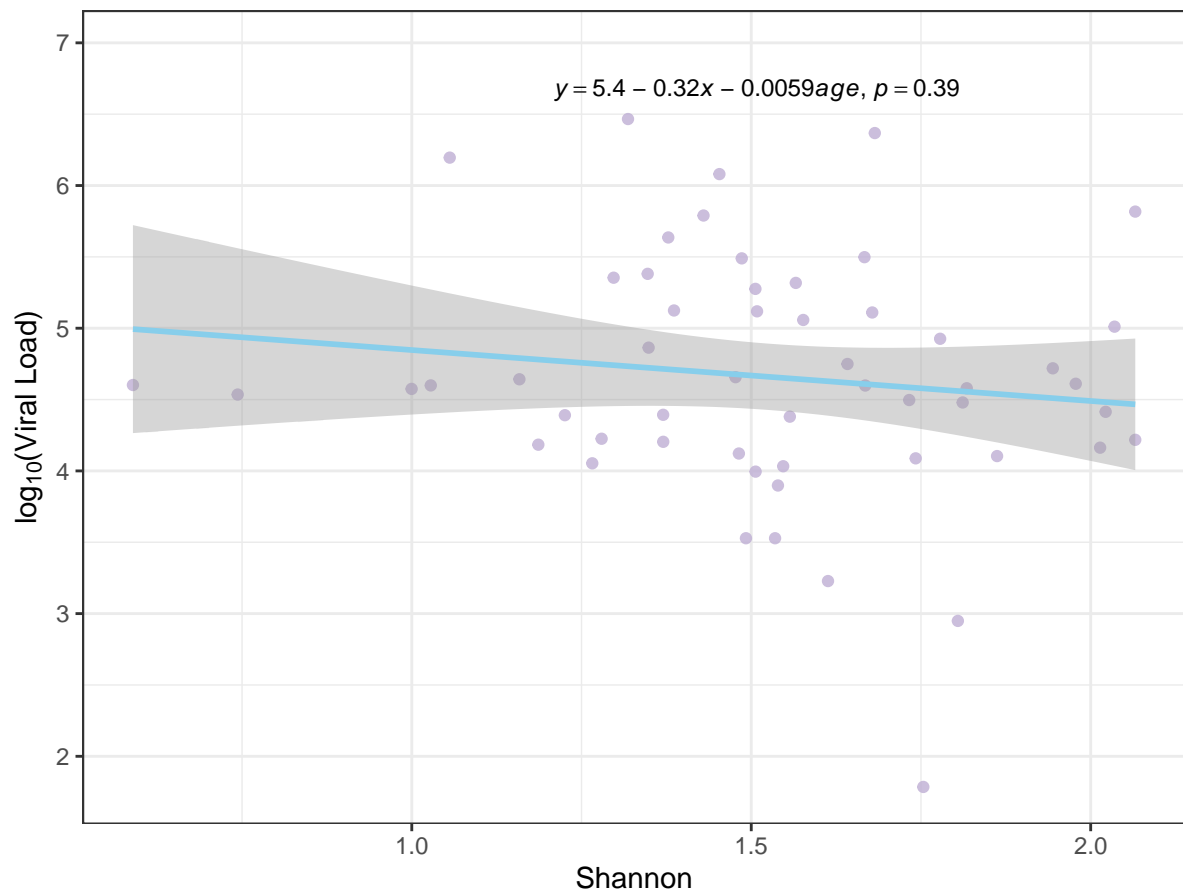

Supplement: Supplementary file 2 — Additional file 1. Supplementary figure S1. Sequence reads of bacterial 16S rRNA V4 gene of SC and NC stool samples. Supplementary figure S2. Correlation analysis of fecal microbiome Shannon diversity index and log2 ratio of peripheral blood CD4+/CD8+ at visit 1(A) or visit 2(B) of SC and NC. Supplementary figure S3. Correlation analysis of fecal microbiome Shannon diversity index and peripheral blood HIV loads at visit 2 of SC. Supplementary figure S4. The fecal microbiome compositions at phylum level of SC and NC at visit 1 and visit 2 obtained with 16S rRNA gene sequencing. Supplementary figure S5. Waterfall plot of log fold change (natural log) of absolute abundances for differentially abundant genera. A: SC vs. NC at visit 1; B. SC vs. NC at visit 2; C. visit 2 vs. visit 1 among SC; D. Time to develop AIDS < 5 years/5-10 years vs. > 10 years among SCs at visit 1; E. Time to develop AIDS < 5 years/5-10 years vs. > 10 years among SCs at visit 2. Data are represented by log fold change (shown as column) ±SE (shown as error bars) derived from the ANCOM-BC model. All effect sizes with p < 0.05 are indicated, *significant at 5% level of significance; **significant at 1% level of significance; ***significant at 0.1% level of significance. Taxa in blue were also significant after multiple testing correction was applied at FDR < 0.05. Exact p-values can be found in Supplementary Table 2. Supplementary figure S6. Correlation analysis of fecal SCFAs and log10 peripheral blood HIV loads at visit 2 of SC. Supplementary figure S7. Correlation of peripheral blood inflammatory cytokines and log2 ratio of CD4+/CD8+ at visit 1 (A) and visit 2 (B) of NC. Supplementary figure S8. Correlation of fecal microbiome alpha diversity (Shannon diversity index) at visit 1 and log2 ratio of peripheral blood CD4+/CD8+ at visit 2 of SC and NC. [file 40168_2021_1168_MOESM2_ESM.zip › fig_s3_ESM.pdf]

# Phylum Composition

NC: Visit 1

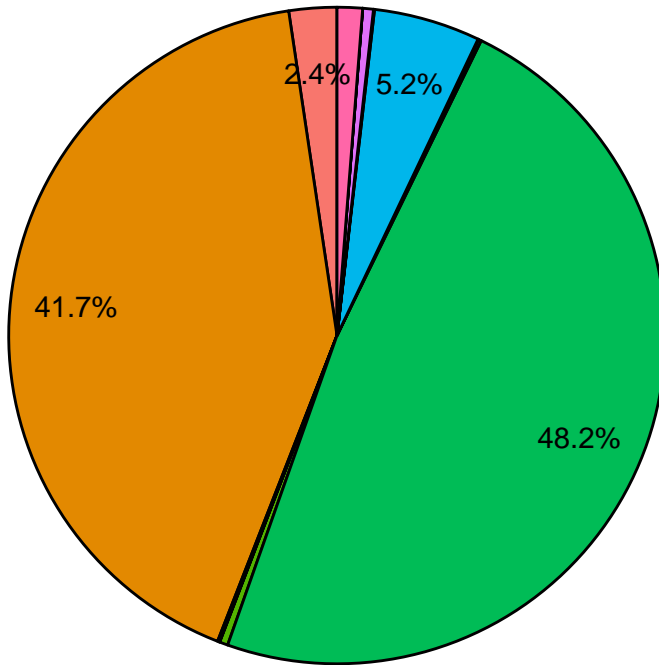

NC: Visit 2

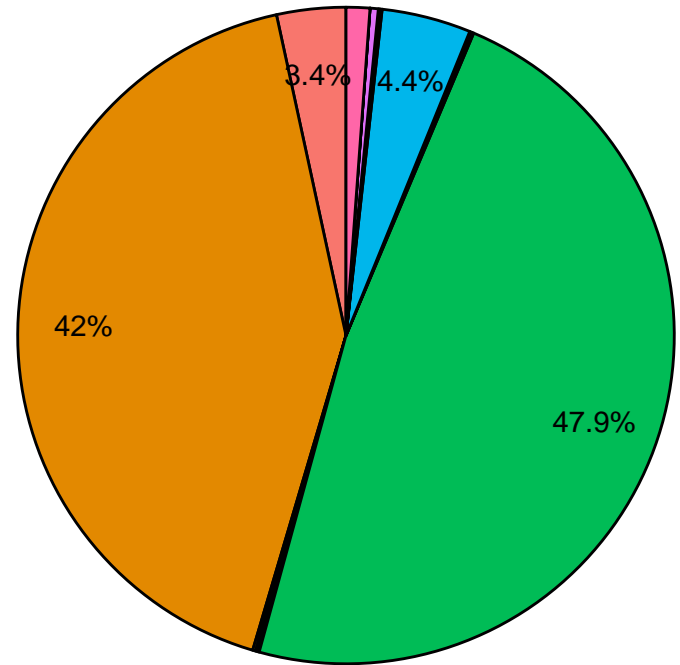

SC: Visit 1

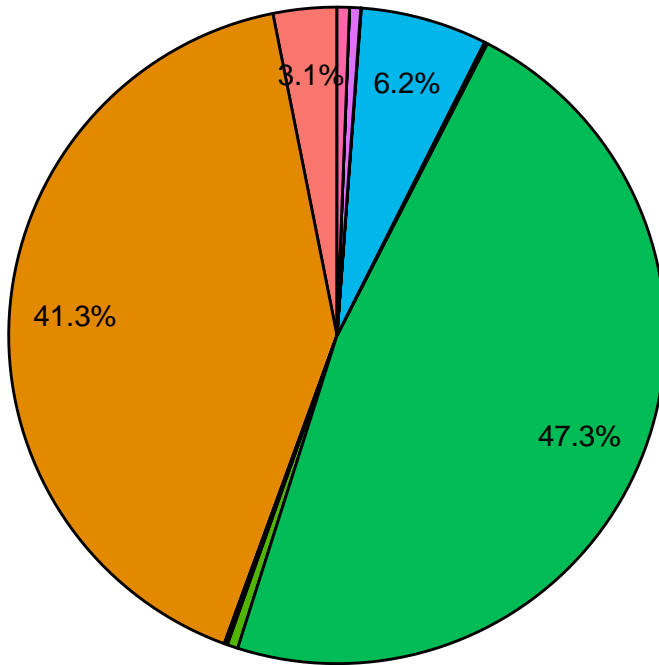

SC: Visit 2

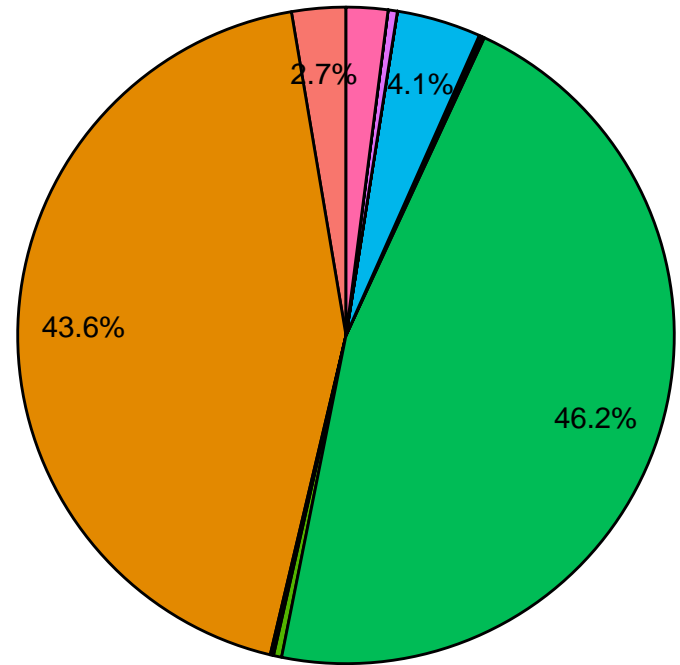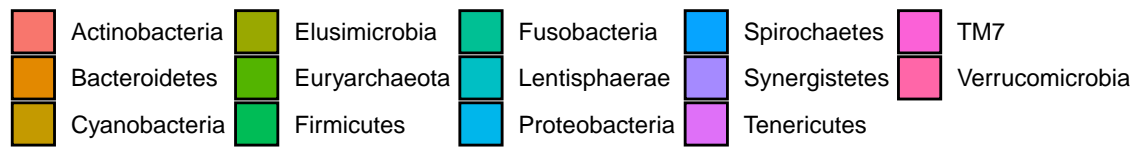

Supplement: Supplementary file 2 — Additional file 1. Supplementary figure S1. Sequence reads of bacterial 16S rRNA V4 gene of SC and NC stool samples. Supplementary figure S2. Correlation analysis of fecal microbiome Shannon diversity index and log2 ratio of peripheral blood CD4+/CD8+ at visit 1(A) or visit 2(B) of SC and NC. Supplementary figure S3. Correlation analysis of fecal microbiome Shannon diversity index and peripheral blood HIV loads at visit 2 of SC. Supplementary figure S4. The fecal microbiome compositions at phylum level of SC and NC at visit 1 and visit 2 obtained with 16S rRNA gene sequencing. Supplementary figure S5. Waterfall plot of log fold change (natural log) of absolute abundances for differentially abundant genera. A: SC vs. NC at visit 1; B. SC vs. NC at visit 2; C. visit 2 vs. visit 1 among SC; D. Time to develop AIDS < 5 years/5-10 years vs. > 10 years among SCs at visit 1; E. Time to develop AIDS < 5 years/5-10 years vs. > 10 years among SCs at visit 2. Data are represented by log fold change (shown as column) ±SE (shown as error bars) derived from the ANCOM-BC model. All effect sizes with p < 0.05 are indicated, *significant at 5% level of significance; **significant at 1% level of significance; ***significant at 0.1% level of significance. Taxa in blue were also significant after multiple testing correction was applied at FDR < 0.05. Exact p-values can be found in Supplementary Table 2. Supplementary figure S6. Correlation analysis of fecal SCFAs and log10 peripheral blood HIV loads at visit 2 of SC. Supplementary figure S7. Correlation of peripheral blood inflammatory cytokines and log2 ratio of CD4+/CD8+ at visit 1 (A) and visit 2 (B) of NC. Supplementary figure S8. Correlation of fecal microbiome alpha diversity (Shannon diversity index) at visit 1 and log2 ratio of peripheral blood CD4+/CD8+ at visit 2 of SC and NC. [file 40168_2021_1168_MOESM2_ESM.zip › fig_s4_ESM.pdf]

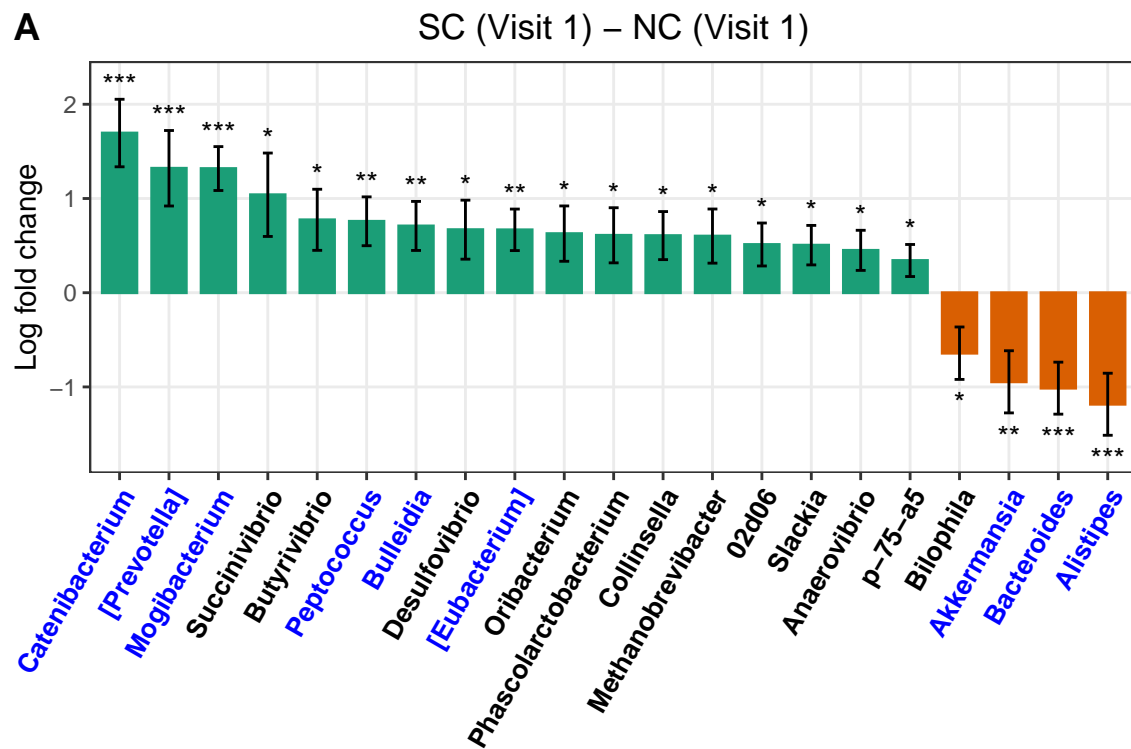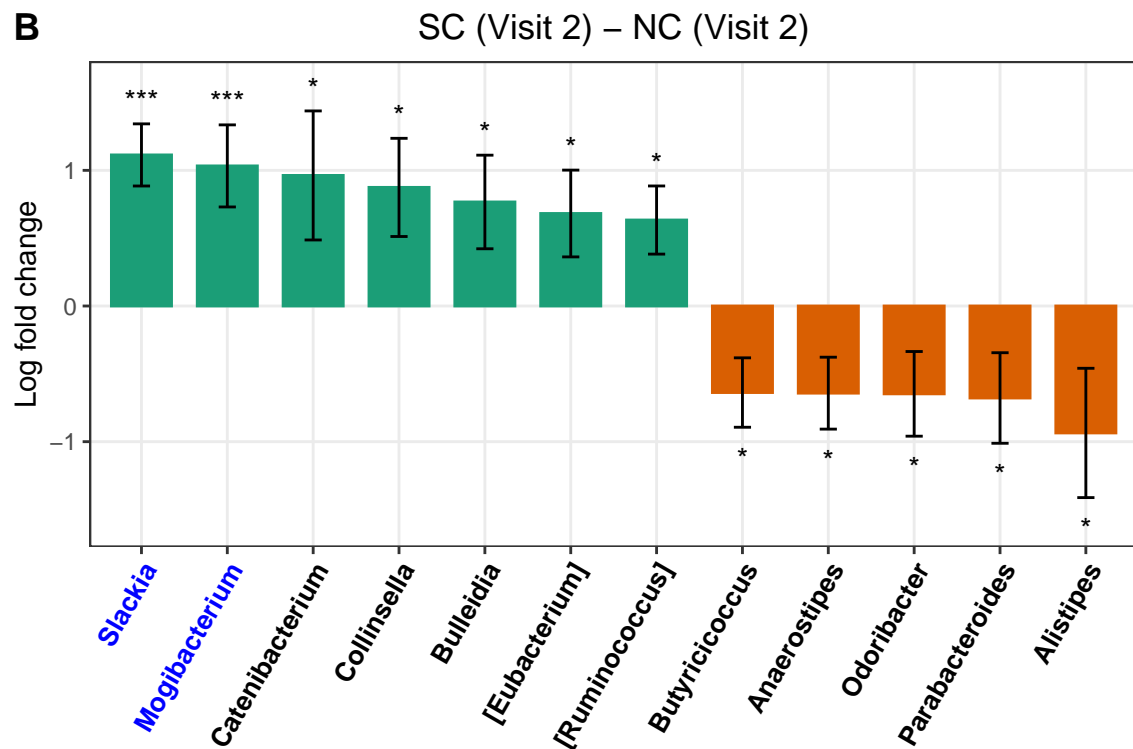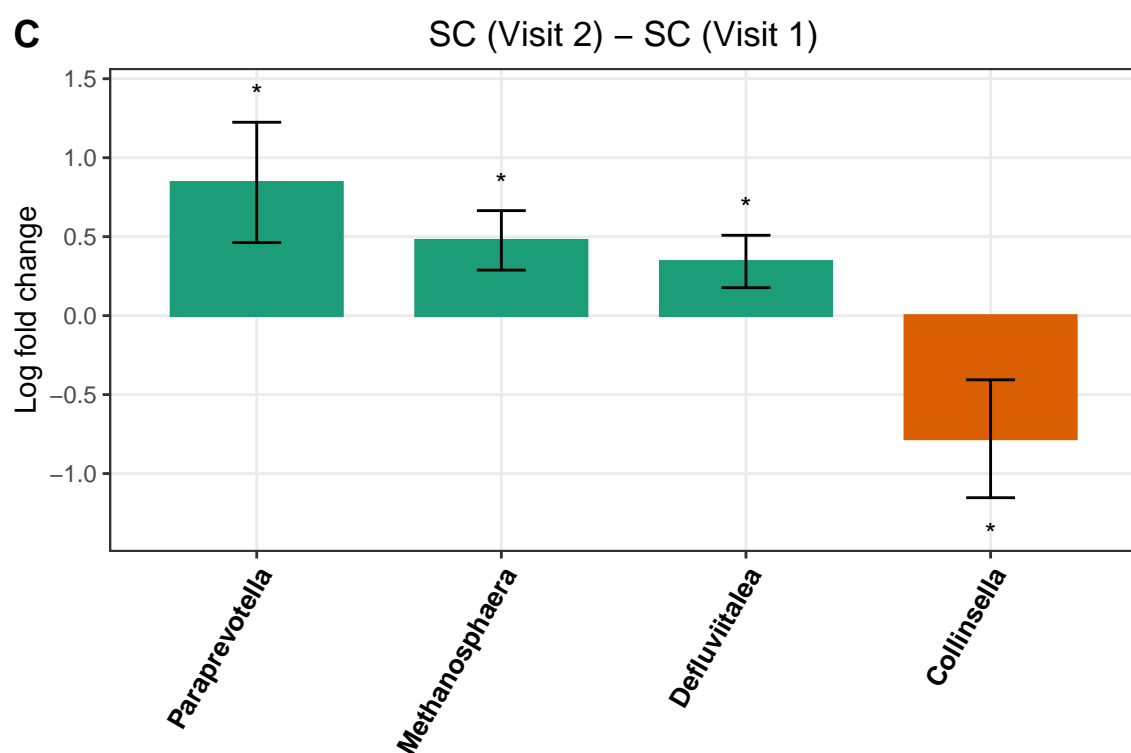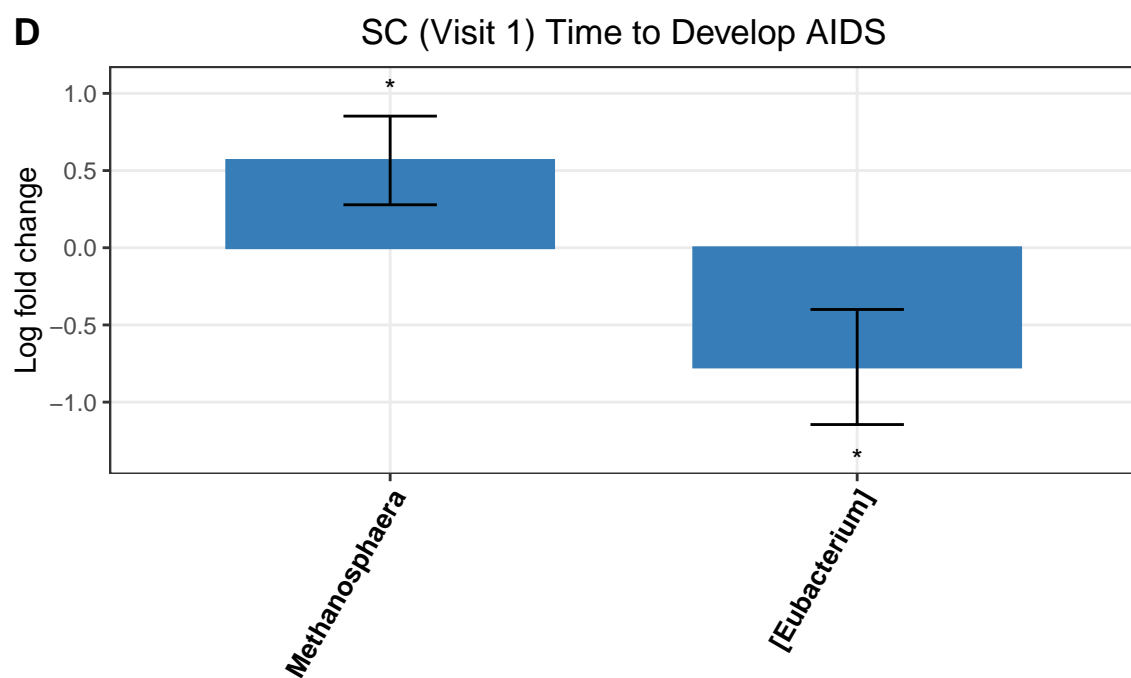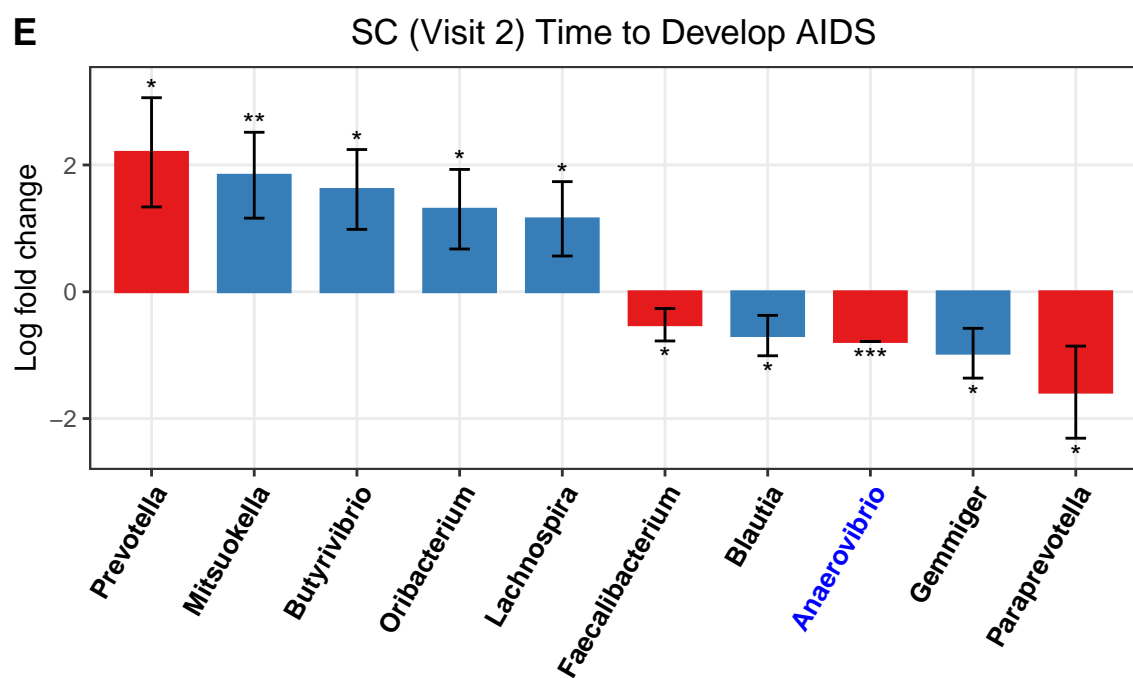

Legend: ■ < 5 years vs. > 10 years ■ 5 – 10 years vs. > 10 years

Supplement: Supplementary file 2 — Additional file 1. Supplementary figure S1. Sequence reads of bacterial 16S rRNA V4 gene of SC and NC stool samples. Supplementary figure S2. Correlation analysis of fecal microbiome Shannon diversity index and log2 ratio of peripheral blood CD4+/CD8+ at visit 1(A) or visit 2(B) of SC and NC. Supplementary figure S3. Correlation analysis of fecal microbiome Shannon diversity index and peripheral blood HIV loads at visit 2 of SC. Supplementary figure S4. The fecal microbiome compositions at phylum level of SC and NC at visit 1 and visit 2 obtained with 16S rRNA gene sequencing. Supplementary figure S5. Waterfall plot of log fold change (natural log) of absolute abundances for differentially abundant genera. A: SC vs. NC at visit 1; B. SC vs. NC at visit 2; C. visit 2 vs. visit 1 among SC; D. Time to develop AIDS < 5 years/5-10 years vs. > 10 years among SCs at visit 1; E. Time to develop AIDS < 5 years/5-10 years vs. > 10 years among SCs at visit 2. Data are represented by log fold change (shown as column) ±SE (shown as error bars) derived from the ANCOM-BC model. All effect sizes with p < 0.05 are indicated, *significant at 5% level of significance; **significant at 1% level of significance; ***significant at 0.1% level of significance. Taxa in blue were also significant after multiple testing correction was applied at FDR < 0.05. Exact p-values can be found in Supplementary Table 2. Supplementary figure S6. Correlation analysis of fecal SCFAs and log10 peripheral blood HIV loads at visit 2 of SC. Supplementary figure S7. Correlation of peripheral blood inflammatory cytokines and log2 ratio of CD4+/CD8+ at visit 1 (A) and visit 2 (B) of NC. Supplementary figure S8. Correlation of fecal microbiome alpha diversity (Shannon diversity index) at visit 1 and log2 ratio of peripheral blood CD4+/CD8+ at visit 2 of SC and NC. [file 40168_2021_1168_MOESM2_ESM.zip › fig_s5_ESM.pdf]

# SC: Visit 2

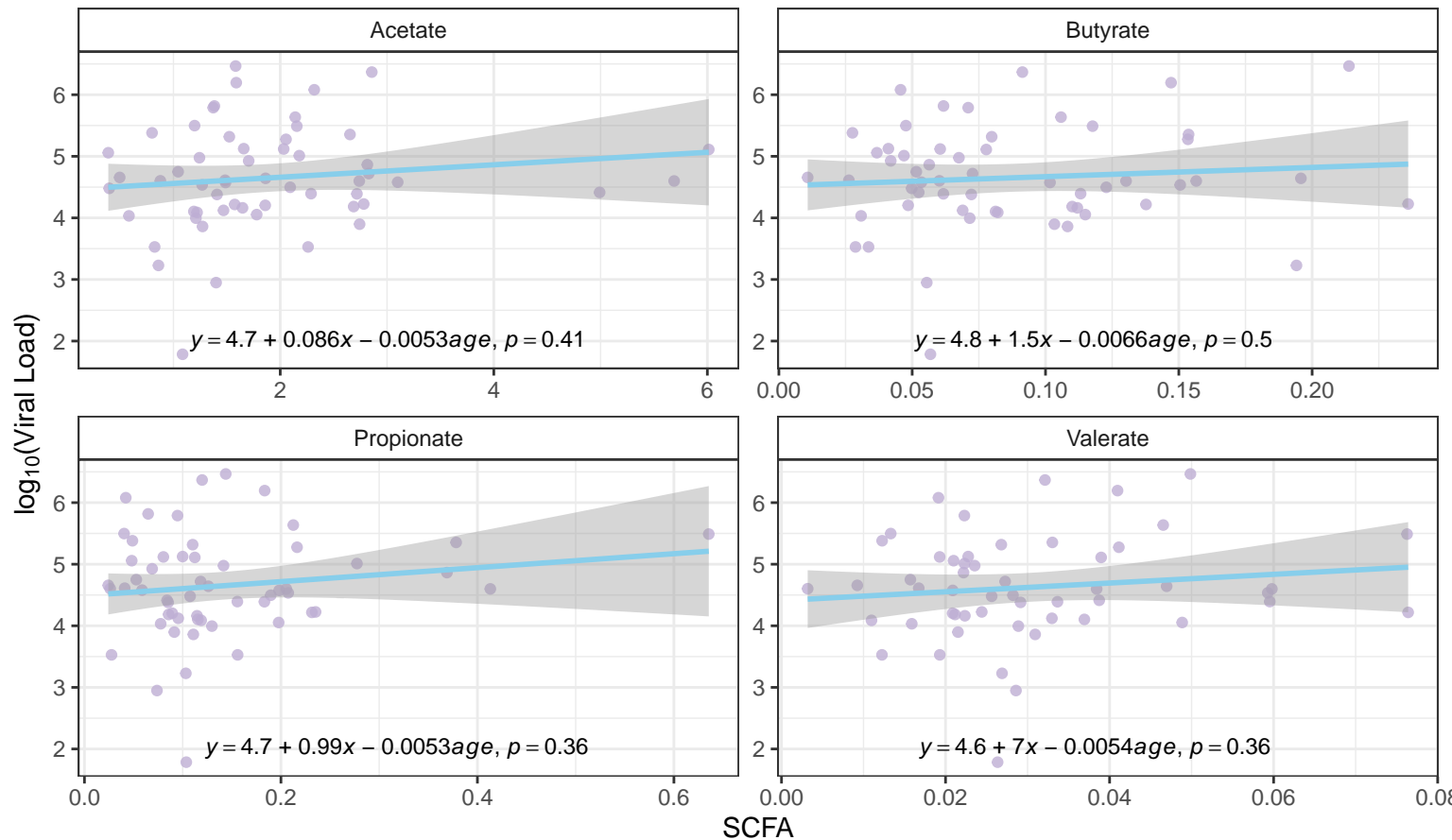

Supplement: Supplementary file 2 — Additional file 1. Supplementary figure S1. Sequence reads of bacterial 16S rRNA V4 gene of SC and NC stool samples. Supplementary figure S2. Correlation analysis of fecal microbiome Shannon diversity index and log2 ratio of peripheral blood CD4+/CD8+ at visit 1(A) or visit 2(B) of SC and NC. Supplementary figure S3. Correlation analysis of fecal microbiome Shannon diversity index and peripheral blood HIV loads at visit 2 of SC. Supplementary figure S4. The fecal microbiome compositions at phylum level of SC and NC at visit 1 and visit 2 obtained with 16S rRNA gene sequencing. Supplementary figure S5. Waterfall plot of log fold change (natural log) of absolute abundances for differentially abundant genera. A: SC vs. NC at visit 1; B. SC vs. NC at visit 2; C. visit 2 vs. visit 1 among SC; D. Time to develop AIDS < 5 years/5-10 years vs. > 10 years among SCs at visit 1; E. Time to develop AIDS < 5 years/5-10 years vs. > 10 years among SCs at visit 2. Data are represented by log fold change (shown as column) ±SE (shown as error bars) derived from the ANCOM-BC model. All effect sizes with p < 0.05 are indicated, *significant at 5% level of significance; **significant at 1% level of significance; ***significant at 0.1% level of significance. Taxa in blue were also significant after multiple testing correction was applied at FDR < 0.05. Exact p-values can be found in Supplementary Table 2. Supplementary figure S6. Correlation analysis of fecal SCFAs and log10 peripheral blood HIV loads at visit 2 of SC. Supplementary figure S7. Correlation of peripheral blood inflammatory cytokines and log2 ratio of CD4+/CD8+ at visit 1 (A) and visit 2 (B) of NC. Supplementary figure S8. Correlation of fecal microbiome alpha diversity (Shannon diversity index) at visit 1 and log2 ratio of peripheral blood CD4+/CD8+ at visit 2 of SC and NC. [file 40168_2021_1168_MOESM2_ESM.zip › fig_s6_ESM.pdf]

**A**

NC: Visit 1

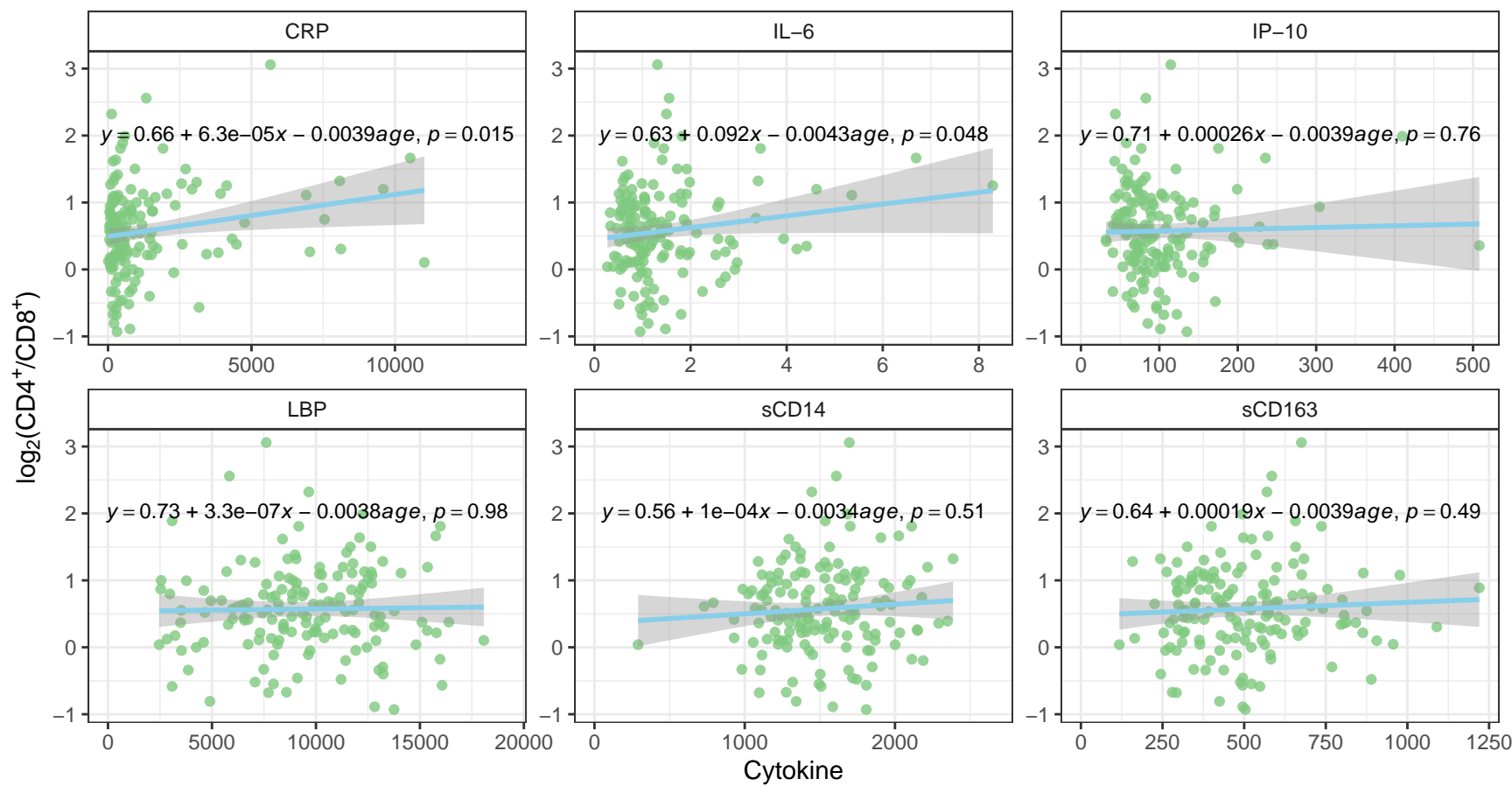**B**

NC: Visit 2

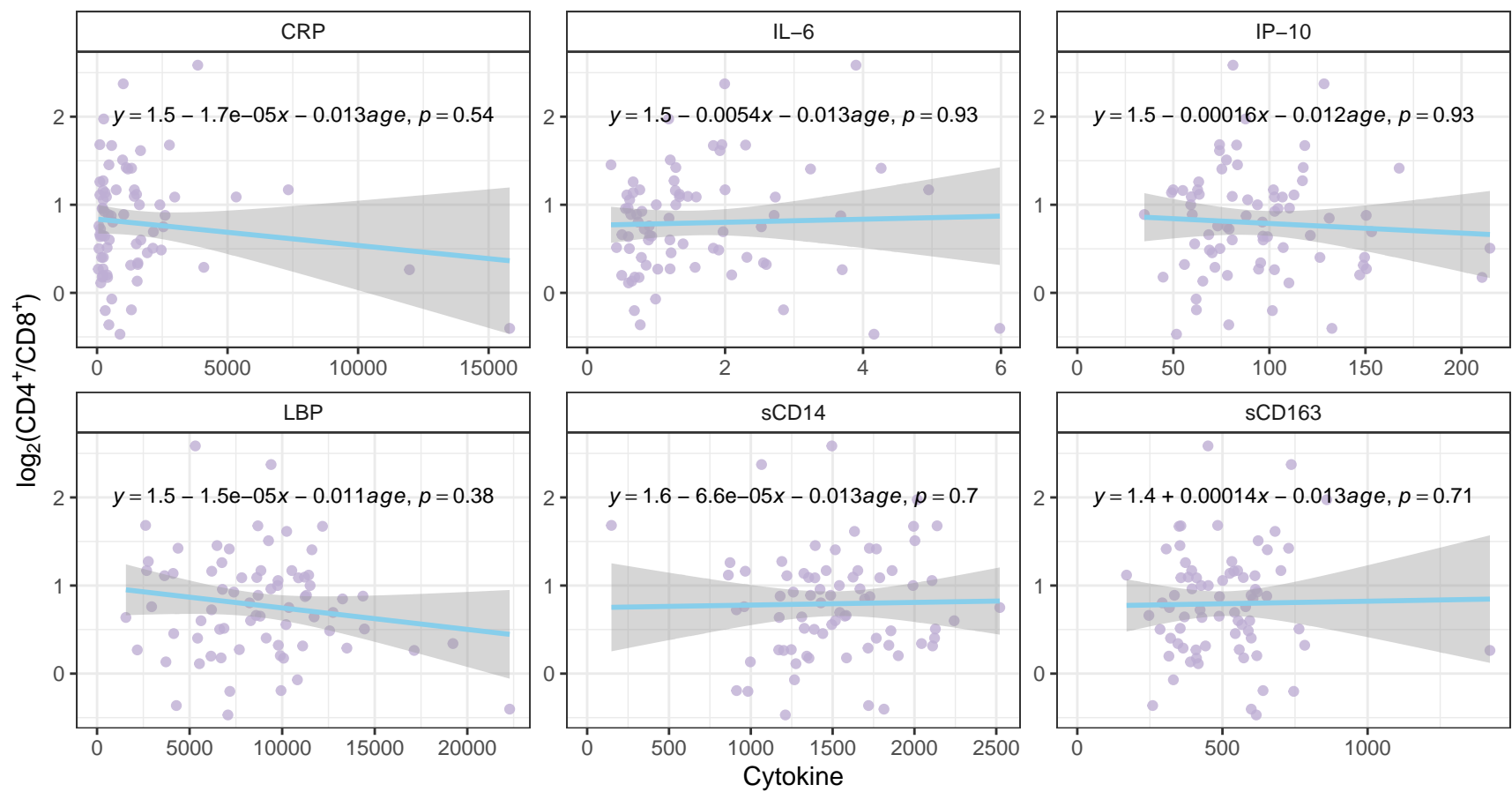

Supplement: Supplementary file 2 — Additional file 1. Supplementary figure S1. Sequence reads of bacterial 16S rRNA V4 gene of SC and NC stool samples. Supplementary figure S2. Correlation analysis of fecal microbiome Shannon diversity index and log2 ratio of peripheral blood CD4+/CD8+ at visit 1(A) or visit 2(B) of SC and NC. Supplementary figure S3. Correlation analysis of fecal microbiome Shannon diversity index and peripheral blood HIV loads at visit 2 of SC. Supplementary figure S4. The fecal microbiome compositions at phylum level of SC and NC at visit 1 and visit 2 obtained with 16S rRNA gene sequencing. Supplementary figure S5. Waterfall plot of log fold change (natural log) of absolute abundances for differentially abundant genera. A: SC vs. NC at visit 1; B. SC vs. NC at visit 2; C. visit 2 vs. visit 1 among SC; D. Time to develop AIDS < 5 years/5-10 years vs. > 10 years among SCs at visit 1; E. Time to develop AIDS < 5 years/5-10 years vs. > 10 years among SCs at visit 2. Data are represented by log fold change (shown as column) ±SE (shown as error bars) derived from the ANCOM-BC model. All effect sizes with p < 0.05 are indicated, *significant at 5% level of significance; **significant at 1% level of significance; ***significant at 0.1% level of significance. Taxa in blue were also significant after multiple testing correction was applied at FDR < 0.05. Exact p-values can be found in Supplementary Table 2. Supplementary figure S6. Correlation analysis of fecal SCFAs and log10 peripheral blood HIV loads at visit 2 of SC. Supplementary figure S7. Correlation of peripheral blood inflammatory cytokines and log2 ratio of CD4+/CD8+ at visit 1 (A) and visit 2 (B) of NC. Supplementary figure S8. Correlation of fecal microbiome alpha diversity (Shannon diversity index) at visit 1 and log2 ratio of peripheral blood CD4+/CD8+ at visit 2 of SC and NC. [file 40168_2021_1168_MOESM2_ESM.zip › fig_s7_ESM.pdf]

$\log_2(\text{CD4}^+/\text{CD8}^+)$  at Visit 2 vs. Shannon Diversity Index at Visit 1

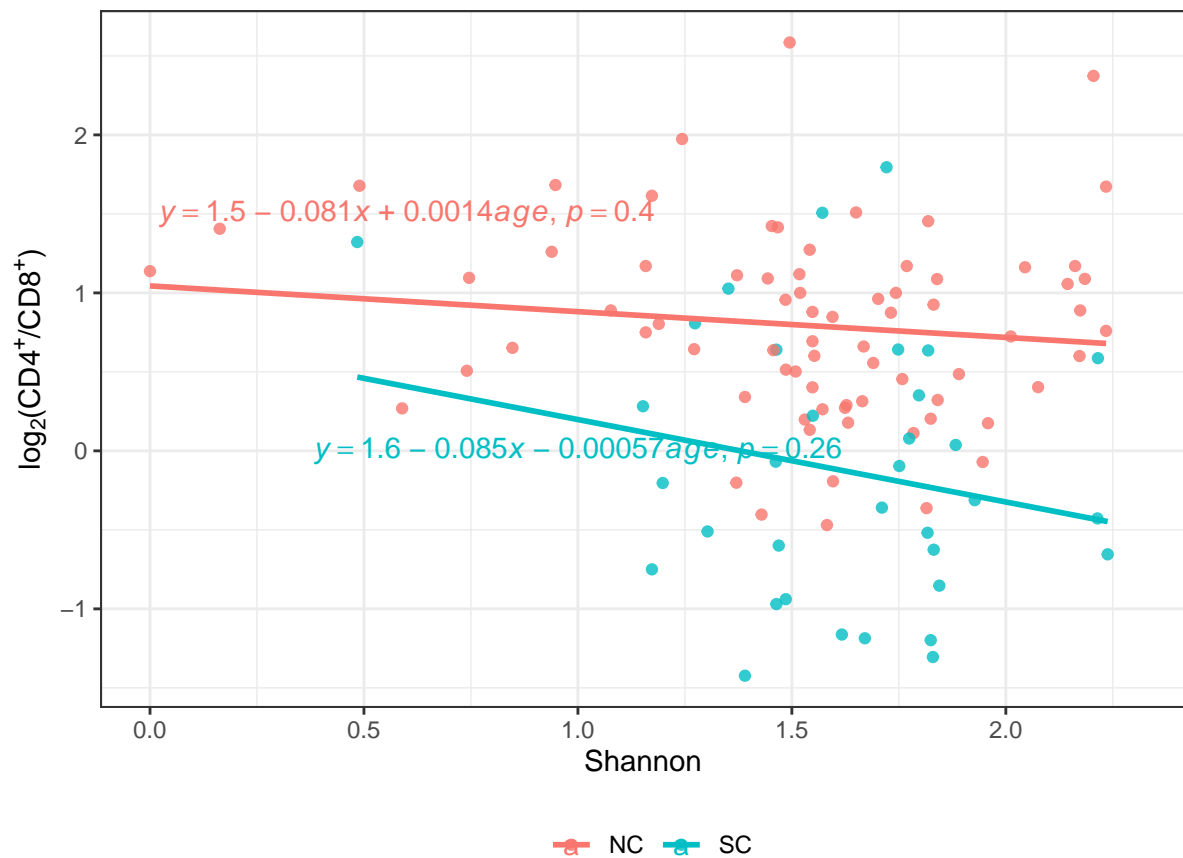

Supplement: Supplementary file 2 — Additional file 1. Supplementary figure S1. Sequence reads of bacterial 16S rRNA V4 gene of SC and NC stool samples. Supplementary figure S2. Correlation analysis of fecal microbiome Shannon diversity index and log2 ratio of peripheral blood CD4+/CD8+ at visit 1(A) or visit 2(B) of SC and NC. Supplementary figure S3. Correlation analysis of fecal microbiome Shannon diversity index and peripheral blood HIV loads at visit 2 of SC. Supplementary figure S4. The fecal microbiome compositions at phylum level of SC and NC at visit 1 and visit 2 obtained with 16S rRNA gene sequencing. Supplementary figure S5. Waterfall plot of log fold change (natural log) of absolute abundances for differentially abundant genera. A: SC vs. NC at visit 1; B. SC vs. NC at visit 2; C. visit 2 vs. visit 1 among SC; D. Time to develop AIDS < 5 years/5-10 years vs. > 10 years among SCs at visit 1; E. Time to develop AIDS < 5 years/5-10 years vs. > 10 years among SCs at visit 2. Data are represented by log fold change (shown as column) ±SE (shown as error bars) derived from the ANCOM-BC model. All effect sizes with p < 0.05 are indicated, *significant at 5% level of significance; **significant at 1% level of significance; ***significant at 0.1% level of significance. Taxa in blue were also significant after multiple testing correction was applied at FDR < 0.05. Exact p-values can be found in Supplementary Table 2. Supplementary figure S6. Correlation analysis of fecal SCFAs and log10 peripheral blood HIV loads at visit 2 of SC. Supplementary figure S7. Correlation of peripheral blood inflammatory cytokines and log2 ratio of CD4+/CD8+ at visit 1 (A) and visit 2 (B) of NC. Supplementary figure S8. Correlation of fecal microbiome alpha diversity (Shannon diversity index) at visit 1 and log2 ratio of peripheral blood CD4+/CD8+ at visit 2 of SC and NC. [file 40168_2021_1168_MOESM2_ESM.zip › fig_s8_ESM.pdf]
