# Supplementary material for: Signature changes in gut microbiome are associated with increased susceptibility to HIV-1 infection in MSM
Source: Microbiome. 2021 Dec 9;9:237. doi: 10.1186/s40168-021-01168-w (PMC8656045; doi:10.1186/s40168-021-01168-w)
Supplement: Supplementary file 3 — Additional file 2. Supplementary Table 1. The log fold change (LFC) of absolute abundances for differentially abundant families (A), (B), (C), (D), (E). *The taxon was declared to be significant since the absolute abundance in the reference group (Time to Develop AIDS > 10 Years) was zero. **NE: not evaluable. Supplementary Table 2. The log fold change (LFC) of absolute abundances for differentially abundant genera (A), (B), (C), (D), (E). *The taxon was declared to be significant since the absolute abundance in the group of interest (Time to Develop AIDS < 5 Years) was zero. **NE: not evaluable. Supplementary Table 3. The log fold change (LFC) of absolute abundances for differentially abundant families (A), (B), (C), (D), (E). *The taxon was declared to be significant since the absolute abundance in the group of interest (Time to Develop AIDS 5 - 10 Years) was zero. **NE: not evaluable. ***The taxon was declared to be significant since the absolute abundance in the reference group (Time to Develop AIDS > 10 Years) was zero. [file 40168_2021_1168_MOESM3_ESM.pptx]

## Slide 1
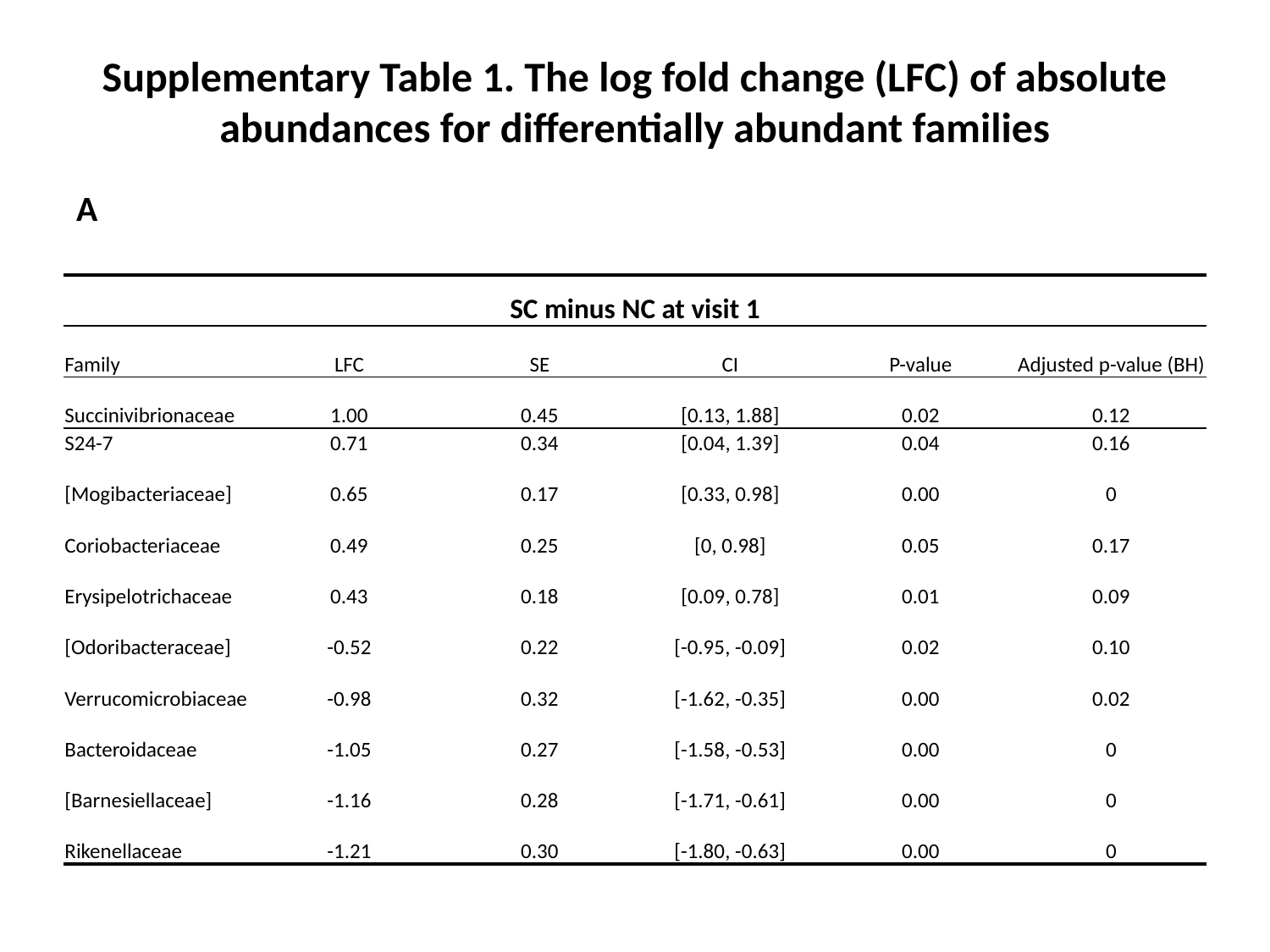

# Supplementary Table 1. The log fold change (LFC) of absolute abundances for differentially abundant families
A
| SC minus NC at visit 1 | | | | | |
| --- | --- | --- | --- | --- | --- |
| Family | LFC | SE | CI | P-value | Adjusted p-value (BH) |
| Succinivibrionaceae | 1.00 | 0.45 | [0.13, 1.88] | 0.02 | 0.12 |
| S24-7 | 0.71 | 0.34 | [0.04, 1.39] | 0.04 | 0.16 |
| [Mogibacteriaceae] | 0.65 | 0.17 | [0.33, 0.98] | 0.00 | 0 |
| Coriobacteriaceae | 0.49 | 0.25 | [0, 0.98] | 0.05 | 0.17 |
| Erysipelotrichaceae | 0.43 | 0.18 | [0.09, 0.78] | 0.01 | 0.09 |
| [Odoribacteraceae] | -0.52 | 0.22 | [-0.95, -0.09] | 0.02 | 0.10 |
| Verrucomicrobiaceae | -0.98 | 0.32 | [-1.62, -0.35] | 0.00 | 0.02 |
| Bacteroidaceae | -1.05 | 0.27 | [-1.58, -0.53] | 0.00 | 0 |
| [Barnesiellaceae] | -1.16 | 0.28 | [-1.71, -0.61] | 0.00 | 0 |
| Rikenellaceae | -1.21 | 0.30 | [-1.80, -0.63] | 0.00 | 0 |

## Slide 2
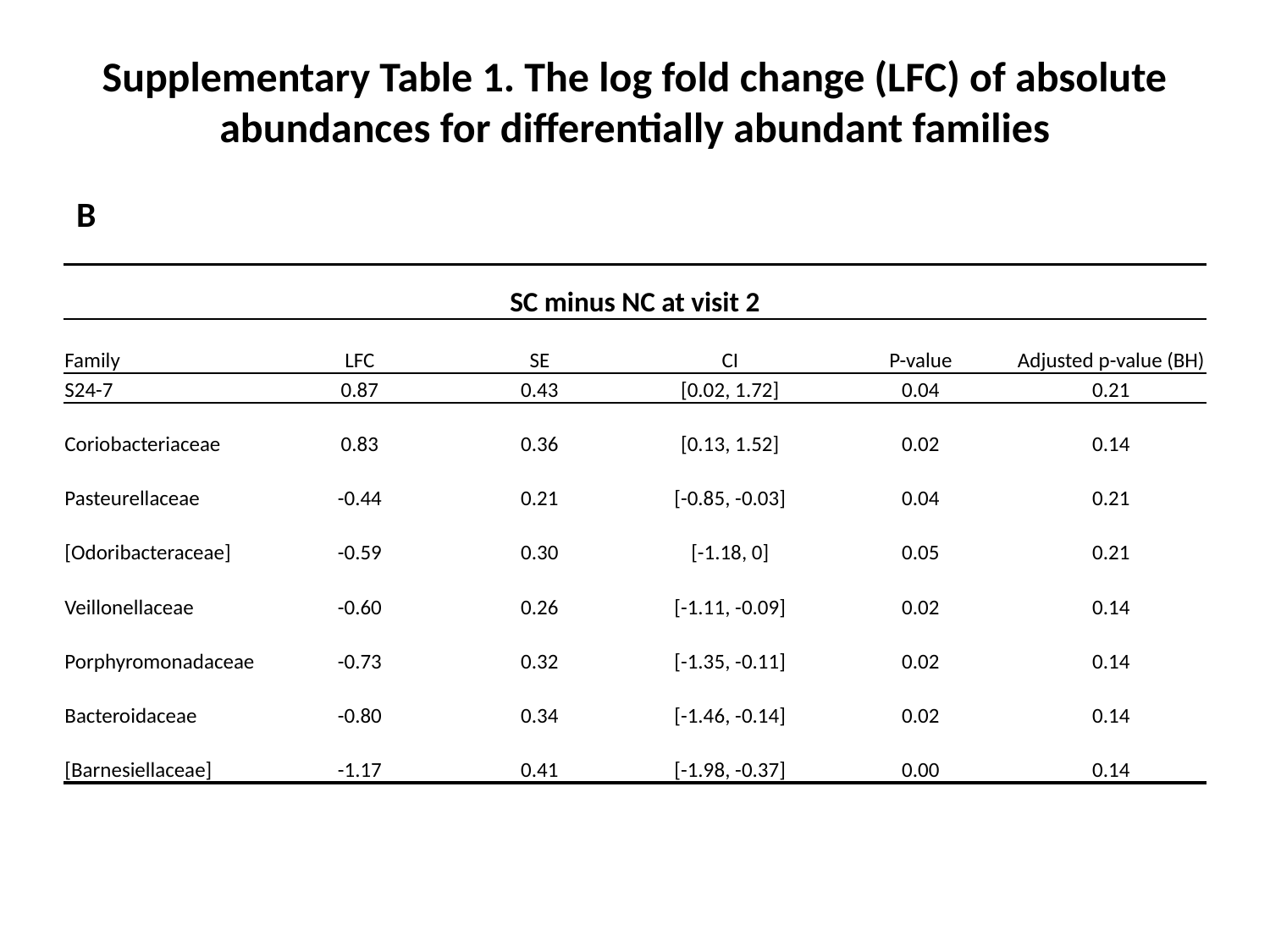

# Supplementary Table 1. The log fold change (LFC) of absolute abundances for differentially abundant families
B
| SC minus NC at visit 2 | | | | | |
| --- | --- | --- | --- | --- | --- |
| Family | LFC | SE | CI | P-value | Adjusted p-value (BH) |
| S24-7 | 0.87 | 0.43 | [0.02, 1.72] | 0.04 | 0.21 |
| Coriobacteriaceae | 0.83 | 0.36 | [0.13, 1.52] | 0.02 | 0.14 |
| Pasteurellaceae | -0.44 | 0.21 | [-0.85, -0.03] | 0.04 | 0.21 |
| [Odoribacteraceae] | -0.59 | 0.30 | [-1.18, 0] | 0.05 | 0.21 |
| Veillonellaceae | -0.60 | 0.26 | [-1.11, -0.09] | 0.02 | 0.14 |
| Porphyromonadaceae | -0.73 | 0.32 | [-1.35, -0.11] | 0.02 | 0.14 |
| Bacteroidaceae | -0.80 | 0.34 | [-1.46, -0.14] | 0.02 | 0.14 |
| [Barnesiellaceae] | -1.17 | 0.41 | [-1.98, -0.37] | 0.00 | 0.14 |

## Slide 3
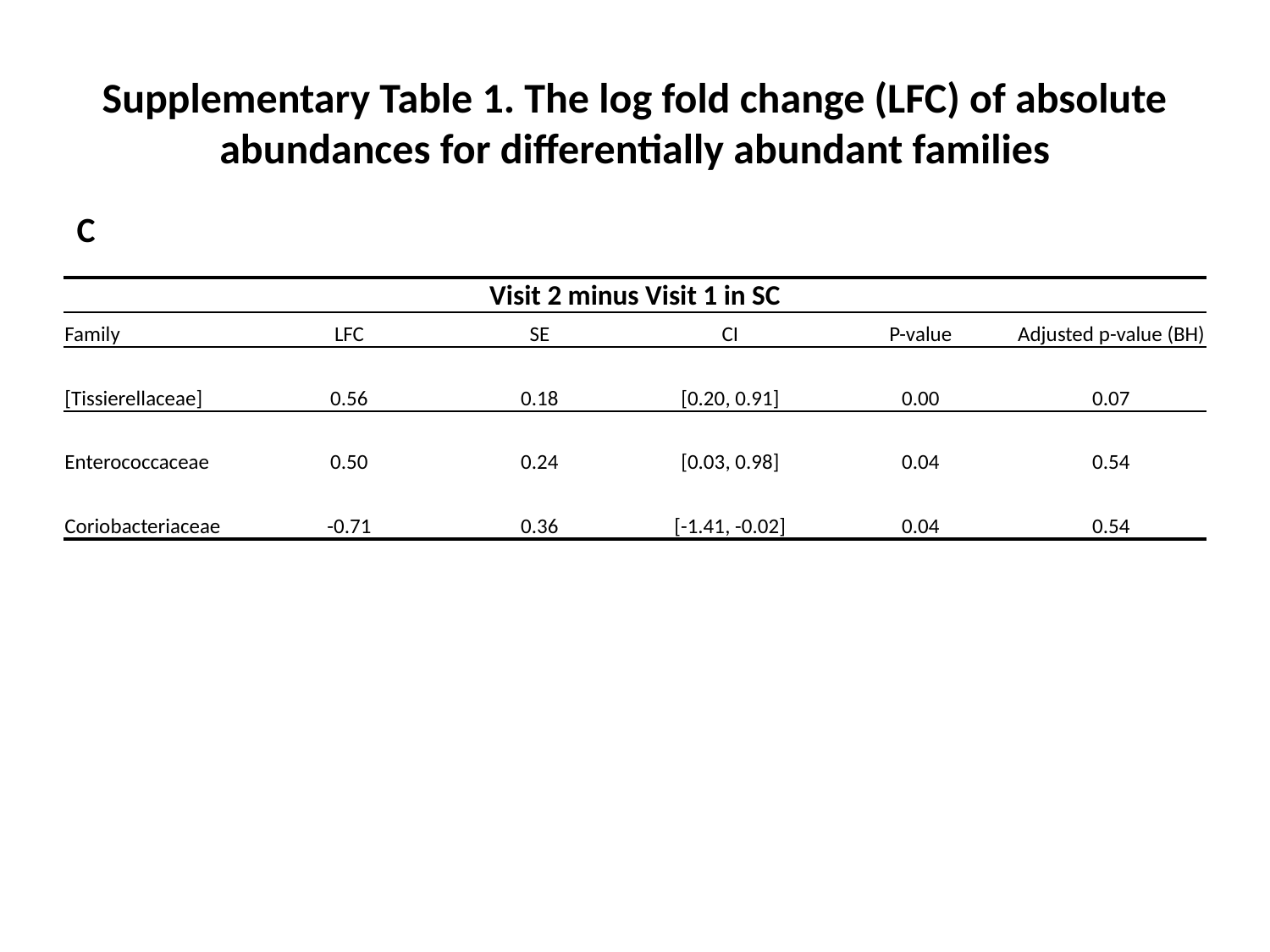

# Supplementary Table 1. The log fold change (LFC) of absolute abundances for differentially abundant families
C
| Visit 2 minus Visit 1 in SC | | | | | |
| --- | --- | --- | --- | --- | --- |
| Family | LFC | SE | CI | P-value | Adjusted p-value (BH) |
| [Tissierellaceae] | 0.56 | 0.18 | [0.20, 0.91] | 0.00 | 0.07 |
| Enterococcaceae | 0.50 | 0.24 | [0.03, 0.98] | 0.04 | 0.54 |
| Coriobacteriaceae | -0.71 | 0.36 | [-1.41, -0.02] | 0.04 | 0.54 |

## Slide 4
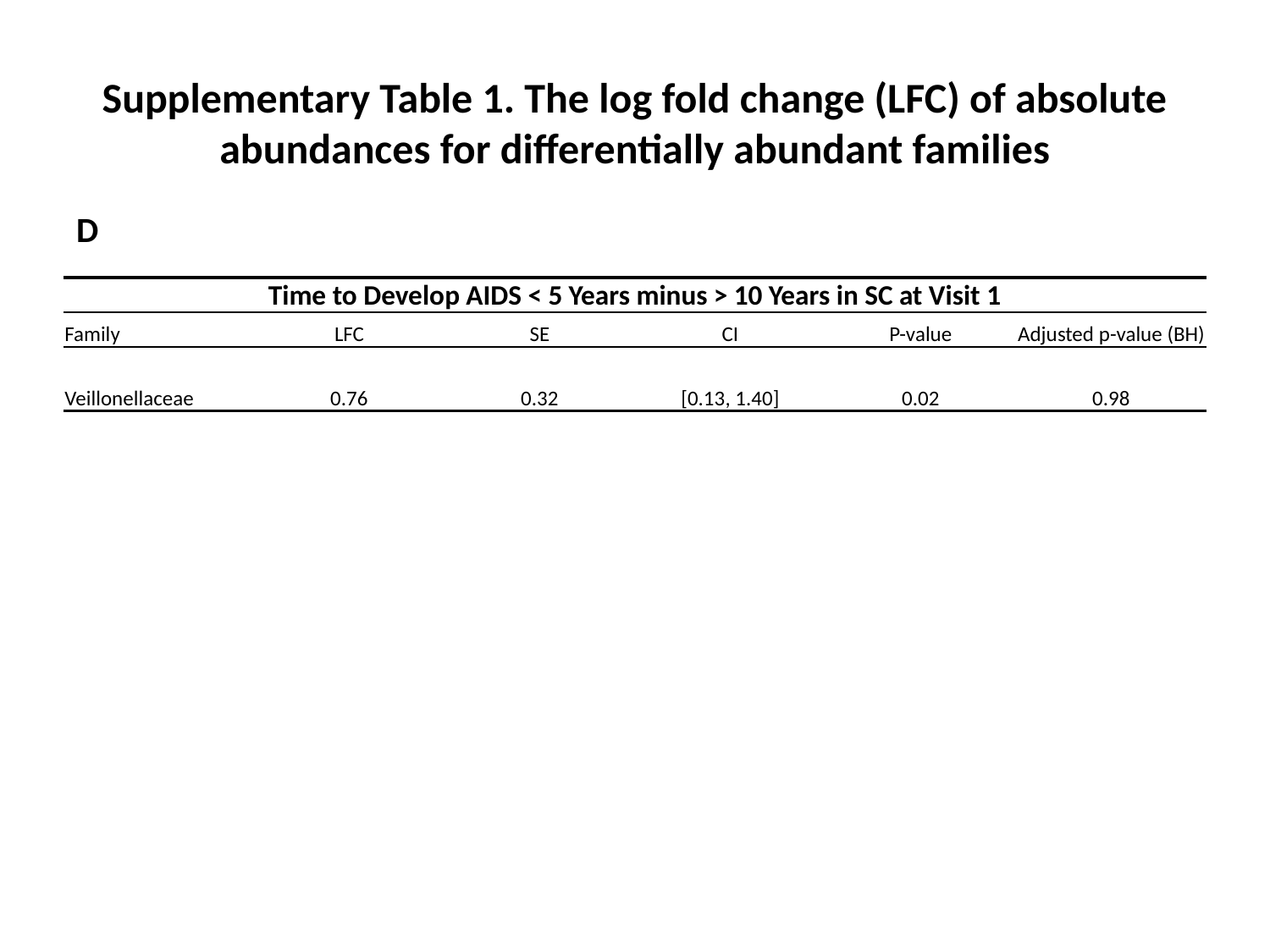

# Supplementary Table 1. The log fold change (LFC) of absolute abundances for differentially abundant families
D
| Time to Develop AIDS < 5 Years minus > 10 Years in SC at Visit 1 | | | | | |
| --- | --- | --- | --- | --- | --- |
| Family | LFC | SE | CI | P-value | Adjusted p-value (BH) |
| Veillonellaceae | 0.76 | 0.32 | [0.13, 1.40] | 0.02 | 0.98 |

## Slide 5
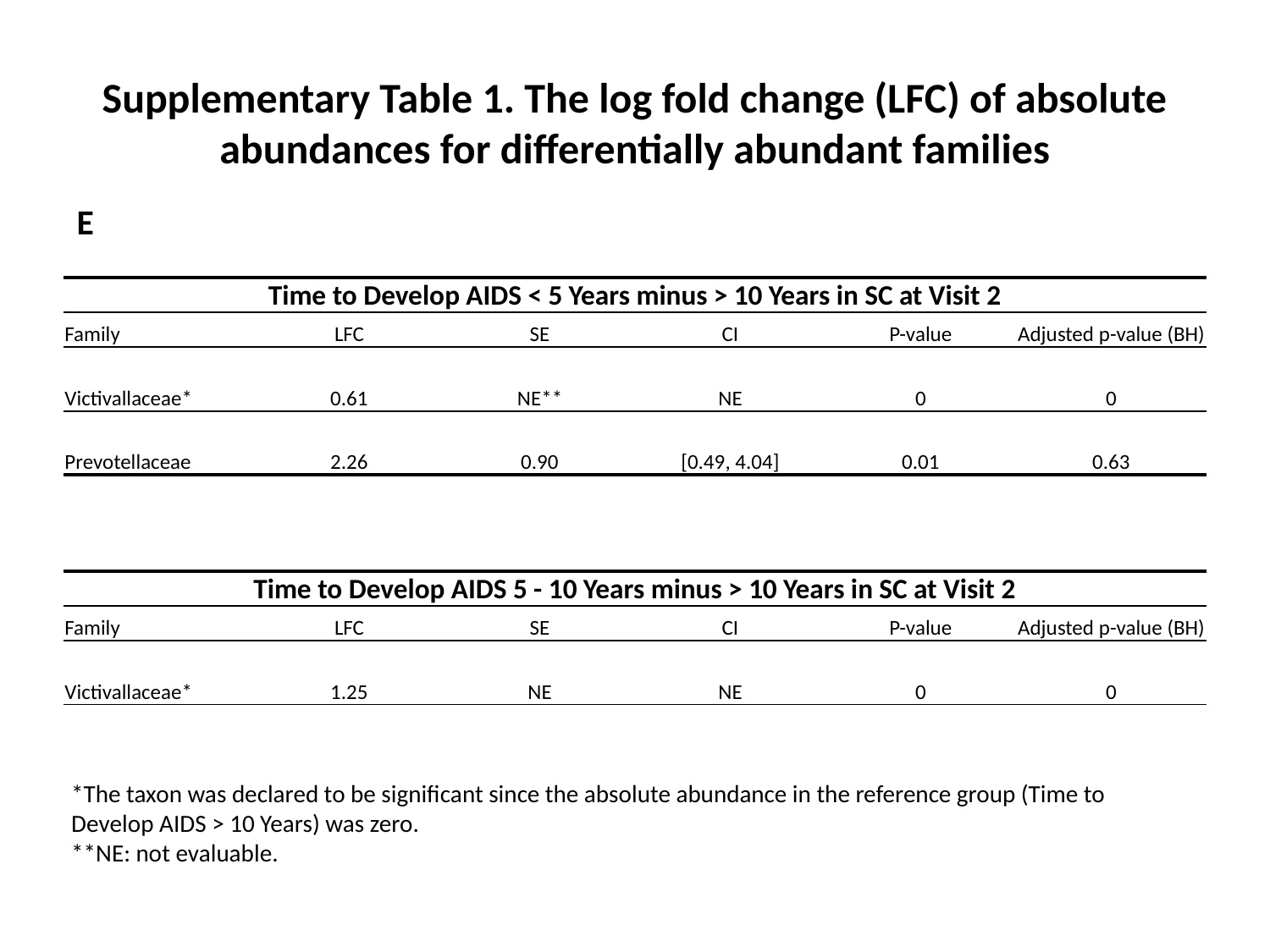

# Supplementary Table 1. The log fold change (LFC) of absolute abundances for differentially abundant families
E
| Time to Develop AIDS < 5 Years minus > 10 Years in SC at Visit 2 | | | | | |
| --- | --- | --- | --- | --- | --- |
| Family | LFC | SE | CI | P-value | Adjusted p-value (BH) |
| Victivallaceae\* | 0.61 | NE\*\* | NE | 0 | 0 |
| Prevotellaceae | 2.26 | 0.90 | [0.49, 4.04] | 0.01 | 0.63 |
| Time to Develop AIDS 5 - 10 Years minus > 10 Years in SC at Visit 2 | | | | | |
| --- | --- | --- | --- | --- | --- |
| Family | LFC | SE | CI | P-value | Adjusted p-value (BH) |
| Victivallaceae\* | 1.25 | NE | NE | 0 | 0 |
*The taxon was declared to be significant since the absolute abundance in the reference group (Time to Develop AIDS > 10 Years) was zero.
**NE: not evaluable.

## Slide 6
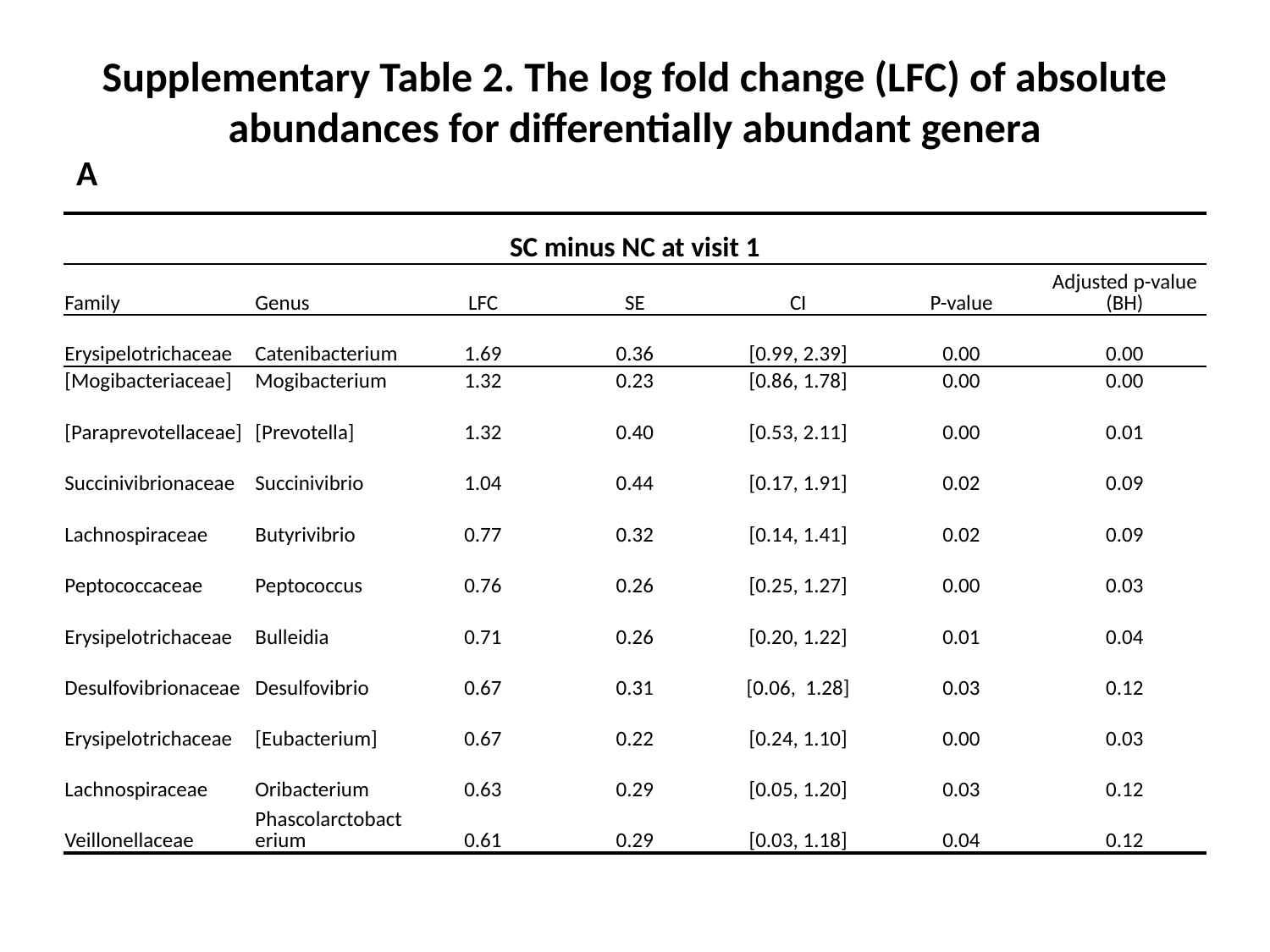

# Supplementary Table 2. The log fold change (LFC) of absolute abundances for differentially abundant genera
A
| SC minus NC at visit 1 | | | | | | |
| --- | --- | --- | --- | --- | --- | --- |
| Family | Genus | LFC | SE | CI | P-value | Adjusted p-value (BH) |
| Erysipelotrichaceae | Catenibacterium | 1.69 | 0.36 | [0.99, 2.39] | 0.00 | 0.00 |
| [Mogibacteriaceae] | Mogibacterium | 1.32 | 0.23 | [0.86, 1.78] | 0.00 | 0.00 |
| [Paraprevotellaceae] | [Prevotella] | 1.32 | 0.40 | [0.53, 2.11] | 0.00 | 0.01 |
| Succinivibrionaceae | Succinivibrio | 1.04 | 0.44 | [0.17, 1.91] | 0.02 | 0.09 |
| Lachnospiraceae | Butyrivibrio | 0.77 | 0.32 | [0.14, 1.41] | 0.02 | 0.09 |
| Peptococcaceae | Peptococcus | 0.76 | 0.26 | [0.25, 1.27] | 0.00 | 0.03 |
| Erysipelotrichaceae | Bulleidia | 0.71 | 0.26 | [0.20, 1.22] | 0.01 | 0.04 |
| Desulfovibrionaceae | Desulfovibrio | 0.67 | 0.31 | [0.06, 1.28] | 0.03 | 0.12 |
| Erysipelotrichaceae | [Eubacterium] | 0.67 | 0.22 | [0.24, 1.10] | 0.00 | 0.03 |
| Lachnospiraceae | Oribacterium | 0.63 | 0.29 | [0.05, 1.20] | 0.03 | 0.12 |
| Veillonellaceae | Phascolarctobacterium | 0.61 | 0.29 | [0.03, 1.18] | 0.04 | 0.12 |

## Slide 7
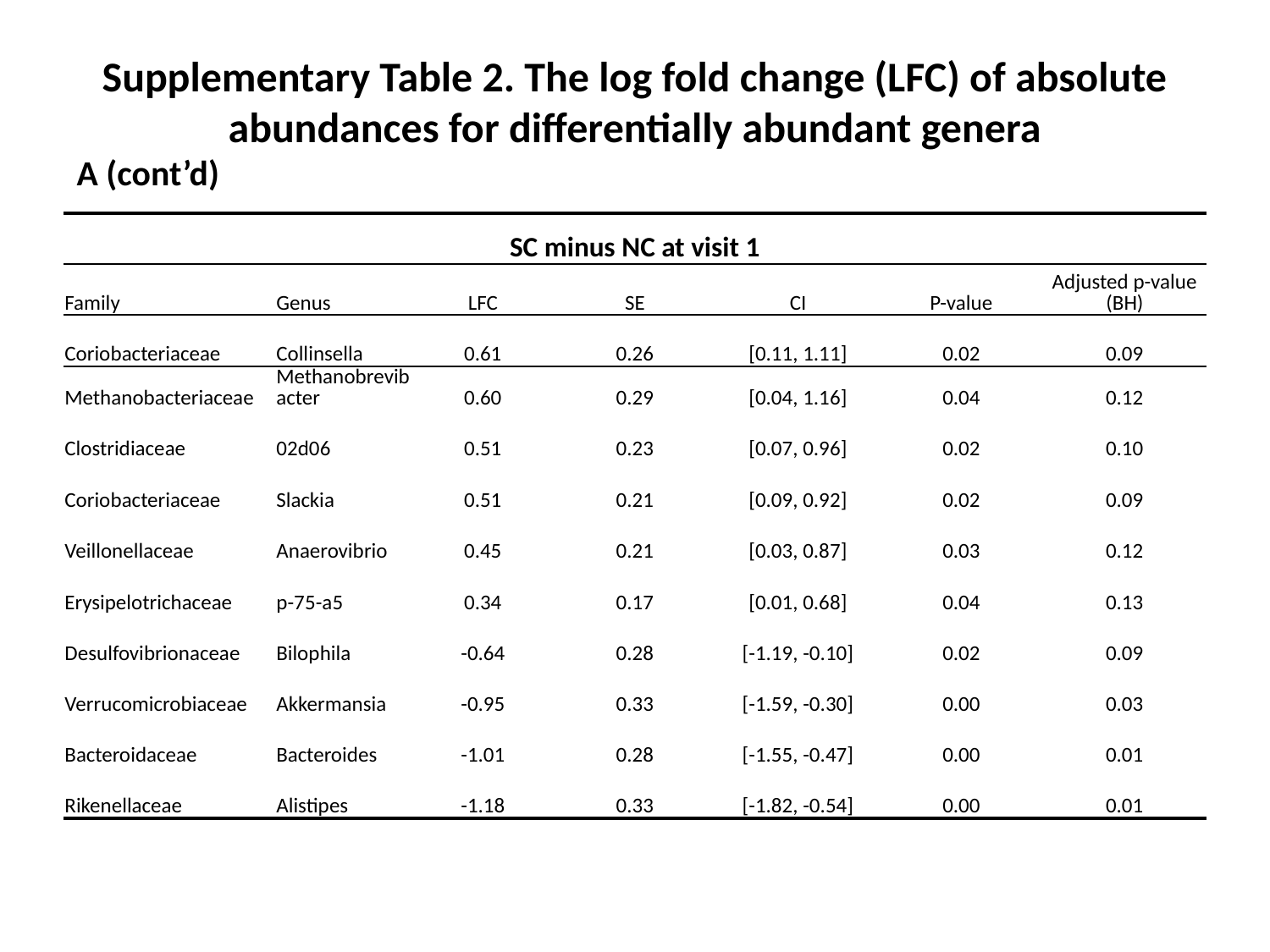

# Supplementary Table 2. The log fold change (LFC) of absolute abundances for differentially abundant genera
A (cont’d)
| SC minus NC at visit 1 | | | | | | |
| --- | --- | --- | --- | --- | --- | --- |
| Family | Genus | LFC | SE | CI | P-value | Adjusted p-value (BH) |
| Coriobacteriaceae | Collinsella | 0.61 | 0.26 | [0.11, 1.11] | 0.02 | 0.09 |
| Methanobacteriaceae | Methanobrevibacter | 0.60 | 0.29 | [0.04, 1.16] | 0.04 | 0.12 |
| Clostridiaceae | 02d06 | 0.51 | 0.23 | [0.07, 0.96] | 0.02 | 0.10 |
| Coriobacteriaceae | Slackia | 0.51 | 0.21 | [0.09, 0.92] | 0.02 | 0.09 |
| Veillonellaceae | Anaerovibrio | 0.45 | 0.21 | [0.03, 0.87] | 0.03 | 0.12 |
| Erysipelotrichaceae | p-75-a5 | 0.34 | 0.17 | [0.01, 0.68] | 0.04 | 0.13 |
| Desulfovibrionaceae | Bilophila | -0.64 | 0.28 | [-1.19, -0.10] | 0.02 | 0.09 |
| Verrucomicrobiaceae | Akkermansia | -0.95 | 0.33 | [-1.59, -0.30] | 0.00 | 0.03 |
| Bacteroidaceae | Bacteroides | -1.01 | 0.28 | [-1.55, -0.47] | 0.00 | 0.01 |
| Rikenellaceae | Alistipes | -1.18 | 0.33 | [-1.82, -0.54] | 0.00 | 0.01 |

## Slide 8
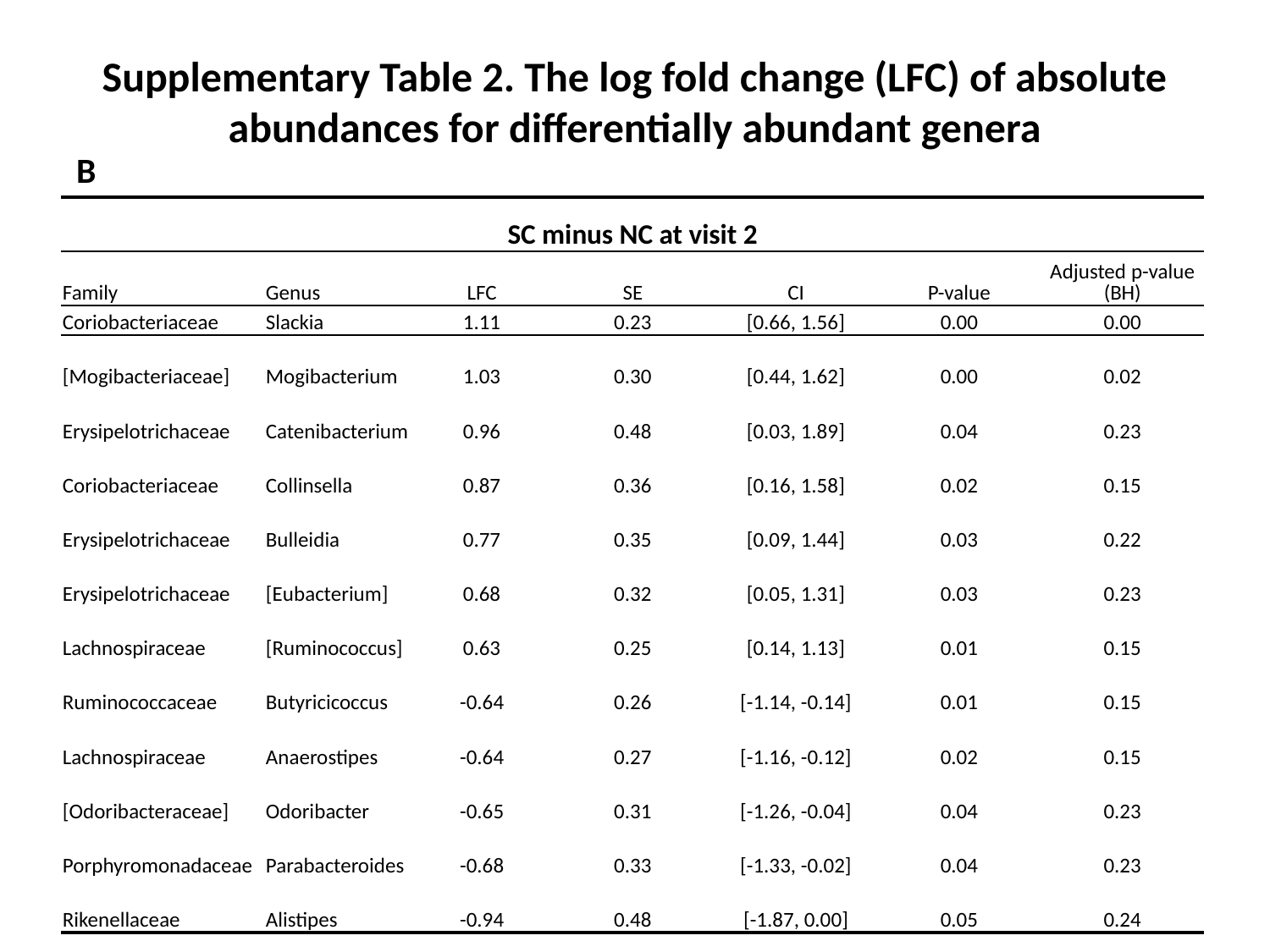

# Supplementary Table 2. The log fold change (LFC) of absolute abundances for differentially abundant genera
B
| SC minus NC at visit 2 | | | | | | |
| --- | --- | --- | --- | --- | --- | --- |
| Family | Genus | LFC | SE | CI | P-value | Adjusted p-value (BH) |
| Coriobacteriaceae | Slackia | 1.11 | 0.23 | [0.66, 1.56] | 0.00 | 0.00 |
| [Mogibacteriaceae] | Mogibacterium | 1.03 | 0.30 | [0.44, 1.62] | 0.00 | 0.02 |
| Erysipelotrichaceae | Catenibacterium | 0.96 | 0.48 | [0.03, 1.89] | 0.04 | 0.23 |
| Coriobacteriaceae | Collinsella | 0.87 | 0.36 | [0.16, 1.58] | 0.02 | 0.15 |
| Erysipelotrichaceae | Bulleidia | 0.77 | 0.35 | [0.09, 1.44] | 0.03 | 0.22 |
| Erysipelotrichaceae | [Eubacterium] | 0.68 | 0.32 | [0.05, 1.31] | 0.03 | 0.23 |
| Lachnospiraceae | [Ruminococcus] | 0.63 | 0.25 | [0.14, 1.13] | 0.01 | 0.15 |
| Ruminococcaceae | Butyricicoccus | -0.64 | 0.26 | [-1.14, -0.14] | 0.01 | 0.15 |
| Lachnospiraceae | Anaerostipes | -0.64 | 0.27 | [-1.16, -0.12] | 0.02 | 0.15 |
| [Odoribacteraceae] | Odoribacter | -0.65 | 0.31 | [-1.26, -0.04] | 0.04 | 0.23 |
| Porphyromonadaceae | Parabacteroides | -0.68 | 0.33 | [-1.33, -0.02] | 0.04 | 0.23 |
| Rikenellaceae | Alistipes | -0.94 | 0.48 | [-1.87, 0.00] | 0.05 | 0.24 |

## Slide 9
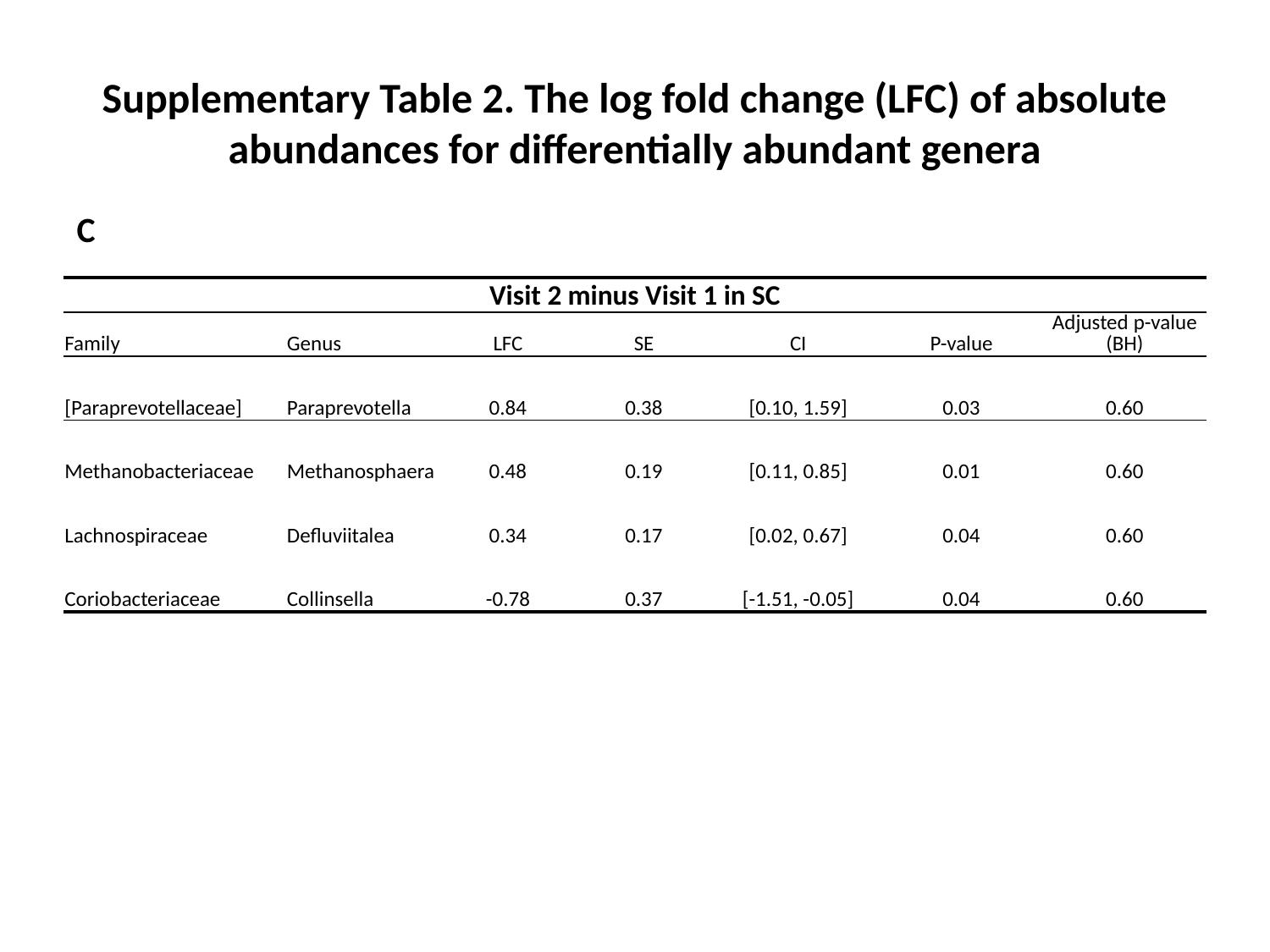

# Supplementary Table 2. The log fold change (LFC) of absolute abundances for differentially abundant genera
C
| Visit 2 minus Visit 1 in SC | | | | | | |
| --- | --- | --- | --- | --- | --- | --- |
| Family | Genus | LFC | SE | CI | P-value | Adjusted p-value (BH) |
| [Paraprevotellaceae] | Paraprevotella | 0.84 | 0.38 | [0.10, 1.59] | 0.03 | 0.60 |
| Methanobacteriaceae | Methanosphaera | 0.48 | 0.19 | [0.11, 0.85] | 0.01 | 0.60 |
| Lachnospiraceae | Defluviitalea | 0.34 | 0.17 | [0.02, 0.67] | 0.04 | 0.60 |
| Coriobacteriaceae | Collinsella | -0.78 | 0.37 | [-1.51, -0.05] | 0.04 | 0.60 |

## Slide 10
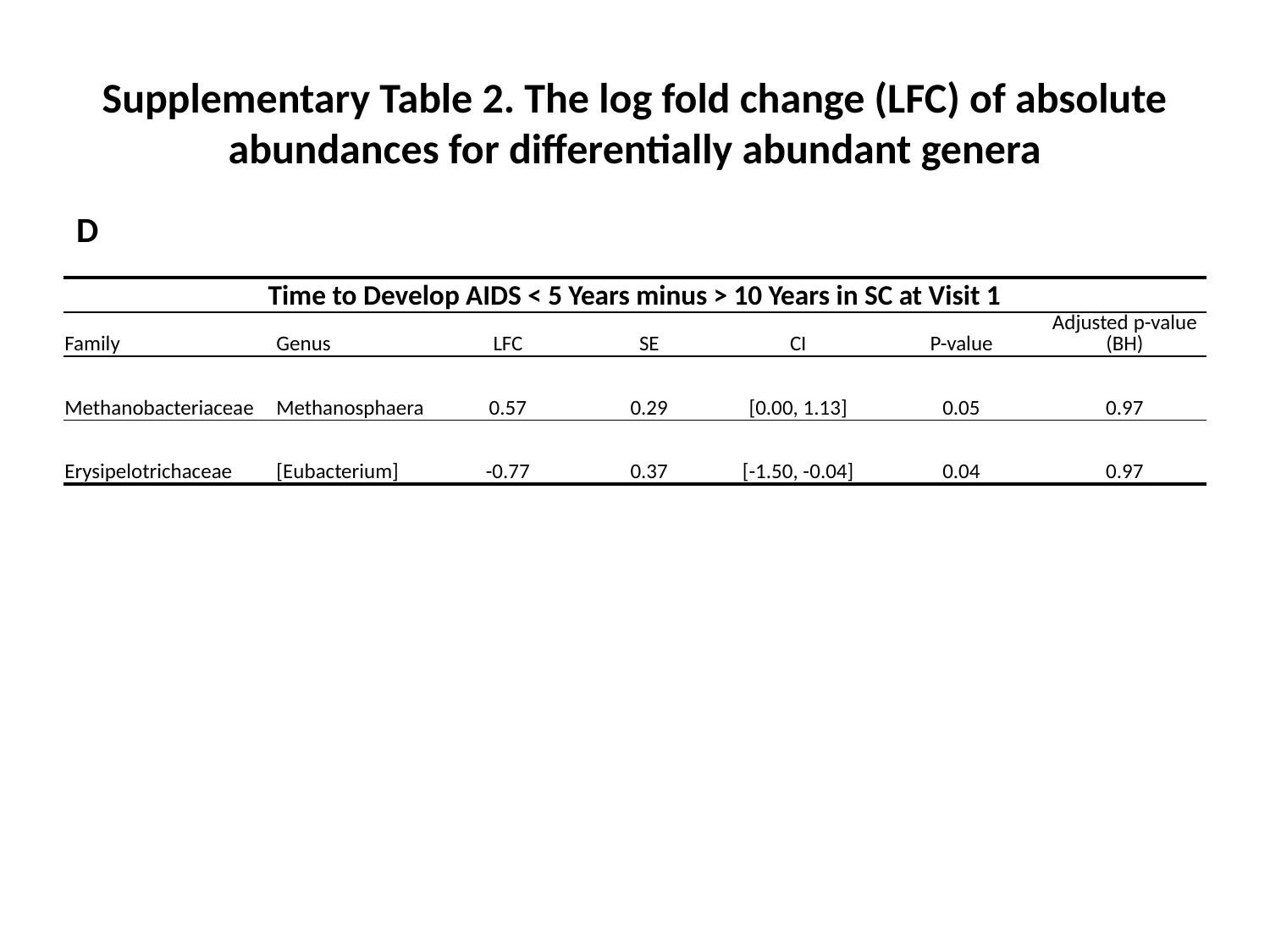

# Supplementary Table 2. The log fold change (LFC) of absolute abundances for differentially abundant genera
D
| Time to Develop AIDS < 5 Years minus > 10 Years in SC at Visit 1 | | | | | | |
| --- | --- | --- | --- | --- | --- | --- |
| Family | Genus | LFC | SE | CI | P-value | Adjusted p-value (BH) |
| Methanobacteriaceae | Methanosphaera | 0.57 | 0.29 | [0.00, 1.13] | 0.05 | 0.97 |
| Erysipelotrichaceae | [Eubacterium] | -0.77 | 0.37 | [-1.50, -0.04] | 0.04 | 0.97 |

## Slide 11
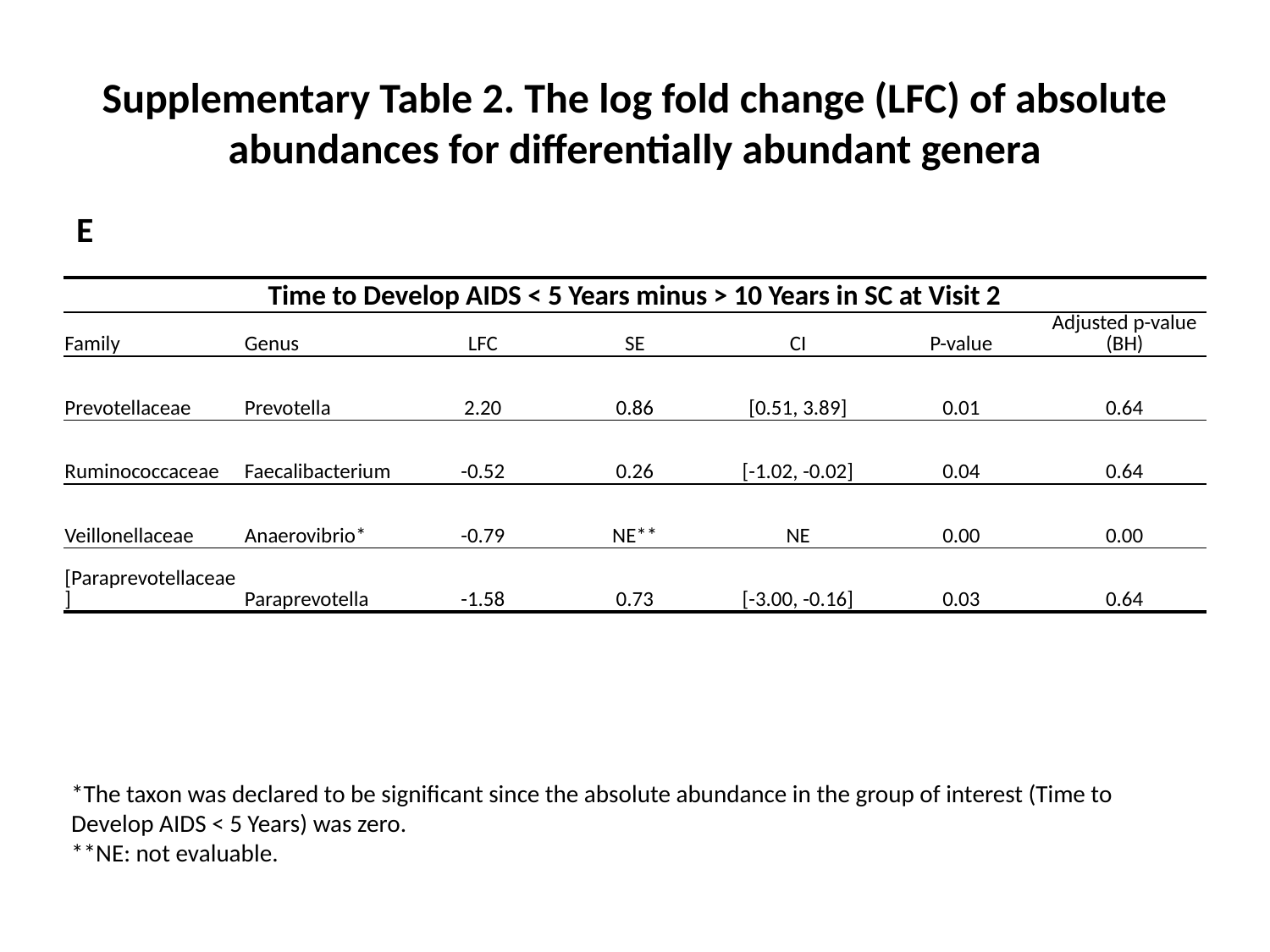

# Supplementary Table 2. The log fold change (LFC) of absolute abundances for differentially abundant genera
E
| Time to Develop AIDS < 5 Years minus > 10 Years in SC at Visit 2 | | | | | | |
| --- | --- | --- | --- | --- | --- | --- |
| Family | Genus | LFC | SE | CI | P-value | Adjusted p-value (BH) |
| Prevotellaceae | Prevotella | 2.20 | 0.86 | [0.51, 3.89] | 0.01 | 0.64 |
| Ruminococcaceae | Faecalibacterium | -0.52 | 0.26 | [-1.02, -0.02] | 0.04 | 0.64 |
| Veillonellaceae | Anaerovibrio\* | -0.79 | NE\*\* | NE | 0.00 | 0.00 |
| [Paraprevotellaceae] | Paraprevotella | -1.58 | 0.73 | [-3.00, -0.16] | 0.03 | 0.64 |
*The taxon was declared to be significant since the absolute abundance in the group of interest (Time to Develop AIDS < 5 Years) was zero.
**NE: not evaluable.

## Slide 12
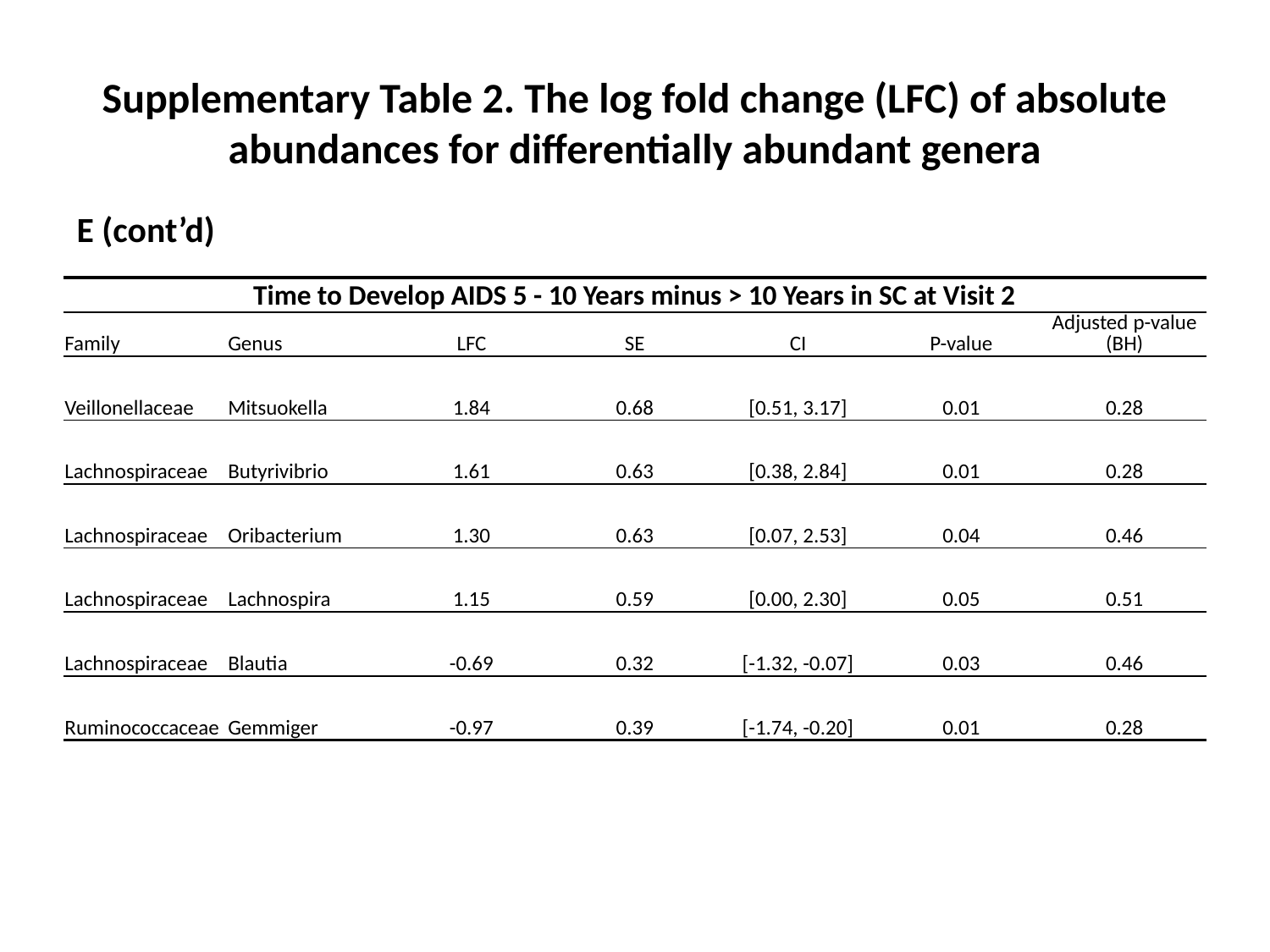

# Supplementary Table 2. The log fold change (LFC) of absolute abundances for differentially abundant genera
E (cont’d)
| Time to Develop AIDS 5 - 10 Years minus > 10 Years in SC at Visit 2 | | | | | | |
| --- | --- | --- | --- | --- | --- | --- |
| Family | Genus | LFC | SE | CI | P-value | Adjusted p-value (BH) |
| Veillonellaceae | Mitsuokella | 1.84 | 0.68 | [0.51, 3.17] | 0.01 | 0.28 |
| Lachnospiraceae | Butyrivibrio | 1.61 | 0.63 | [0.38, 2.84] | 0.01 | 0.28 |
| Lachnospiraceae | Oribacterium | 1.30 | 0.63 | [0.07, 2.53] | 0.04 | 0.46 |
| Lachnospiraceae | Lachnospira | 1.15 | 0.59 | [0.00, 2.30] | 0.05 | 0.51 |
| Lachnospiraceae | Blautia | -0.69 | 0.32 | [-1.32, -0.07] | 0.03 | 0.46 |
| Ruminococcaceae | Gemmiger | -0.97 | 0.39 | [-1.74, -0.20] | 0.01 | 0.28 |

## Slide 13
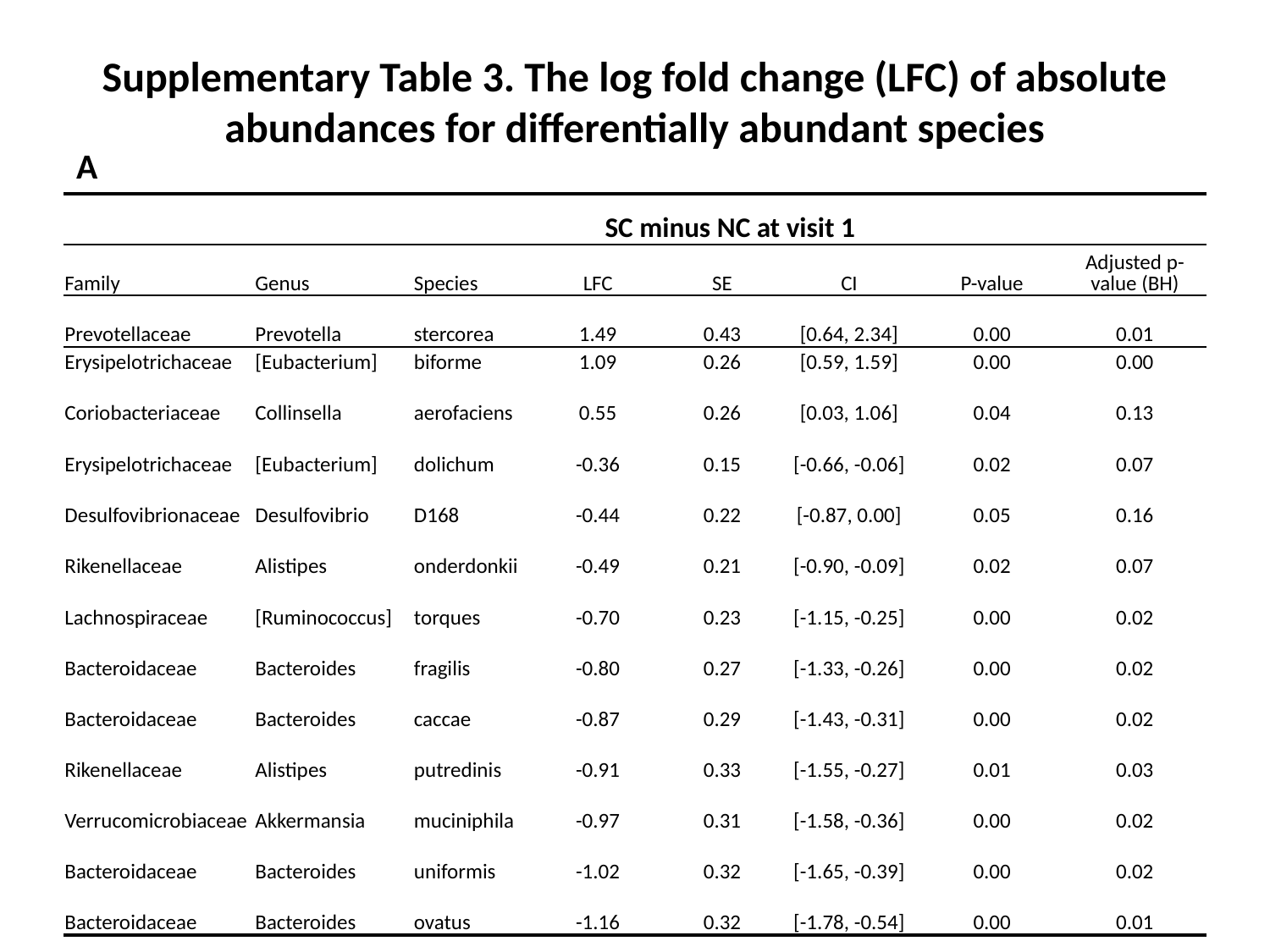

# Supplementary Table 3. The log fold change (LFC) of absolute abundances for differentially abundant species
A
| | SC minus NC at visit 1 | | | | | | |
| --- | --- | --- | --- | --- | --- | --- | --- |
| Family | Genus | Species | LFC | SE | CI | P-value | Adjusted p-value (BH) |
| Prevotellaceae | Prevotella | stercorea | 1.49 | 0.43 | [0.64, 2.34] | 0.00 | 0.01 |
| Erysipelotrichaceae | [Eubacterium] | biforme | 1.09 | 0.26 | [0.59, 1.59] | 0.00 | 0.00 |
| Coriobacteriaceae | Collinsella | aerofaciens | 0.55 | 0.26 | [0.03, 1.06] | 0.04 | 0.13 |
| Erysipelotrichaceae | [Eubacterium] | dolichum | -0.36 | 0.15 | [-0.66, -0.06] | 0.02 | 0.07 |
| Desulfovibrionaceae | Desulfovibrio | D168 | -0.44 | 0.22 | [-0.87, 0.00] | 0.05 | 0.16 |
| Rikenellaceae | Alistipes | onderdonkii | -0.49 | 0.21 | [-0.90, -0.09] | 0.02 | 0.07 |
| Lachnospiraceae | [Ruminococcus] | torques | -0.70 | 0.23 | [-1.15, -0.25] | 0.00 | 0.02 |
| Bacteroidaceae | Bacteroides | fragilis | -0.80 | 0.27 | [-1.33, -0.26] | 0.00 | 0.02 |
| Bacteroidaceae | Bacteroides | caccae | -0.87 | 0.29 | [-1.43, -0.31] | 0.00 | 0.02 |
| Rikenellaceae | Alistipes | putredinis | -0.91 | 0.33 | [-1.55, -0.27] | 0.01 | 0.03 |
| Verrucomicrobiaceae | Akkermansia | muciniphila | -0.97 | 0.31 | [-1.58, -0.36] | 0.00 | 0.02 |
| Bacteroidaceae | Bacteroides | uniformis | -1.02 | 0.32 | [-1.65, -0.39] | 0.00 | 0.02 |
| Bacteroidaceae | Bacteroides | ovatus | -1.16 | 0.32 | [-1.78, -0.54] | 0.00 | 0.01 |

## Slide 14
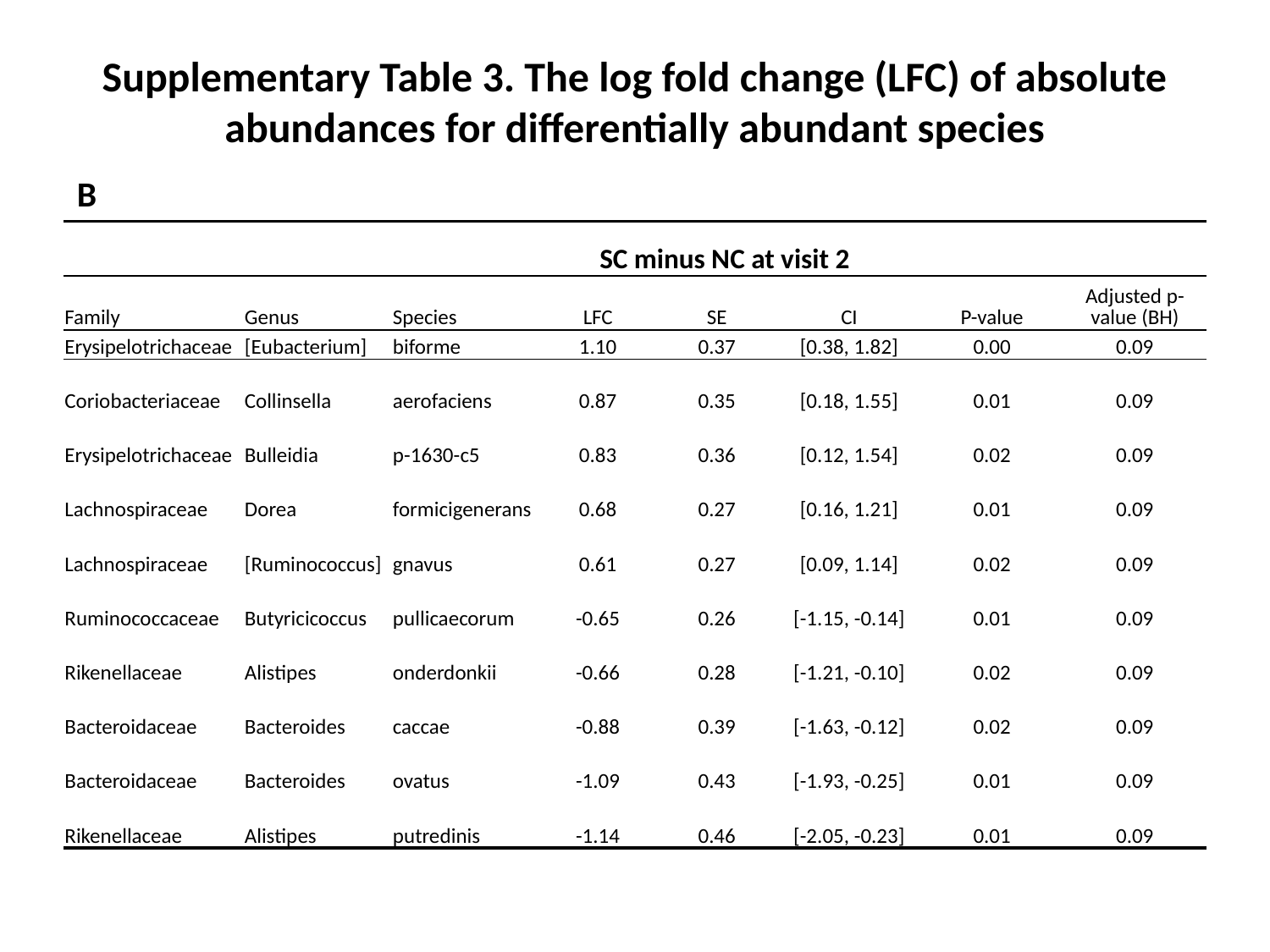

# Supplementary Table 3. The log fold change (LFC) of absolute abundances for differentially abundant species
B
| | SC minus NC at visit 2 | | | | | | |
| --- | --- | --- | --- | --- | --- | --- | --- |
| Family | Genus | Species | LFC | SE | CI | P-value | Adjusted p-value (BH) |
| Erysipelotrichaceae | [Eubacterium] | biforme | 1.10 | 0.37 | [0.38, 1.82] | 0.00 | 0.09 |
| Coriobacteriaceae | Collinsella | aerofaciens | 0.87 | 0.35 | [0.18, 1.55] | 0.01 | 0.09 |
| Erysipelotrichaceae | Bulleidia | p-1630-c5 | 0.83 | 0.36 | [0.12, 1.54] | 0.02 | 0.09 |
| Lachnospiraceae | Dorea | formicigenerans | 0.68 | 0.27 | [0.16, 1.21] | 0.01 | 0.09 |
| Lachnospiraceae | [Ruminococcus] | gnavus | 0.61 | 0.27 | [0.09, 1.14] | 0.02 | 0.09 |
| Ruminococcaceae | Butyricicoccus | pullicaecorum | -0.65 | 0.26 | [-1.15, -0.14] | 0.01 | 0.09 |
| Rikenellaceae | Alistipes | onderdonkii | -0.66 | 0.28 | [-1.21, -0.10] | 0.02 | 0.09 |
| Bacteroidaceae | Bacteroides | caccae | -0.88 | 0.39 | [-1.63, -0.12] | 0.02 | 0.09 |
| Bacteroidaceae | Bacteroides | ovatus | -1.09 | 0.43 | [-1.93, -0.25] | 0.01 | 0.09 |
| Rikenellaceae | Alistipes | putredinis | -1.14 | 0.46 | [-2.05, -0.23] | 0.01 | 0.09 |

## Slide 15
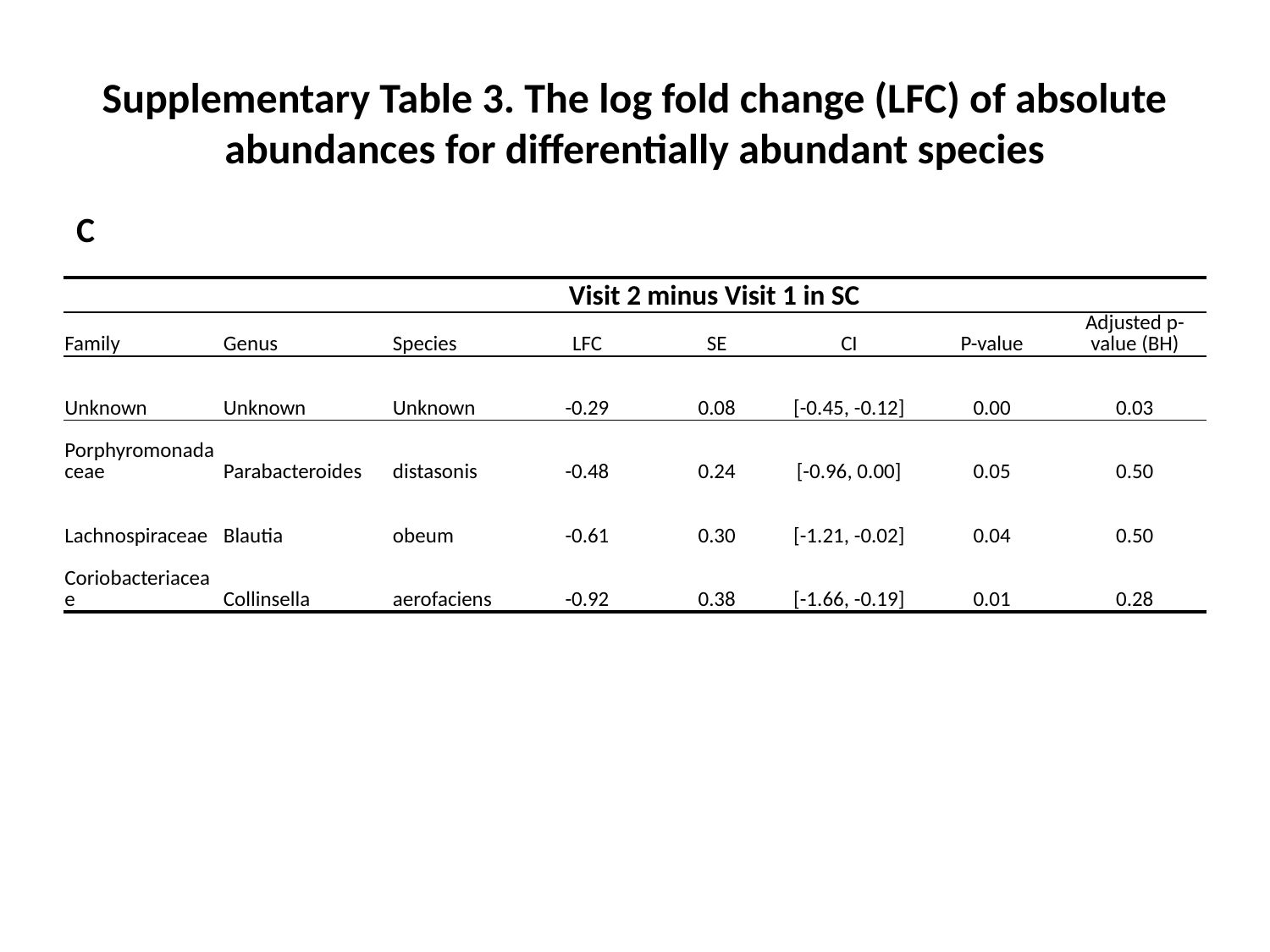

# Supplementary Table 3. The log fold change (LFC) of absolute abundances for differentially abundant species
C
| | Visit 2 minus Visit 1 in SC | | | | | | |
| --- | --- | --- | --- | --- | --- | --- | --- |
| Family | Genus | Species | LFC | SE | CI | P-value | Adjusted p-value (BH) |
| Unknown | Unknown | Unknown | -0.29 | 0.08 | [-0.45, -0.12] | 0.00 | 0.03 |
| Porphyromonadaceae | Parabacteroides | distasonis | -0.48 | 0.24 | [-0.96, 0.00] | 0.05 | 0.50 |
| Lachnospiraceae | Blautia | obeum | -0.61 | 0.30 | [-1.21, -0.02] | 0.04 | 0.50 |
| Coriobacteriaceae | Collinsella | aerofaciens | -0.92 | 0.38 | [-1.66, -0.19] | 0.01 | 0.28 |

## Slide 16
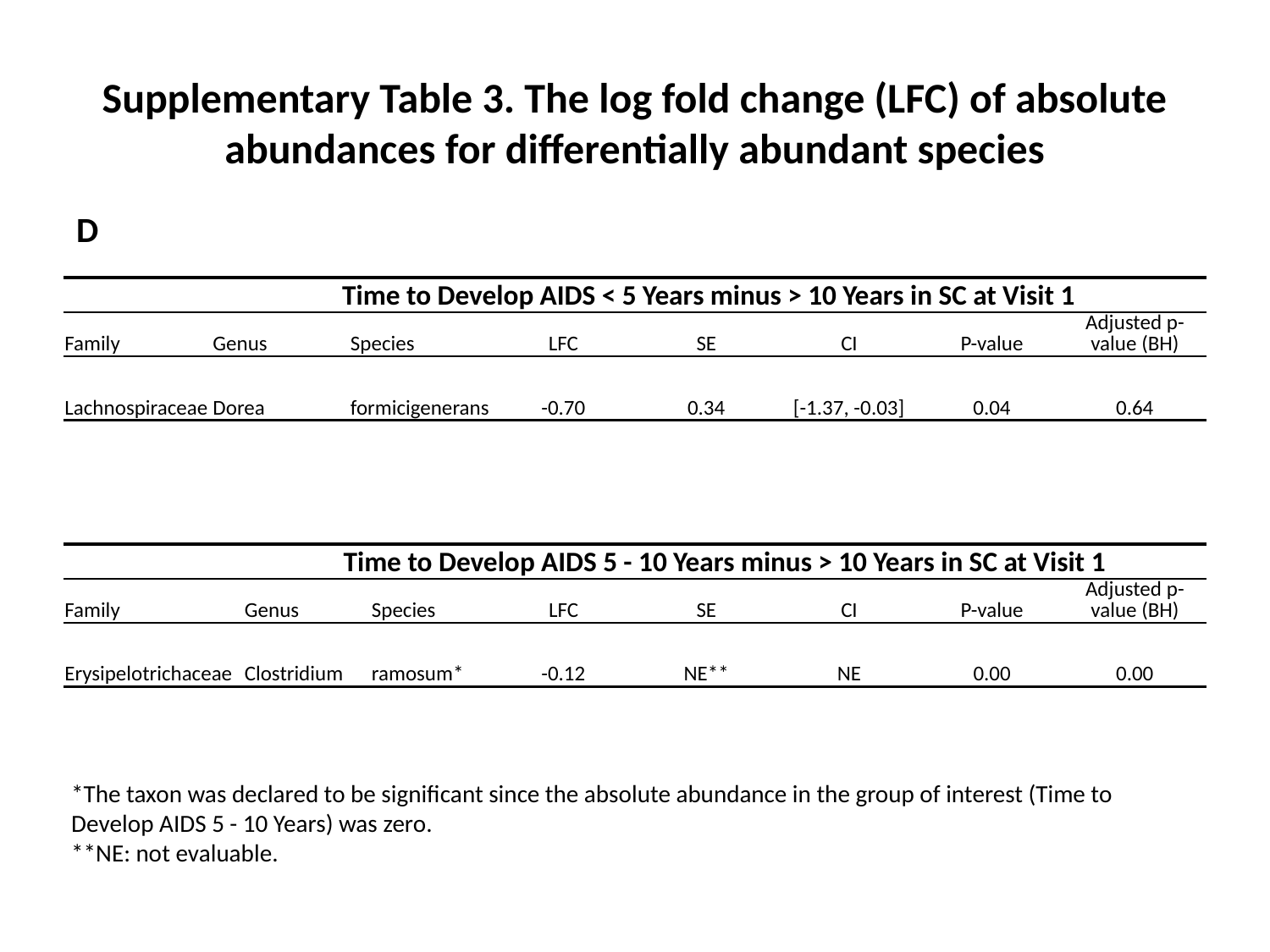

# Supplementary Table 3. The log fold change (LFC) of absolute abundances for differentially abundant species
D
| | Time to Develop AIDS < 5 Years minus > 10 Years in SC at Visit 1 | | | | | | |
| --- | --- | --- | --- | --- | --- | --- | --- |
| Family | Genus | Species | LFC | SE | CI | P-value | Adjusted p-value (BH) |
| Lachnospiraceae | Dorea | formicigenerans | -0.70 | 0.34 | [-1.37, -0.03] | 0.04 | 0.64 |
| | Time to Develop AIDS 5 - 10 Years minus > 10 Years in SC at Visit 1 | | | | | | |
| --- | --- | --- | --- | --- | --- | --- | --- |
| Family | Genus | Species | LFC | SE | CI | P-value | Adjusted p-value (BH) |
| Erysipelotrichaceae | Clostridium | ramosum\* | -0.12 | NE\*\* | NE | 0.00 | 0.00 |
*The taxon was declared to be significant since the absolute abundance in the group of interest (Time to Develop AIDS 5 - 10 Years) was zero.
**NE: not evaluable.

## Slide 17
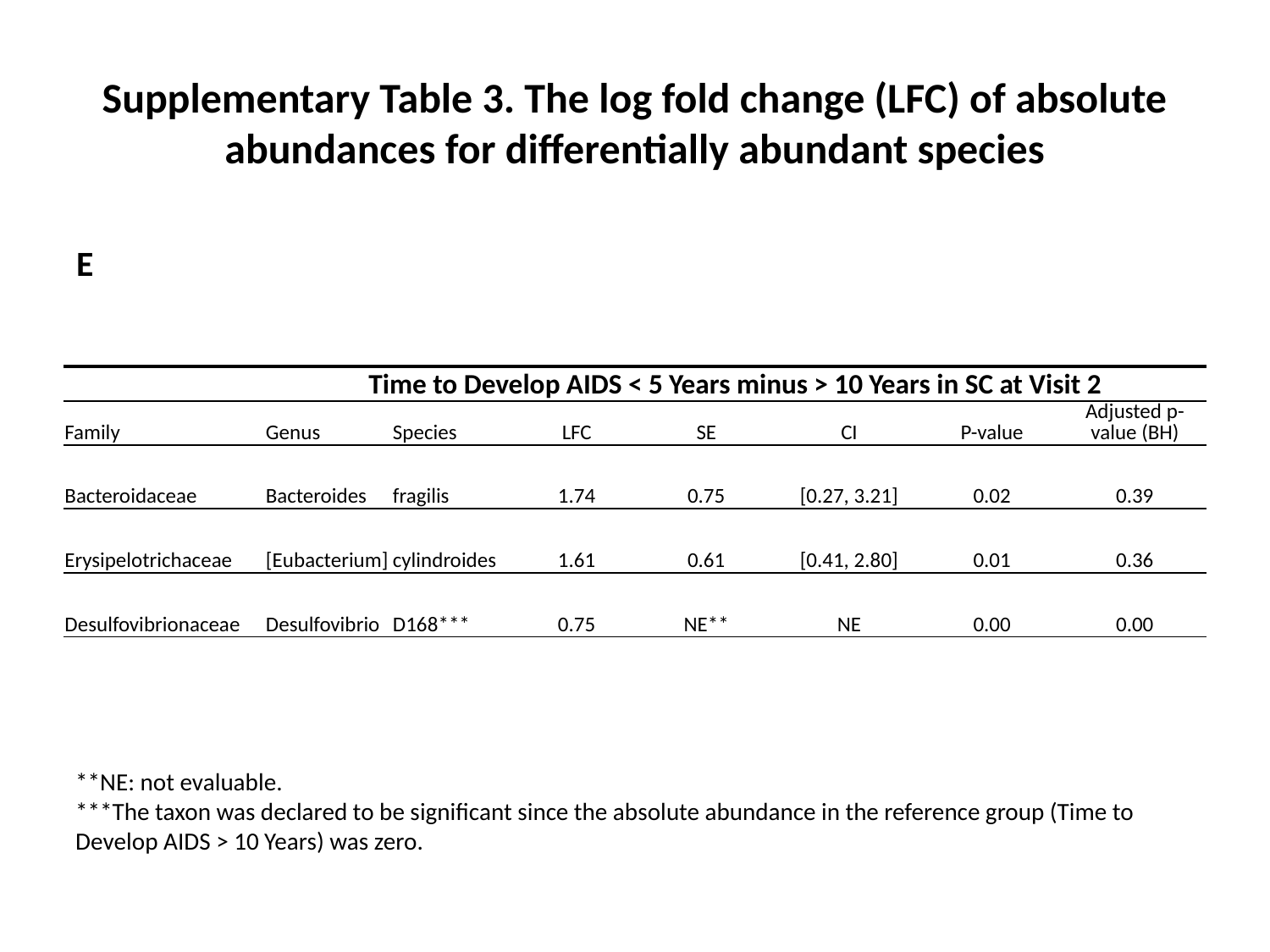

# Supplementary Table 3. The log fold change (LFC) of absolute abundances for differentially abundant species
E
| | Time to Develop AIDS < 5 Years minus > 10 Years in SC at Visit 2 | | | | | | |
| --- | --- | --- | --- | --- | --- | --- | --- |
| Family | Genus | Species | LFC | SE | CI | P-value | Adjusted p-value (BH) |
| Bacteroidaceae | Bacteroides | fragilis | 1.74 | 0.75 | [0.27, 3.21] | 0.02 | 0.39 |
| Erysipelotrichaceae | [Eubacterium] | cylindroides | 1.61 | 0.61 | [0.41, 2.80] | 0.01 | 0.36 |
| Desulfovibrionaceae | Desulfovibrio | D168\*\*\* | 0.75 | NE\*\* | NE | 0.00 | 0.00 |
**NE: not evaluable.
***The taxon was declared to be significant since the absolute abundance in the reference group (Time to Develop AIDS > 10 Years) was zero.

## Slide 18
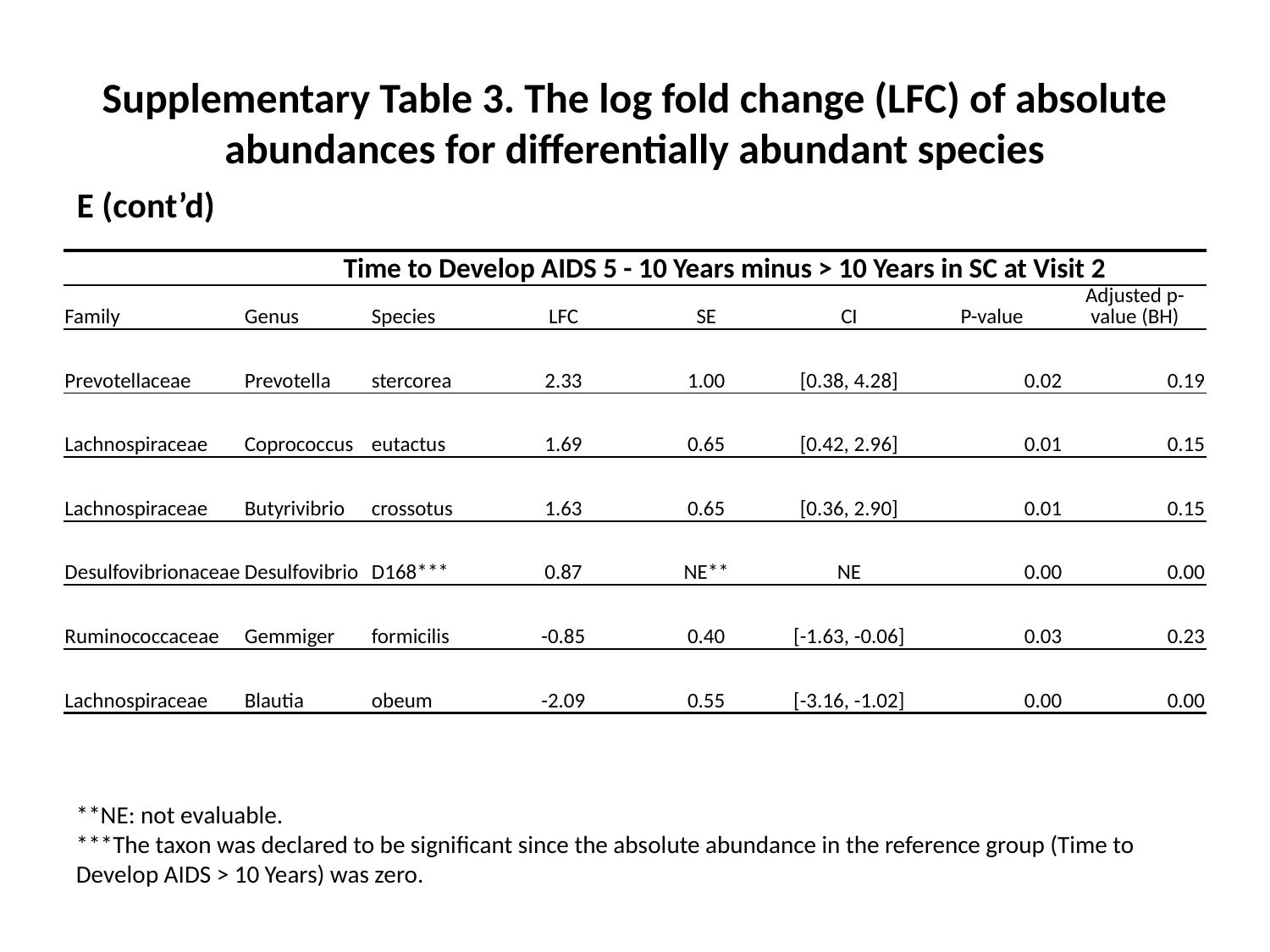

# Supplementary Table 3. The log fold change (LFC) of absolute abundances for differentially abundant species
E (cont’d)
| | Time to Develop AIDS 5 - 10 Years minus > 10 Years in SC at Visit 2 | | | | | | |
| --- | --- | --- | --- | --- | --- | --- | --- |
| Family | Genus | Species | LFC | SE | CI | P-value | Adjusted p-value (BH) |
| Prevotellaceae | Prevotella | stercorea | 2.33 | 1.00 | [0.38, 4.28] | 0.02 | 0.19 |
| Lachnospiraceae | Coprococcus | eutactus | 1.69 | 0.65 | [0.42, 2.96] | 0.01 | 0.15 |
| Lachnospiraceae | Butyrivibrio | crossotus | 1.63 | 0.65 | [0.36, 2.90] | 0.01 | 0.15 |
| Desulfovibrionaceae | Desulfovibrio | D168\*\*\* | 0.87 | NE\*\* | NE | 0.00 | 0.00 |
| Ruminococcaceae | Gemmiger | formicilis | -0.85 | 0.40 | [-1.63, -0.06] | 0.03 | 0.23 |
| Lachnospiraceae | Blautia | obeum | -2.09 | 0.55 | [-3.16, -1.02] | 0.00 | 0.00 |
**NE: not evaluable.
***The taxon was declared to be significant since the absolute abundance in the reference group (Time to Develop AIDS > 10 Years) was zero.
